# Tiling array study of MNNG treated *Escherichia coli* reveals a widespread transcriptional response

**DOI:** 10.1038/srep03053

**Published:** 2013-10-25

**Authors:** James A. Booth, Gard O. S. Thomassen, Alexander D. Rowe, Ragnhild Weel-Sneve, Karin Lagesen, Knut I. Kristiansen, Magnar Bjørås, Torbjørn Rognes, Jessica M. Lindvall

**Affiliations:** 1 Centre for Molecular Biology and Neuroscience (CMBN) and Department of Microbiology, Oslo University Hospital, Rikshospitalet, PO Box 4950 Nydalen, NO-0424 Oslo, Norway; 2 Department of Microbiology, University of Oslo, PO Box 4950 Nydalen, NO-0424 Oslo, Norway; 3 Department of Informatics, University of Oslo, PO Box 1080 Blindern, NO-0316 Oslo, Norway; 4 Department of Medical Biochemistry, University of Oslo, PO Box 4950 Nydalen, NO-0424 Oslo, Norway; 5 These authors contributed equally to this work.; 6 Current address: University Center for Information Technology, University of Oslo, PO Box 1059 Blindern, NO-0316 Oslo.; 7 Current address: Department of Biosciences and Nutrition, Karolinska Institutet, Novum, SE-141 83 Huddinge, Sweden.

## Abstract

The alkylating agent *N*-methyl-*N*'-nitro-*N*-nitrosoguanidine (MNNG) is known to trigger the adaptive response by inducing the *ada*-regulon – consisting of three DNA repair enzymes Ada, AlkB, AlkA and the enigmatic AidB. We have applied custom designed tiling arrays to study transcriptional changes in *Escherichia coli* following a MNNG challenge. Along with the expected upregulation of the adaptive response genes (*ada*, *alkA* and *alkB*), we identified a number of differentially expressed transcripts, both novel and annotated. This indicates a wider regulatory response than previously documented. There were 250 differentially-expressed and 2275 similarly-expressed unannotated transcripts. We found novel upregulation of several stress-induced transcripts, including the SOS inducible genes *recN* and *tisAB*, indicating a novel role for these genes in alkylation repair. Furthermore, the *ada*-regulon A and B boxes were found to be insufficient to explain the regulation of the adaptive response genes after MNNG exposure, suggesting that additional regulatory elements must be involved.

Alkylating agents are present and generated both intra- and extracellularly. Such agents react with DNA and can subsequently give rise to mutations that can lead to cell death^1^,^2^. The alkylating agent *N*-methyl-*N'*-nitro-*N*-nitrosoguanidine (MNNG) reacts with DNA to produce different *O*-alkylated and *N*-alkylated DNA lesions^3^. After exposure of *Escherichia coli* (*E. coli*) to non-lethal doses of MNNG, transcription of the *ada*, *alkB*, *alkA* and *aidB* genes are induced under control of the *ada*-regulon. This stress response to alkylating agents is termed the adaptive response^2^,^4^. The Ada protein is a DNA methyltransferase that removes methyl groups from the premutagenic lesions *O*^6^-methylguanine and *O*^4^-methylthymine^2^. The AlkB protein repairs 1-methyladenine and 3-methylcytosine in DNA by oxidative demethylation^5^,^6^. The DNA glycosylase AlkA catalyses the removal of several methylated base lesions^7^. Flavin adenine dinucleotide (FAD) containing AidB is involved in reducing the mutagenic effects of MNNG. It has been shown to potentially act at specific highly expressed genes including various DNA repair and constitutively expressed genes, but the mechanism of action is unknown^8^,^9^. In addition to the role of Ada in the repair of alkylated DNA, the Ada protein also regulates the *ada*-regulon^10^,^11^,^12^. When the Ada protein covalently transfers a methyl group from a methylated phosphotriester in the DNA backbone to its own *N*-terminal Cys38, Ada is converted into a transcriptional activator of the *ada*, *alkB*, *alkA* and *aidB* genes^10^.

Genome-tiling microarrays have enabled investigation of global expression patterns in organisms such as bacteria, mouse, human and yeast^13^,^14^,^15^,^16^,^17^,^18^, with or without a completely annotated genome. Investigations employing unbiased tiling of human chromosomes 21 and 22 have elegantly shown that large portions of the human genome are in fact transcribed^19^ and are not, as previously thought, simply “junk” areas. Whether these transcripts represent novel mRNAs or non-coding RNAs (ncRNAs)^20^ remains largely unclear. However, other more extensive datasets point towards the fact that these areas are of great importance^21^,^22^,^23^,^24^.

In traditional gene-probing microarrays, all probes targeting the same gene are assumed to give independent measures of the same RNA expression^15^,^25^,^26^. When applying a tiling strategy to an entire genome, the analysis should not depend on the annotation, as this would restrict the analysis to annotated genes only, and would make the analysis impossible in the absence of an annotation. Therefore, one of the major challenges for tiling array studies is to pinpoint transcriptional start and stop sites as well as to predict whether transcripts form long continuous stretches (typically genes) or short non-continuous strands of RNA (typically ncRNAs).

Recently, high-throughput RNA sequencing (RNA-Seq) has become available as an alternative approach to study transcription in great detail. Several RNA-Seq experiments have been performed on *E. coli*^27^,^28^,^29^,^30^, but to our knowledge no such experiments subjecting *E. coli* to MNNG have been published.

Here we present a comprehensive approach to high-throughput transcript discovery in *E. coli*. The study focuses mainly on intergenic regions, which in combination with an analysis of coding transcripts provides a more complete insight into the *E. coli* transcriptome. We have used a custom tiling microarray design strategy and a novel analysis method described earlier^31^. Furthermore, we have compared all findings to previously published *E. coli* transcriptome and proteome experiments^26^,^32^,^33^,^34^. This meta-analysis approach serves as a verification strategy as well as providing more insight into the data. Several of the differentially expressed transcripts have been verified by RT-qPCR. In addition, we have investigated the upstream sequences of all differentially expressed annotated genes aiming to identify the correlation between the reported *ada*-regulon box A and box B regulatory sequences^10^ and the gene regulation after MNNG exposure. Through the experimental identification of expressed or suppressed transcripts due to MNNG treatment, this study provide the basis for focused studies to subsequently uncover the functions and relevance of transcripts induced by methylation, e.g. *recN*, *tisAB*, *hmp*, *iraP* and *gcvT*.

## Results

In this work we have used a custom designed tiling array and three separate analysis approaches (see Methods and Thomassen, et al.^31^ for details) to characterize the transcriptional changes following MNNG treatment of *E. coli*. Details about the experiments, raw data as well as several tables of results from this study not included here, have been deposited at the NCBI Gene Expression Omnibus (GEO) public repository available online at www.ncbi.nlm.nih.gov/geo with GEO accession GSE13830. We have previously utilized the same tiling arrays and applied the same analysis methods in a similar experiment where *E. coli* was exposed to UV, and further information and examples can be found in that paper^35^.

### Change in expression of annotated transcripts after MNNG treatment

**Figure 1:**
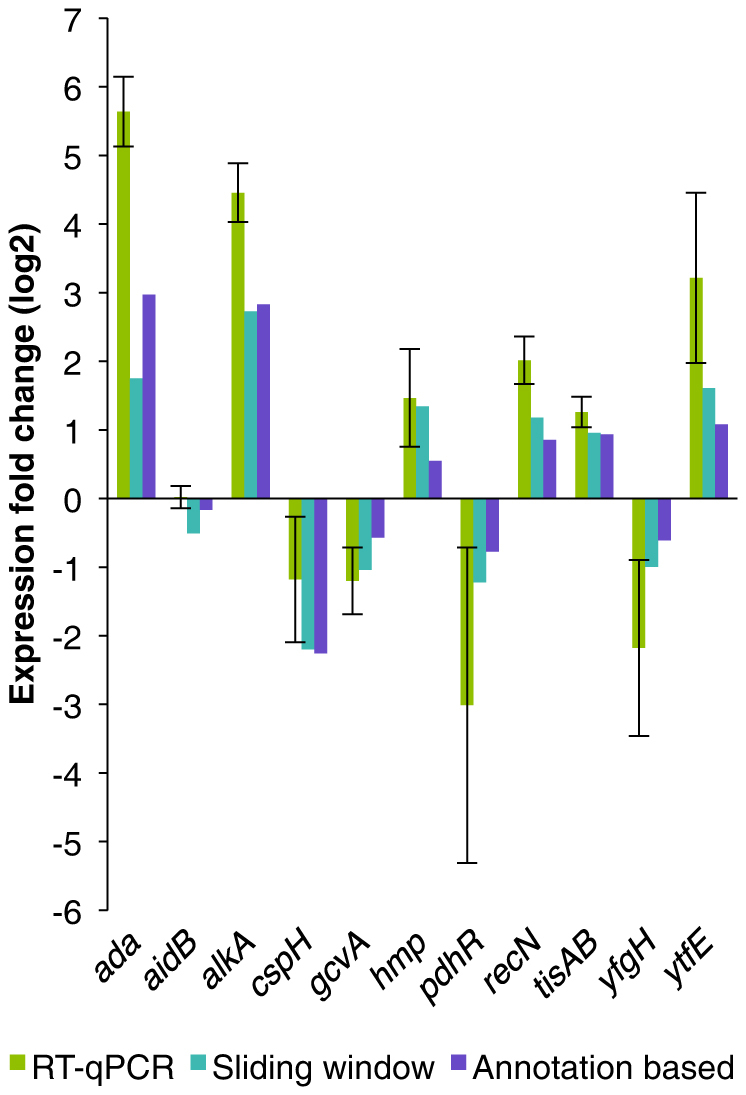
RT-qPCR verification of the expression of selected genes. Selected differentially expressed genes identified by the tiling array data were verified by RT-qPCR. The base 2 logarithms of the fold changes of expression are shown on the y-axis.

Initially, we wished to observe the number of annotated genes that were induced or repressed in response to MNNG treatment. Additionally, we were interested in the transcriptional changes of the genes known to be involved in the adaptive response. The annotation based analysis algorithm (see Methods and Thomassen, et al.^31^ for details) resulted in 185 differentially expressed protein coding genes (14 up- and 171 down-regulated) and 14 ncRNAs, which were all found to be downregulated following MNNG treatment. As expected, genes such as *ada*, *alkA* and *alkB*, which are known members of the adaptive response^2^,^4^, were strongly induced after MNNG exposure. Interestingly, *aidB* did not show any differential expression after MNNG stimulation, however, it has previously been shown to be only weakly induced by low concentrations of MNNG^36^. This finding was also verified by RT-qPCR experiments (Fig. 1). Of note is a study by Baek, et al.^32^ of the transcriptomic and proteomic changes following treatment of *E. coli* K-12 with the alkylating agent methyl-methanesulfonate (MMS) which reports only a minor *aidB* response after exposure, but a full response from the other adaptive response components. In this respect several studies have shown that MMS induces *aidB* to a greater extent than MNNG when compared to the induction of the other *ada*-regulon genes^36^,^37^. Five of the 185 differentially regulated protein coding genes (*yqiI*, *ymfA*, *yjbM*, *ybdO* and *ydfV*) were only detected in the reference transcriptome, not appearing in the MNNG induced transcriptome, and were thus defined as repressed in response to MNNG exposure. A total of 3752 out of the 4263 annotated transcripts, corresponding to 88%, were found to be similarly expressed in the two conditions, i.e. signal levels above background with no significant differential expression between the MNNG exposed and the reference sample.

### Identification of novel transcripts

**Figure 2:**
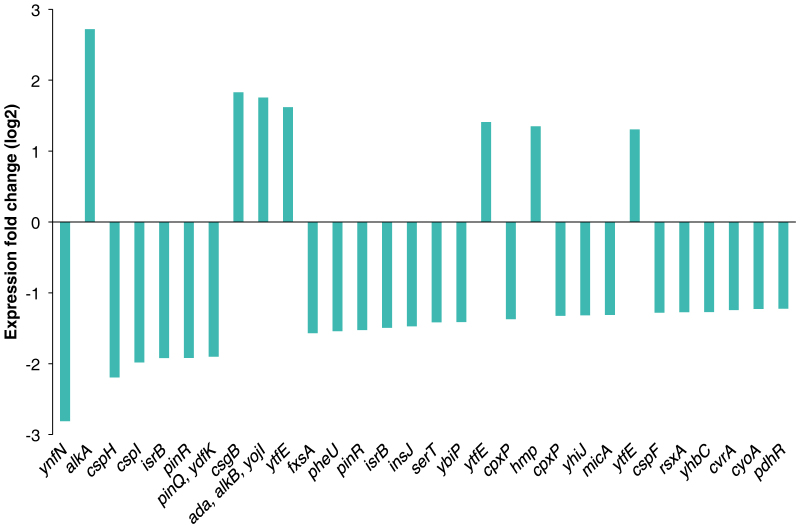
Transcripts with the largest change in magnitude of expression, in decreasing order from the left, of *E. coli* cells subjected to MNNG. The base 2 logarithms of the fold changes of expression are shown on the y-axis. Transcripts were detected using the "sliding window" method, and the gene names indicated are those of the annotated *E. coli* genes that overlap with the transcripts detected.

**Figure 3:**
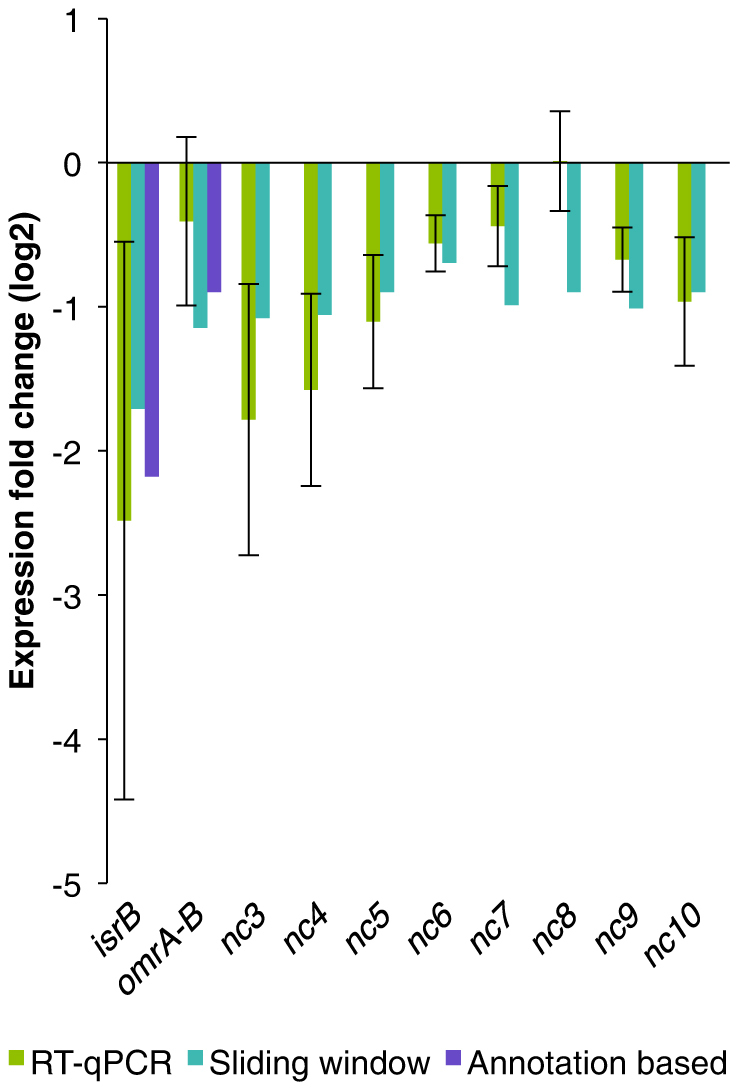
RT-qPCR verification of 10 differentially expressed ncRNA regions. Here, the fold change of eight modulated ncRNA candidates and two annotated ncRNAs is presented. ncRNA candidates nc3, nc4, nc6 and nc10 have been previously predicted whilst nc5, nc7, nc8 and nc9 are completely novel ncRNA candidates.

The sliding window algorithm detected significantly more transcription than the present annotation suggests (Table 1). Several of the detected transcripts showed a remarkably higher expression level and/or transcript length compared to the annotation based algorithm. Both the sliding window algorithm and the annotation-based algorithm are described in detail by Thomassen, et al.^31^. The sliding window analysis approach predicts transcripts by identifying continuous genome segments with enhanced expression based on several probe measurements. Our UV study^35^ includes two examples of plots of probe intensities over genomic regions and the location of genes that are either predicted using the sliding window approach or previously annotated. In short, the nature of the sliding window algorithm enables non-overlapping parts of one annotated gene to be reported as differentially and similarly expressed, thus one annotated transcript might count as two or more separate transcripts. A total of 904 genomic regions were detected as differentially expressed using the sliding window method. Of these, 322 regions touch (overlapping by one or more nucleotide) a total of 241 annotated transcripts, including 17 ncRNAs. Fig. 2 shows the annotated transcripts with the most prominent expression changes. Some of the detected transcripts represent two or more non-consecutive parts of a single annotated transcript (*cpxP, isrB, pinR, ytfE*), whereas others span two or more adjacent annotated transcripts, identifying potential operons (*ada, alkB, yojI; pinQ, ydfK*). The 17 annotated differentially expressed ncRNAs were all downregulated in response to MNNG. Finally, 250 differentially expressed regions are suggested as novel (previously non-annotated) differentially expressed transcripts. The RT-qPCR verification results of two differentially expressed annotated ncRNAs (*isrB* and *omrA-B*) along with four previously *in silico* predicted (nc3, nc4, nc6 and nc10) and four entirely novel ncRNA candidates (nc5, nc7, nc8 and nc9) are shown in Fig. 3. Table 2 lists the 15 most differentially expressed unannotated transcripts. Notably, several of the transcripts are found rather close to each other (novel1, novel2 and novel12; novel 4 and novel5; novel7 and novel13). Transcripts denoted novel1, novel2 and novel12 are found in a prophage region (Qin).

In order to investigate whether our novel transcripts overlap with regions predicted as genes and if rho-independent terminator structures reside within their 250 downstream nucleotides, we applied Prodigal^38^ and Glimmer^39^ (gene finders, here optimized for short genes) and TransTerm^40^ (terminator finder). Of the 250 novel differentially expressed transcripts one is predicted by Glimmer *and* Prodigal *and* have a terminator, 12 are predicted by Glimmer *or* Prodigal *and* have a terminator while 52 transcripts have terminators but no overlapping gene predication. The percentage of novel transcripts overlapping gene predictions and terminator structures are similar for the 2275 novel similarly expressed transcripts. These prediction results are shown in full detail in the GEO submission (GSE 13830). The results are as expected as most candidates would have been annotated automatically as genes if they made a very good fit for current gene-prediction software.

Additionally, potential antisense transcripts (from the opposite strand of known genes) were observed for numerous genes. Previous studies have either left these regions unprobed^34^, or detected abundant antisense transcription^26^,^27^,^30^. The antisense signalling detected within our data correlates well with previous *E. coli* findings by Selinger, et al.^26^, and the amount of antisense transcription also correlates with the results from two yeast studies^41^,^42^. However, many of these antisense transcripts could potentially be false positives. Perocchi, et al.^43^ used a tiling array strategy on *Saccharomyces cerevisiae* and showed that about half of the detected antisense transcripts were, in fact, technical artefacts. They demonstrated that these artefacts could be avoided by adding actinomycin D during the labelling reaction. Actinomycin D selectively prevents second-strand cDNA synthesis during reverse transcription due to the specific inhibition of DNA-dependent, but not RNA-dependent, DNA-synthesis. Since our experimental setup was performed without Actinomycin D, our methodology may be prone to the same problem. Based on this knowledge we decided to remove all possible antisense transcripts from the analysis, as their validity cannot be established satisfactorily.

Notably, the total number of expressed nucleotides decreased by about 3% after MNNG treatment. Further, we noticed that the number of genes downregulated after MNNG treatment was three times as large as the number of upregulated genes. Table 3 presents an overview of the number of genomic regions and annotated genes detected as transcribed in the reference and MNNG treated dataset, respectively.

### MNNG induced regulation of responses outside the adaptive response network

**Figure 4:**
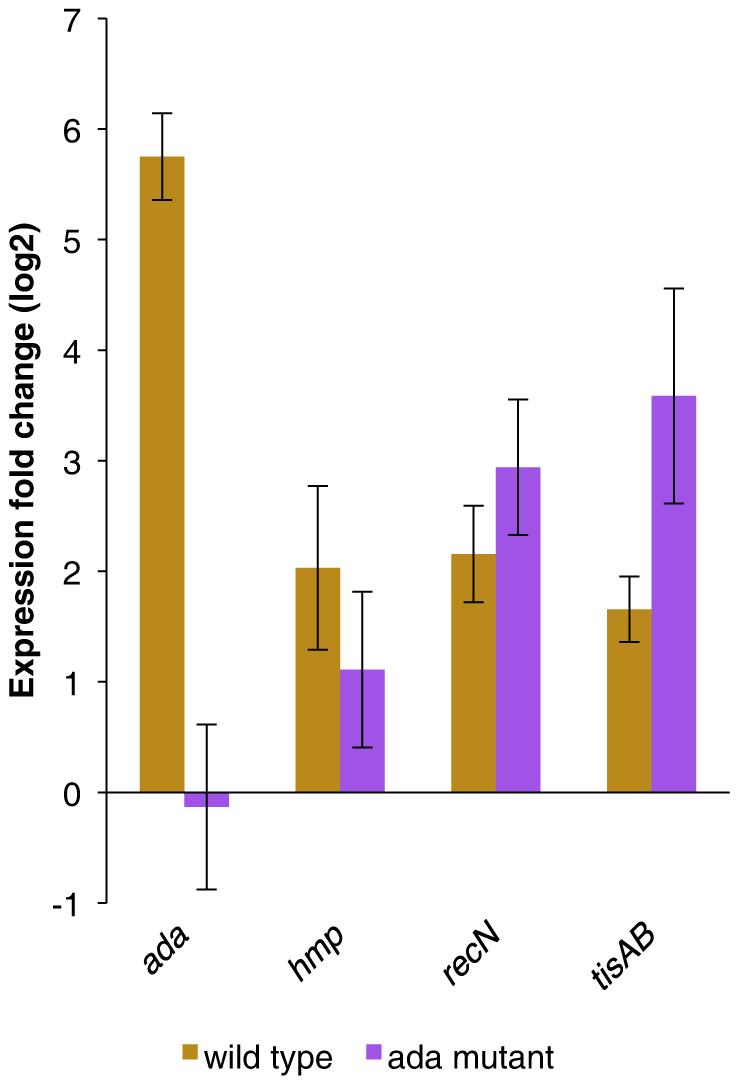
RT-qPCR for *ada*, *recN*, *tisAB* and *hmp* following MNNG stimulation in wild type and in the *ada* mutant strain BK2110. The mutant *E. coli* BK2110 strain is an *ada* mutant in AB1157 background^68^ which is deficient in the repair of *O*^6^-methylguanine residues.

Unexpectedly, the SOS regulated *recN* and *tisAB* genes were found to be significantly upregulated in response to MNNG and these observations were also verified by RT-qPCR (Fig. 4). The induction of these SOS genes in response to MNNG treatment indicates a dual stress response, an effect that has previously been observed in various recombination deficient mutants treated with either MMS or MNNG^44^,^45^. Baek, et al.^32^ also showed that *recN* was significantly upregulated in the wild type strain (K-12 W3110) following 30 minutes of MMS treatment. Furthermore, these authors also found that *recN* was even more strongly induced in their *ada* mutant strain following MMS treatment. In line with these data we demonstrate here upregulation of *recN* following MNNG treatment of an *ada* mutant strain (Fig. 4). This was also shown for *tisAB* in the same *ada* mutant strain (Fig. 4). It thus appears that the SOS genes *recN* and *tisAB* are not regulated by the adaptive response. In the SOS response network, *recN* and *tisAB* expression is tightly regulated by the transcriptional repressor LexA that binds to SOS operator sequences in the promoter regions^46^. Despite *lexA* and *recA* not being significantly modulated by MNNG in the array, *recN* and *tisAB* were not upregulated in the strain DM49, which has a non-cleavable variant of LexA (Table 4). This prevents the induction of the SOS regulon and thus demonstrates that *recN* and *tisAB* are induced in a LexA dependent process. This response distinguishes itself from an UV induced SOS response in that genes such as *umuC* and *uvrB* are upregulated disproportionally less in response to MNNG (Table 4)^33^,^35^. RecN is a conserved SMC-like protein in bacteria. SMC (structural maintenance of chromosomes) proteins are nucleoid-associated ATPases involved in the tethering of chromatids and in double strand break repair^47^,^48^,^49^. Thus, our data suggest that RecN is not only involved in double-strand break repair but also other types of repair such as base lesion repair.

The *tisAB* transcript was shown to express a small peptide, TisB, which localizes to the inner-membrane of the cell^50^. We have previously shown that TisB modulates the regulation of the SOS response and inhibits several SOS response functions^51^. Moreover, it appears that a 20 nucleotide region of complementarity between the *uxaA* gene of the Entner Doudoroff pathway and the 5' untranslated region of *tisAB* could link sugar metabolism to the SOS response^51^. TisB has also been shown to be involved in the production of persister cell formation induced by ciprofloxacin. *In vitro* work has shown that this effect could be produced by forming anion-selective pores in the lipid bilayer^52^,^53^. TisB therefore appears to be a peptide that has an important regulatory function in several stress response networks, including MNNG and UV induced stress.

The flavohemoglobin family is widespread in prokaryotes but their functional annotations are still controversial. Different cellular responses linked to oxidative and/or nitrosative stress have been proposed^54^. The flavohemoglobin of *E. coli*, Hmp, is regulated in response to exogenous signals such as anoxia, redox state, oxidative and nitrosative stress^55^. Hmp expression is regulated by the positive transcription factor FeS protein FNR (fumarate and nitrate reduction) that bind two FNR binding sites upstream of the *hmp* gene^56^. Transcription of *hmp* is not affected by the *ada*-regulon in *E. coli* (Fig. 4). Therefore our data indicate that MNNG upregulates *hmp* in *E. coli* by activating NO related stress response pathways but not by the adaptive response.

Downregulation of prophage related genes (*pinR*, *pinQ* and *ydfK*) as well as the cold-shock related *cspH* were also detected. Interestingly, Baek, et al.^32^ also reported a slight downregulation of *cspH* (fold change −0.44) following MMS exposure, suggesting a general downregulation of this gene in response to alkylating agents.

### Regions assigned as UTRs or part of operon elements

A total of 475 differentially expressed regions were found outside known annotations on either strand, 226 of these regions were assigned roles as UTRs or parts of operons according to the sorting criteria described in Methods. RegulonDB, which is a database that creates models of the complex regulation of transcription initiation or regulatory network of the cell^57^, was used for further investigations of the transcripts believed to be parts of operons. The RegulonDB describes 813 operons with two or more genes, of which 621 operons were transcribed in the reference dataset according to the sliding window method. However, one should keep in mind that the detected transcripts might not only represent UTRs or parts of operons, but could potentially also form other types of transcripts, such as ncRNAs or short peptides.

### Verification of previous *in silico* predicted ncRNAs

In order to verify the novel transcripts, we performed a meta-analysis where we compared the data with previously predicted ncRNAs. Two independent studies were used; 306 predicted ncRNAs from Saetrom, et al.^58^ and 1235 unique ncRNAs compiled by Hershberg, et al.^59^. From these two datasets, a total of 171 ncRNAs *intersect* and a total of 1370 ncRNAs are predicted to be novel in *either* study. Table 5 presents a summary of the predictions compared to our study. Table 6 and Supplementary Table S1 show the 14 differentially expressed regions overlapping with the predicted ncRNAs by Saetrom, et al.^58^, of which seven intersect with ncRNAs listed in the study by Hershberg, et al.^59^. Complete lists of differentially and similarly expressed regions that are found to overlap with previously predicted ncRNAs^58^,^59^ are provided in Supplementary Tables S1–S4.

### Comparison to previous transcriptome studies

The first report of a genome-wide microarray study of *E. coli* was published by Selinger, et al.^26^. In comparison, the study presented here shows a higher detection level of annotated genes. The number of differentially regulated transcripts between log and stationary phase in *E. coli* reported by Selinger, et al.^26^ was slightly higher than the number reported here for reference versus treated bacteria. It is reasonable to believe that the relative changes between log and stationary phase are larger than the changes between treated and untreated bacteria since the repair mechanism should be specific, while the change from stationary to log phase is believed to be more global in an expression context.

A tiling array study by Courcelle, et al.^33^ described transcription in UV-exposed wild-type *E. coli* as compared to non-treated cells. These authors reported an upregulation of known SOS response genes such as *recN*, *recA* and *sulA* in addition to several genes unrelated to the SOS response. Interestingly, Courcelle et al. also reported a large number of genes which were downregulated following UV-irradiation. This finding corresponds to our observation of downregulated transcripts as well as a general decrease of the expression level from reference to MNNG-treated *E. coli*. Interestingly in response to UV, albeit after 15 minutes, a decrease in various nucleoid associated proteins (NAPs), *stpA*, *hupB*, *dps* and *hns* was seen, differing significantly from the MNNG response^35^.

In addition, other *E. coli* transcriptome mapping studies performed by Tjaden et al.^34^,^60^ reported on several possible short RNAs or ORFs, UTRs and operons. In the following comparison with operon and UTR data^34^, only regions adjacent to genes present in the annotation used here were considered. Tjaden et al. used unstressed bacteria and identified 200 genes that were previously known to form parts of operons, as well as predicting 269 novel operon genes. In our study, 187 of the 200 previously known and 247 out of 269 novel operon genes detected by Tjaden, et al.^34^ were identified. This strong overlap between the studies gives confidence to our findings. Additionally, we detected 346 out of 528 genes with novel 5'UTRs and 192 out of 372 genes with novel 3'UTRs predicted in the reference *E. coli*. Furthermore, Tjaden, et al.^60^ presented a list of 340 novel transcripts, suggested to be ORFs or ncRNAs and of these, 60 were identified in our study as novel transcripts with no previous function and 268 of the remaining transcripts were suggested to be UTRs or part of operon elements in our study.

A study by Baek, et al.^32^ reported on transcriptomic and proteomic changes following MMS treatment in the *E. coli* strain K-12 and an *ada* mutant. Although the authors used a different microarray approach, the biological questions are quite similar. The study elegantly outlined the effects MMS has on the stress response pathways, demonstrating upregulation of genes involved in DNA replication, recombination and stationary phase responses. The authors also found that expression of genes belonging to processes such as flagellar biosynthesis, chemotaxis and two-component regulatory systems was controlled by Ada. A comparison of our findings to those of Baek et al. revealed that *ada*, *alkB* and *recN* were the only genes similarly regulated in response to both MNNG and MMS. All three genes were upregulated.

A comprehensive study of *E. coli* transcription by Cho, et al.^61^ presented a total of 2667 transcript segments consisting of 3,183, 501 nt. To further assess the consistency of our transcript detection using custom microarrays, the Cho et al. data was compared to the complete set of expressed nucleotides in our wild type (possible antisense transcripts removed) and then mapped back to the current annotation (NC_000913). NC_000913 annotates a total of 4321 genes (mRNAs, tRNA, rRNAs and ncRNAs), which consists of 3,946,038 nt while our study presents 4,279,881 expressed nucleotides. When mapping these expressed segments back to the Cho et al. data we find that there are 153,767 nt from the Cho et al. study not covered by our study, and 1,240,167 nt defined as expressed in our study are not reported by Cho et al. Further analysis of these expressed nucleotides, which are exclusive to the two studies, show that 86% and 74% of the expressed nucleotides are previously unannotated in the Cho, et al.^61^ and this study, respectively. Comparatively the number of detected transcripts appears relatively high in the present study, but as mentioned in Methods (and discussed by Thomassen et al.^31^,^35^) this is due to the sliding-window algorithm, which is optimized for detection of small differentially expressed regions. Furthermore, this study presents a higher number of expressed nucleotides compared to the Cho et al. study. However, the numbers presented herein are in concordance with the studies presented above. This discrepancy is most probably caused by a slightly lower threshold for transcript detection than applied by Cho et al. The above mentioned results do not change our findings of MNNG modulated transcripts, but imposes some uncertainty with respect to similarly expressed transcripts with an expression level close to the background noise level.

Recent studies by Ragahavan et al.^29^ and Shinhara et al.^28^ have employed deep sequencing (RNA-Seq) to detect novel small RNAs in *E.coli*. Ragahavan et al. identified ten new sRNAs and nine new regulatory leader sequences. Of the ten novel sRNAs, four (*ychE-oppA, yejG-bcr, yigE-corA* and *glnA-typA*) overlapped with similarly expressed transcripts identified in our study, while two (*sdiA-yecC* and *yhcF-yhcG*) overlapped both with similarly and differentially expressed transcripts. Among the nine new regulatory leader sequences, one (*ybjM*) overlapped with a similarly expressed transcript identified in our study, while another one (*mdtJ*) overlapped both with a similarly and a differentially expressed transcript. Shinhara et al. reported 229 novel candidate sRNAs with computational or experimental evidence of transcription initiation. Of these, ten overlapped with differentially expressed transcripts identified in our study, while 38 overlapped with similarly expressed transcripts and five overlapped with both similarly and differentially expressed transcripts, as shown in Supplementary Table 5.

### Promoter search for Ada responsive genes

The study by He, et al.^10^ revealed that the previously published *ada*-box (AAANNAA/AAAGCGCA)^11^,^12^ might not be the correct signal sequence of the adaptive response genes. As an alternative, two promoter boxes were identified; the A box (AAT) and the B box (GCAA), separated by a 6 nt spacer. A computational search was conducted for these A and B boxes with a 5–7 nt spacer in the upstream regions (200 nt) of all detected genes for both the annotated and the detected start sites. Perfect box A and box B sequences with a 6 nt spacer were identified upstream of two upregulated genes; *hmp* (flavohemoglobin) and *iraP* (hypothetical protein) in addition to the known *ada* and *alkA* genes. Interestingly, the same regulatory sequences were found (with a 6 nt spacer) upstream of four downregulated genes (Table 7). These four genes were *fxsA* (inner membrane protein), *spf* (ncRNA), *csrD* (conserved inner membrane protein) and *yiiX* (predicted peptidoglycan peptidase).

To further investigate these findings we repeated the search on all upstream sequences of all similarly expressed genes. The search revealed an almost identical rate of occurrence of this regulatory sequence (AATNNNNNNGCAA) upstream of similarly and differentially expressed genes, indicating that the regulon promoter sequence must act together with other sequence elements to ensure regulatory specificity. There was no statistically significant difference between the number of *ada* regulatory sequences per transcript in regions upstream of detected and annotated start sites for either similarly or differentially expressed genes. Also, there were only minor differences in the distribution of distances between regulatory sequences and the transcriptional start sites, but these were insufficient to explain the different expression levels of genes with the same upstream regulatory sequence.

## Discussion

A known part of the gene repertoire transcriptionally activated after MNNG treatment is the *ada*-regulon, which consists of four genes, *ada*, *alkA*, *alkB* and *aidB* (reviewed by Sedgwick and Lindahl^7^). As a positive quality control of our experimental setup, we found three of these four genes, *ada*, *alkA* and *alkB*, as the top three differentially expressed protein-coding genes using the annotation based analysis approach. Likewise, using the sliding window method, the same three genes were found among the most strongly modulated genes. However, *aidB* was not found to be significantly upregulated in response to 1 μg/ml MNNG, indicating that *aidB* is not or not as sensitive to MNNG as the other genes. When taken in the context of earlier work^36^ it appears that *aidB* is only significantly induced at higher concentrations of MNNG. The lack of *aidB* induction was also verified in additional experiments using RT-qPCR. Also, in a study by Baek, et al.^32^ the authors reported a very weak response of *aidB* after MMS treatment in the wild type *E. coli* strain K-12 W3110, which further supports this conclusion. One possible explanation for this weak or even non-existent response for *aidB* following either MMS or MNNG treatments at these concentrations might be that it has a more appreciable role in survival against a chronic or more extreme acute alkylating challenge.

This work and results from others^35^,^62^,^63^,^64^ indicate that the response to cytotoxic agents is a global process that encompasses both tightly controlled specific stress responses and modulation of the nucleoid structure via NAPs to modify many aspects of cellular metabolism to aid in survival and repair. Various studies^64^ have recently shown that NAPs can modify transcription of hundreds of the transcripts and as such can direct global transcription according to the immediate biological and physical conditions a bacterium may find itself exposed to. These results and those of others^62^,^63^ show that cytotoxic agents can result in the induction of several specific stress response regulons to different extents so as to tailor the transcriptional response to the specific agent. The discrete assignment of genes to particular regulons and their association with a particular type of DNA damage should not necessarily preclude their involvement in the response to other types of agents that challenge the integrity of DNA. An individual chemical or type of radiation will give rise to a variety of biological consequences capable of inducing a range of transcriptional responses. The finding of differential expression of several genes for example *recN* and *tisAB* normally associated with the SOS stress response is therefore understandable in this context where other typical SOS genes such as *umuC* and *uvrB* are not upregulated to the extent as seen in a typical response to UV radiation (Table 4).

Several differentially expressed genes, which have not previously been identified as members of the *ada*-regulon, were found to contain promoter regions with *ada* box A and B with a 6 nt spacer. Remarkably, some of these genes were found to be downregulated by MNNG in the tiling array. Compared to downregulated and similarly expressed genes, it appears that the A and B boxes of upregulated genes are located closer to the transcription initiation start site. However, these findings are not found to be statistically significant. Along with the verified unresponsiveness of *aidB* to the investigated strength of induction, this indicates that the box A and B region might be insufficient to regulate the adaptive response genes alone. This *in silico* identification of perfect A and B *ada* boxes for differentially as well as similarly expressed genes, should be followed by promoter-fusion studies for experimental verification of the Met-Ada binding and the effect this may cause on the expression level of the particular gene. These investigations might also explain the mechanisms underlying the unexpected downregulation of genes that may belong to the *ada*-regulon. We, therefore, conclude that there must be other regulatory elements that invoke the upregulation of the adaptive response genes in addition to the Met-Ada binding. However, this regulatory system remains to be elucidated.

The total number of expressed nucleotides in MNNG treated *E. coli* transcriptome was found to be about 3% lower than in the reference bacteria. In addition, three times the number of upregulated genes were found to be downregulated after MNNG stimulation. Hence, we conclude that we have measured a general downregulation of transcriptional activity due to stress, while we see a much more selective and specific upregulation of known mRNAs as well as other yet uncharacterized transcripts, which are presumably needed, in addition to the adaptive response genes, after MNNG exposure. The study by Selinger, et al.^26^ showed a higher number of differentially regulated transcripts between log and stationary phase, as compared to the differences between reference and MNNG treated bacteria reported in this work. According to the data from the present study combined with previous computer based predictions^58^,^65^,^66^,^67^ there seem to be far more *E. coli* ncRNAs than those few that have been verified to date. A high correlation between our novel transcripts and previous predictions was observed. We therefore conclude that high resolution tiling arrays are useful tools for transcript prediction/verification and can efficiently screen large sets of computationally predicted transcripts before applying low-throughput methods such as RT-qPCR or northern blot analysis.

The impact of small ncRNAs as regulators in transcriptional and translational control has become clearer over the past few years. Our study shows MNNG induced downregulation of 17 annotated ncRNAs. Although no functional studies have been performed on any of these transcripts here, we speculate that they play important roles in the regulation of the cellular response to alkylating stress. However, it cannot be excluded that several of these small RNAs encode short peptides. One of the challenges in future investigations would be to determine the biological significance of these differentially regulated small RNAs in different bacterial stress responses.

In addition to the number of novel short transcripts, we have identified and predicted several novel long transcripts (>200 nt), UTRs and operon elements. We believe these findings to be of biological importance since they were well correlated to previous array data. We have also verified some of the findings using RT-qPCR and northern blot analysis. However, the biological function of these transcripts in the adaptive response network remains unknown.

## Methods

### Strain and growth conditions

*Escherichia coli* K-12 strains MG1655 (F- λ- ilvG- rfb-50 rph-1), AB1157 (F– λ – rac- thi-1 hisG4 Δ(gpt-proA)62 argE3 thr-1 leuB6 kdgK51 rfbD1 *araC14 lacY1 galK2 xylA5 mtl-1 tsx-33 supE44(glnV44) rpsL31(strR)*) or DM49 (AB1157 *lexA3*) were used in this study. Overnight cultures were diluted 1:500 in K-medium [39] (1 × M9, 1.2% glucose, 1.25% casamino acids (dCAA), 1 mM MgSO_4_, 0.1 mM CaCl_2_) and subsequently grown at 37°C. Cells were grown in 100 ml batch cultures in 500 ml Erlenmeyer flasks with aeration by rotary shaking. At OD_600_ = 0.5 the adaptive response was induced in 50 ml of the cultures by adding 1 μg/ml MNNG^68^. The MNNG was dissolved in DMSO. Cells were harvested by centrifugation 60 minutes after MNNG exposure. The reference bacteria (with DMSO) were extracted from the culture before the addition of MNNG.

### RNA isolation, cDNA synthesis and target labelling

RNA was isolated as described by Saetrom, et al.^58^. The RNA was reverse transcribed to cDNA, fragmented and labelled using the Affymetrix ‘Prokaryotic Sample and Array Processing protocol version 701029 Rev. 4. Five replicates were run for each of the two conditions.

### Reverse transcriptase Real-Time quantitative polymerase chain reaction (RT-qPCR)

cDNA was generated from the DNaseI treated total RNA solutions using a volume equivalent to 1 μg RNA and using the High Capacity cDNA Reverse Transcription Kit (ABI). The reactions were carried out according to the manufacturer's instructions. The Power SYBR Green PCR MasterMix was used in conjunction with the StepOnePlus Real-Time PCR System (ABI) and the equivalent of 5 ng of the cDNA to generate the Real-Time plots to be processed by the associated software. StepOne Software v2.0.1 was used in order to generate the cycle threshold (Ct) values. The primers for the RT-qPCR regarding the candidate transcripts were designed using Primer Express 3.0 (ABI). Primer sequences are found in Supplementary Tables S6–S8. The samples were run in quadruplets. In addition, three independent parallels were run. The Ct value was then subtracted from that of *rrsB* (16S ribosomal RNA), a stably expressed gene, in order to give a value specific to the transcript. The *rrsB* gene did not belong to the group of transcripts detected as differentially expressed in the tiling array data and could therefore be included as a standard gene for the RT-qPCR validation study (probability of differential expression ~ 0). The specificity of the PCR reactions was determined from dissociation curves generated after the RT-qPCR reactions. The visual representation of the transcript specific values showing fold change, assumes a doubling of PCR product each PCR cycle.

### Array design and data processing

The genome sequence of *E. coli* K12 MG1655 was downloaded together with the current annotation from the NCBI ftp-site (May 24^th^, 2005). From this annotation the genome was split in two; 1) protein-coding transcripts including tRNAs and rRNAs and 2) non-coding transcripts. Due to the limitation regarding the number of array probes, a selective tiling approach was taken in order to ensure high coverage of the unannotated genomic regions. The coverage was 19 probes per coding region with an average intergenic resolution of 14 nt as well as a maximum resolution of 7 nt for the intergenic regions. The 17 micron 282,000 feature NimbleExpress arrays were used and are further described by Thomassen, et al.^31^.

The arrays were scanned using GeneChip Scanner 3000 7 G. The array data were processed according to the methods described in Thomassen, et al.^31^. The minimum signal intensity of a probe signal was set to 9.0 on a log_2_ scale. All measured intensities below 9.0 were considered uncertain as these probe values were inseparable from background noise. A short outline of the post-processing procedures i.e. the annotation based approach, the sliding window method and the transcriptome mapping are described below. The microarray data are accessible through the NCBI Gene Expression Omnibus (http://www.ncbi.nlm.nih.gov/geo/); GPL 7714 (array) and GSE 13830 (data). Also, GSE 13830 contains Supplementary Information Files and Tables regarding all differentially and similarly expressed transcripts not presented herein.

The *E. coli* genome annotation used in the post-processing annotated 4321 transcripts, including tRNAs, rRNAs, and ncRNAs, of which the 4200 protein-coding genes and all the ncRNAs were targeted by one or more probes. The annotation (NC_000913) was downloaded from the NCBI ftp-site December 13th, 2007. The ncRNA gene *istR* was split into *istR1* and *istR2* according to Vogel, et al.^69^, hence making a total of 63 annotated ncRNA genes.

### Data analysis

Three different analysis methods were used in this study. The first method was an annotation-based method only considering previously annotated transcripts. The probability of an annotated gene being differently expressed was computed using a t-test comparing the probe intensity values for all probes targeting the given gene in the reference and the treated sample. The second approach was a *novel*, annotation *independent* method (referred to henceforth as the sliding window method), which applied dynamic programming with a t-test based data segmentation algorithm that simultaneously performed a reference versus treated comparison. The algorithm computed both a probability score (P) for each transcript that were differentially expressed as well as a log_2_ fold change value. Finally, the third method detected transcripts found to be either absent or present i.e. either below (<9 log_2_ signal) or above (≥9 log_2_ signal) background in the tiling array without any prior comparison or knowledge of the data. The minimum length of a differentially expressed region was set to 25 nt while a constantly expressed transcript needed to exceed 35 nt. All differentially expressed genes and transcripts had a probability score of at least P > 0.95 and a fold change of > 0.5 (log_2_). All of these methods are presented in detail by Thomassen, et al.^31^.

All detected transcripts were subsequently mapped to the annotation. All regions overlapping an annotated transcript with one or more nucleotides are herein described as “touching” a transcript. Also, all regions with one end located < 100 nt up- or downstream of a gene were assigned as potential 5′ or 3′ UTRs. Likewise, regions located < 100 nt of both an upstream and a downstream gene were suggested as part of a potential operon element. This 100 nt distance cut-off is approximately 20% higher than the average UTR length suggested by Bernstein, et al.^70^ and should therefore include most UTRs. All remaining regions were considered as unannotated transcripts.

## Supplementary Material

Supplementary Information Supplementary Information

## Figures and Tables

**Table 1:** Classification of differentially expressed transcripts detected using the sliding window method^31^

	Differentially expressed		Similarly expressed	
Classification	Genes*	Regions	Genes*	Regions
Annotated on same strand	241 (17)	322	4176 (56)	4506
Potential operon elements**	-	68	-	1428
Potential 5'UTRs**	-	168	-	1954
Potential 3'UTRs**	-	114	-	1838
Potential novel transcripts with no previous function	-	250	-	2275

*Numbers in parenthesis indicate the number of transcripts annotated as ncRNAs.

**The number of UTRs and operon elements consisted of the sum of lone-standing transcripts assigned a role as a UTR or part of an operon element as well as parts of transcripts that spanned more than one protein-coding gene.

**Table 2:** The 15 most differentially expressed unannotated transcripts following MNNG exposure

ID	Strand	Start	End	Length	Fold change (log2)
novel1	−	1638816	1638983	168	−2.68
novel2	+	1635853	1635913	61	−2.12
novel3	−	1905921	1905998	78	−1.84
novel4	+	4390901	4390941	41	−1.67
novel5	+	4390794	4390841	48	−1.66
novel6	+	3646047	3646133	87	−1.60
novel7	−	3655638	3655792	155	−1.58
novel8	+	2494751	2494903	153	−1.57
novel9	+	1431799	1431929	131	−1.55
novel10	+	17175	17235	61	−1.53
novel11	+	4324950	4325013	64	−1.52
novel12	+	1630469	1630556	88	−1.52
novel13	+	3655786	3655834	49	−1.46
novel14	−	2403446	2403519	74	−1.44
novel15	−	837261	837295	35	−1.43

**Table 3:** Summary of the number of transcripts found to be expressed in MNNG treated and reference bacteria, respectively, using the sliding window approach

	Genes		Regions	
	Reference*	Treated*	Reference	Treated
Genes (ncRNAs)	4220 (61)	4184 (59)	3339	3654
Novel transcripts	-	-	2350	2521

*The numbers in parenthesis indicate the number of ncRNAs.

**Table 4:** Expression fold change (log2) of selected genes after exposure to MNNG or UV in four experiments. The *E. coli* strains AB1157 and DM49 (non-cleavable LexA repressor) were exposed to MNNG and the resulting changes examined after 1 h. The results of Courcelle, et al.^33^ and Thomassen, et al.^35^ are in response to UV, 1 hour and 15 minutes after treatment, respectively. The base 2 logarithms of the expression fold change ratios are shown

	AB1157		DM49			
Gene	Mean	StDev	Mean	StDev	Courcelle	Thomassen
*aidB*	−0.14	0.10	1.14	0.08		−0.07
*recN*	2.86	0.10	0.32	0.09	4.53	3.18
*recA*	1.87	0.07	1.08	0.04	2.19	2.08
*tisAB*	2.22	0.06	0.31	0.25		2.46
*uvrB*	0.55	0.15	0.33	0.07	2.21	1.70
*umuC*	1.75	0.06	−0.45	0.21	4.51	2.89
*sulA*	1.93	0.07	−1.25	0.07	3.23	2.82

**Table 5:** Overview of transcripts detected by the sliding window method found to overlap with ncRNA predictions of previous studies. Intersection denotes the numbers of unique candidates present in both studies from Saetrom, et al.^58^ and Hershberg, et al.^59^. Union denotes the number of unique candidates found to be present in the two studies in total. The intersection and union results should be considered in combination with the data presented in the two first data rows

Study	Predicted (total)	Present in reference cells	Present in treated cells	Similarly expressed	Differentially expressed	Present neither in reference nor treated cells
Saetrom, et al.^58^	306	269	265	255	14	35
Hershberg, et al.^59^	1235	1176	1150	1114	14	55
Intersection	171	149	148	143	7	1
Union	1370	1296	1267	1226	21	69

**Table 6:** Differentially expressed regions, detected by the sliding window method, which overlapped with predicted ncRNAs from previous studies

ID	Saetrom, et al.^58^	Reference	Overlap with Saetrom, et al.^58^	Region length	Left gene	Right Gene	Fold-change (log_2_)	RT-qPCR (Fig. 3)
R1	I253	Tjaden, et al.^60^	100%	92	*yjhX*	*yjhS*	−1.0	nc4
R2	I179	Chen, et al.^66^	54%	103	*yjhX*	*yjhS*	−1.0	nc4
R3	I176	-	100%	65	*insH*	*insD*	−1.0	
R4	I287	-	24%	47	*insH*	*insD*	−0.9	
R5	I202	Carter, et al.^65^	50%	91	*yfjW*	*yfjX*	−0.9	nc3
R6*	I073	-	40%	33	*yfhL*	*ryfB*	−0.9	
R7	I006	-	11%	25	*thrW*	*ykfI*	−0.9	
R8	I248	Chen, et al.^66^	100%	145	*insA*	*rpsT*	−0.9	nc10
R9#	I159	Tjaden, et al.^60^	44%	25	*dinQ*	*dinQ*	−0.9	
R10	I291	-	100%	86	*torY*	*cutC*	−0.8	
R11	I074	Rivas, et al.^67^	12%	32	*uof*	*fldA*	−0.8	
R12	I165	-	42%	73	*djlA*	*rluA*	−0.7	nc6
R13	I238	Carter, et al.^65^	2%	92	*dinQ*	*arsR*	+0.6	
R14	I032	-	32%	43	*yefM*	*hisL*	−0.6	

*candidate overlapping with the experimentally verified *ryfB* (2698081,2698399).

#candidate found to be located on the opposite strand of *dinQ* (3645728,3645856).

**Table 7:** Result of the promoter search 200 nt upstream of the annotated start site for the genes detected as differentially expressed by the sliding window method. Box A (AAT) and box B (GCAA) are shown in bold

Upstream distance	Spacer	Sequence	Gene	Start site	Fold change (log_2_)	Probability (differentially regulated)	Strand
81	6	**AAT**GGAAAA**GCAA**	*csrD*	3401354	−0.85	1.00	−
178	6	**AAT**ACTAAA**GCAA**	*yiiX*	4125917	−0.73	1.00	−
101	6	**AAT**AGTTGC**GCAA**	*fxsA*	4366687	−1.57	1.00	+
64	6	**AAT**TCCTAT**GCAA**	*spf*	4047922	−1.51	0.99	+
16	6	**AAT**TAAGAT**GCAA**	*hmp*	2683857	+1.09	1.00	+
97	6	**AAT**ATTTGC**GCAA**	*iraP*	400610	+0.67	0.97	+
66	6	**AAT**TAAAGC**GCAA**	*ada*	2308427	+1.76	1.00	−
53	6	**AAT**ATGAAA**GCAA**	*alkA*	2145564	+2.72	1.00	−
