# Supplementary material for: Tiling array study of MNNG treated Escherichia coli reveals a widespread transcriptional response
Source: Sci Rep. 2013 Oct 25;3:3053. doi: 10.1038/srep03053 (PMC6505713; doi:10.1038/srep03053)
Supplement: Supplementary Information [file srep03053-s1.pdf]

Tiling array study of MNNG treated *Escherichia coli* reveals a widespread transcriptional response

James A. Booth, Gard O. S. Thomassen, Alexander D. Rowe, Ragnhild Weel-Sneve, Karin Lagesen, Knut I. Kristiansen, Magnar Bjørås, Torbjørn Rognes and Jessica M. Lindvall

Supplementary Information

Contents

Supplementary Table S1 – Page 2

Supplementary Table S2 – Page 3

Supplementary Table S3 – Page 10

Supplementary Table S4 – Page 12

Supplementary Table S5 – Page 38

Supplementary Table S6 – Page 40

Supplementary Table S7 – Page 41

Supplementary Table S8 – Page 42

Supplementary Table S1

Differentially expressed transcripts overlapping with ncRNA predicted by Saetrom *et al.* (2005)

Transcripts were detected using the sliding window method. All Saetrom predicted ncRNAs that overlap with differentially expressed regions in this study. Info on Saetrom data is found in columns 1-6. Info on candidates also found in the list by Hershberg *et al.* (2003) and others is found in column 7. Info on the differentially expressed regions that overlap Saetrom predicted ncRNAs from this study is found in column 8-17. Signal intensity values and fold change are $\log_2$ values.

Saetrom ID	Saetrom start	Saetrom end	Saetrom length	Saetrom strand	Saetrom info	Prev pred.	Fold change	Length	Start	End	Strand	P(diff reg)	Regulation	Reference signal	Treated signal	RT-qPCR ID
I253	4532252	4532302	50	+	0.013 (0-50)	4532142-4532310 + Tjaden et al.	-1.05	92	4532242	4532334	+	1.00	DOWN	12.17	11.12	nc4
I179	4532433	4532483	50	+	0.032 (0-50)	4532323-4532459 - Chen et al.	-0.99	103	4532456	4532559	+	1.00	DOWN	11.22	10.22	nc4
I176	2066068	2066118	50	+	0.033 (0-50)	~	-0.98	65	2066057	2066122	+	1.00	DOWN	11.97	10.99	
I287	2065455	2065505	50	-	0.0035 (0-50)	~	-0.94	47	2065493	2065540	-	1.00	DOWN	11.02	10.08	
I202	2773242	2773342	100	+	0.026 (0-50), 0.026 (25-75), 0.022 (50-100)	2773203-2773322 + Carter et al.	-0.93	91	2773201	2773292	+	1.00	DOWN	11.89	10.96	nc3
I073	2698088	2698138	50	-	0.078 0.054 (0-50)	~	-0.93	33	2698075	2698108	-	1.00	DOWN	10.62	9.69	
I006	262270	262352	82	-	0.079 (0-50), 0.18 0.14 (7-57), 0.0062 (32-82)	~	-0.92	25	262343	262368	-	0.99	DOWN	11.24	10.32	
I248	20665	20715	50	-	0.015 (0-50)	20666-20798 + Chen et al.	-0.91	145	20629	20774	-	1.00	DOWN	12.18	11.27	
I159	3645831	3645881	50	+	0.039 (0-50)	3645700-3645859 + Tjaden et al.	-0.90	25	3645828	3645853	+	1.00	DOWN	10.26	9.36	
I291	1956394	1956444	50	-	0.0029 (0-50)	~	-0.81	86	1956391	1956477	-	1.00	DOWN	10.92	10.11	
I074	710008	710058	50	-	0.077 (0-50)	709961-710159 - + Rivas et al.	-0.80	32	709982	710014	-	1.00	DOWN	13.99	13.19	
I165	59529	59579	50	+	0.038 (0-50)	~	-0.73	73	59477	59550	+	1.00	DOWN	11.85	11.12	nc6
I238	3645983	3646033	50	-	0.018 (0-50)	3645948-3646027 - Carter et al.	0.64	92	3645892	3645984	-	1.00	UP	13.57	14.21	
I032	2087862	2087912	50	-	0.11 (0-50)	~	-0.59	43	2087896	2087939	-	1.00	DOWN	10.53	9.94	

Supplementary Table S2

Similarly expressed transcripts that overlapped with the ncRNAs predicted by Saetrom *et al.* (2005)

Transcripts detected by the sliding window method. All Saetrom predicted ncRNAs that overlap with similarly expressed regions in this study. Info on Saetrom data is found in columns 1-6. Info on candidates also found in the list by Hershberg *et al.* (2003) and others is found in column 7. Info on the similarly expressed regions that overlap Saetrom predicted ncRNAs from this study is found in columns 8-14. Signal intensity values are log_2 values.

Saetrom ID	Saetrom start	Saetrom end	Saetrom length	Saetrom strand	Saetrom info	Prev pred.	Start	End	Length	Strand	Reference signal	Treated signal	P(Diff reg)
I108	4188163	4188213	50	-	0.061 (0-50)	4188185-4188292 + Tjaden et al.	4187937	4188532	595	-	12.02	11.7	0.003
I109	3182582	3182671	89	-	0.011 (0-50), 0.058 (14-64), 0.06 0.04 (39-89)	3182546-3182665 - Carter et al.	3181731	3182750	1019	-	12.54	12.4	0.001
I170	1735718	1735768	50	-	0.036 (0-50)	~	1735663	1735720	57	-	11.12	10.46	0.016
I170	1735718	1735768	50	-	0.036 (0-50)	~	1735726	1735785	59	-	10.46	10.38	0.002
I271	1766809	1766859	50	-	0.0075 (0-50)	1766800-1766919 + Carter et al.	1763235	1766952	3717	-	11.23	11.19	0.000
I111	914325	914375	50	-	0.058 (0-50)	914218-914571 - + Rivas et al., 914259-914378 + Carter et al.	914238	914492	254	-	9.67	9.76	0.004
I273	3769758	3769808	50	-	0.0069 (0-50)	~	3769434	3769811	377	-	10.08	9.69	0.007
I274	1642517	1642567	50	+	0.0068 (0-50)	1642458-1642537 + Carter et al.	1642488	1642584	96	+	9.9	9.86	0.001
I089	3723266	3723316	50	-	0.069 (0-50)	3723150-3723337 - Tjaden et al., 3723226-3723305 - Carter et al., 3723282-3723377 + Tjaden et al.	3720538	3723442	2904	-	11.38	11.35	0.000
I088	2474298	2474348	50	+	0.07 (0-50)	~	2474242	2474405	163	+	10.11	9.79	0.005
I087	1218274	1218324	50	+	0.07 0.051 (0-50)	~	1218277	1218431	154	+	11.13	10.99	0.002
I085	2773665	2773840	175	-	0.0011 (0-50), 0.07 0.013 (25-75), 0.021 (75-125), 0.056 0.011 (125-175)	2773603-2773682 + Carter et al., 2773723-2773802 + Carter et al., 2773770-2773849 - Carter et al.	2773595	2773680	85	-	9.58	9.22	0.009
I085	2773665	2773840	175	-	0.0011 (0-50), 0.07 0.013 (25-75), 0.021 (75-125), 0.056 0.011 (125-175)	2773603-2773682 + Carter et al., 2773723-2773802 + Carter et al., 2773770-2773849 - Carter et al.	2773684	2773904	220	-	9.8	9.61	0.003
I084	2302953	2303003	50	-	0.071 (0-50)	2302928-2303047 - Carter et al.	2302646	2303930	1284	-	10.39	10.25	0.002
I083	1355234	1355284	50	-	0.072 (0-50)	~	1355176	1356810	1634	-	11	10.69	0.002
I082	3645508	3645583	75	-	0.016 (0-50), 0.072 (25-75)	3645316-3645610 - Tjaden et al.	3644506	3645672	1166	-	9.79	9.49	0.005
I081	576446	576521	75	-	0.072 0.038 (0-50), 0.033 (25-75)	576300-576615 + Tjaden et al., 576356-576507 + Chen et al.	576422	576509	87	-	10.73	10.82	0.002
I080	3850990	3851065	75	+	0.00057 (0-50), 0.073 0.069 (25-75)	~	3849695	3851037	1342	+	9.73	9.63	0.001
I007	4626216	4626291	75	+	0.17 (0-50), 0.11 0.0052 (25-75)	~	4622883	4627703	4820	+	10.53	10.5	0.000
I006	262270	262352	82	-	0.079 (0-50), 0.18 0.14 (7-57), 0.0062 (32-82)	~	262222	262352	130	-	10.27	9.56	0.013
I005	303544	303594	50	-	0.19 (0-50)	~	303548	303596	48	-	9.73	9.57	0.005
I004	3766615	3766665	50	+	0.21 0.02 (0-50)	~	3766618	3766696	78	+	9.8	9.73	0.002
I003	719883	719958	75	+	0.21 0.086 (0-50), 0.095 (25-75)	719854-719973 + Carter et al.	719890	719931	41	+	9.32	9.32	0.000
I002	4230937	4231087	150	-	0.13 0.038 (0-50), 0.011 (25-75), 0.22 (75-125), 0.11 0.058 (100-150)	4230927-4231086 - Carter et al.	4229870	4231553	1683	-	10.67	10.62	0.000
I001	271879	271979	100	+	0.22 (0-50), 0.03 (25-75), 0.016 (50-100)	271880-272035 + Carter et al.	271841	271883	42	+	9.76	9.79	0.001
I001	271879	271979	100	+	0.22 (0-50), 0.03 (25-75), 0.016 (50-100)	271880-272035 + Carter et al.	271889	272447	558	+	10.36	10.27	0.001
I178	1766809	1766859	50	+	0.032 (0-50)	1766800-1766919 + Carter et al.	1766770	1768304	1534	+	10.57	10.34	0.002
I176	2066068	2066118	50	+	0.033 (0-50)	~	2066027	2066071	44	+	12.15	10.82	0.043
I176	2066068	2066118	50	+	0.033 (0-50)	~	2066107	2066169	62	+	10.99	10.34	0.017
I175	1268767	1268817	50	+	0.033 (0-50)	~	1262830	1269867	7037	+	11.69	11.38	0.002
I174	1269397	1269447	50	-	0.033 (0-50)	1269412-1269531 - Carter et al.	1268279	1269894	1615	-	10.22	10.03	0.007

I172	2640964	2641014	50	+	0.034 (0-50)	~	2640958	2641014	56	+	10.1	9.94	0.004
I009	1859481	1859606	125	+	0.16 (0-50), 0.048 (50-100), 0.11 (75-125)	1859567-1859646 + Carter et al.	1859478	1859571	93	+	10.43	10.37	0.001
I009	1859481	1859606	125	+	0.16 (0-50), 0.048 (50-100), 0.11 (75-125)	1859567-1859646 + Carter et al.	1859586	1859689	103	+	10.12	9.97	0.003
I008	1702671	1702746	75	+	0.13 0.023 (0-50), 0.16 0.047 (25-75)	1702604-1702818 + Tjaden et al.	1702438	1702914	476	+	12.17	11.54	0.005
I110	3851115	3851165	50	+	0.059 (0-50)	3851106-3851185 + Carter et al., 3851123-3851297 + Tjaden et al.	3851128	3851207	79	+	9.84	9.79	0.002
I100	4054055	4054105	50	-	0.067 (0-50)	4054045-4054204 - Carter et al.	4051444	4054400	2956	-	11.28	11.19	0.001
I101	4076394	4076444	50	-	0.067 (0-50)	~	4076005	4076495	490	-	9.52	9.47	0.002
I255	4177374	4177424	50	-	0.013 (0-50)	4177248-4177547 - Tjaden et al., 4177322-4177441 + Carter et al., 4177364-4177443 - Carter et al.	4176522	4178018	1496	-	10.27	10.06	0.001
I279	4529349	4529399	50	+	0.0062 (0-50)	~	4528875	4529748	873	+	9.8	9.51	0.005
I187	1501456	1501506	50	-	0.031 (0-50)	~	1501469	1501514	45	-	11.5	11.52	0.001
I148	3571258	3571308	50	-	0.044 (0-50)	3571143-3571363 + Tjaden et al., 3571208-3571287 - Carter et al., 3571214-3571405 + Chen et al.	3562046	3573072	11026	-	11.45	11.28	0.001
I168	2085190	2085240	50	+	0.037 (0-50)	~	2085180	2085271	91	+	10.37	10.16	0.005
I262	1913329	1913379	50	+	0.011 (0-50)	1913291-1913450 + Tjaden et al.	1911318	1913394	2076	+	10.37	10.26	0.001
I261	2301642	2301692	50	+	0.012 (0-50)	~	2301640	2301703	63	+	10.8	10.85	0.001
I266	279399	279449	50	+	0.0084 (0-50)	279369-279448 - Carter et al., 279430-279509 + Carter et al.	278060	279943	1883	+	10.11	10.03	0.001
I265	1501324	1501374	50	+	0.0086 (0-50)	~	1501169	1501336	167	+	10.37	10.22	0.002
I265	1501324	1501374	50	+	0.0086 (0-50)	~	1501337	1501408	71	+	10.07	9.39	0.019
I264	1620759	1620834	75	-	0.0089 (0-50), 0.0056 (25-75)	1620744-1620823 + Carter et al.	1620772	1620811	39	-	9.67	9.68	0.000
I264	1620759	1620834	75	-	0.0089 (0-50), 0.0056 (25-75)	1620744-1620823 + Carter et al.	1620831	1620944	113	-	13.14	13.11	0.000
I269	4055789	4055839	50	+	0.008 (0-50)	4055641-4055988 - + Rivas et al., 4055836-4055961 + Tjaden et al.	4054942	4056164	1222	+	10.09	9.85	0.003
I268	3237434	3237484	50	-	0.0082 (0-50)	~	3236826	3237772	946	-	9.7	9.6	0.001
I032	2087862	2087912	50	-	0.11 (0-50)	~	2087812	2087906	94	-	10.34	10.29	0.001
I033	2640964	2641049	85	-	0.11 0.051 (0-50), 0.012 (35-85)	~	2633112	2641130	8018	-	11.78	11.52	0.001
I031	1195940	1195990	50	+	0.11 (0-50)	~	1195941	1195977	36	+	11.26	11.29	0.001
I036	1306819	1306894	75	+	0.012 (0-50), 0.1 0.026 (25-75)	1306800-1306879 + Carter et al.	1306726	1306978	252	+	12.92	12.48	0.004
I037	1739271	1739321	50	+	0.1 (0-50)	1739222-1739379 + Chen et al., 1739267-1739346 - Carter et al.	1739251	1740912	1661	+	12.5	12.29	0.001
I034	3949641	3949724	83	+	0.0045 (0-50), 0.11 0.031 (33-83)	~	3949586	3950141	555	+	10.27	10.21	0.000
I035	914278	914378	100	+	0.1 (0-50), 0.092 (50-100)	914218-914571 - + Rivas et al., 914259-914378 + Carter et al.	914223	914521	298	+	10.64	10.31	0.008
I142	4531552	4531602	50	+	0.046 (0-50)	4531359-4531856 - + Rivas et al., 4531472-4531746 + Chen et al., 4531483-4531562 + Carter et al., 4531556-4531866 - + Rivas et al.	4531299	4531876	577	+	10.77	10.57	0.003
I143	1080170	1080220	50	-	0.046 0.038 (0-50)	1080042-1080192 - + Rivas et al., 1080160-1080239 - Carter et al., 1080196-1080293 - Tjaden et al.	1080120	1080506	386	-	11	10.74	0.002
I038	4609745	4609795	50	+	0.1 (0-50)	4609572-4609981 - + Rivas et al.	4609267	4610142	875	+	10.83	10.58	0.002
I146	3237309	3237359	50	-	0.045 (0-50)	3237214-3237373 - Carter et al., 3237308-3237387 + Carter et al.	3236826	3237772	946	-	9.7	9.6	0.001
I147	3645733	3645808	75	-	0.045 (0-50), 0.012 (25-75)	3645700-3645859 + Tjaden et al.	3645701	3645860	159	-	11.93	11.04	0.012
I144	89284	89334	50	-	0.046 (0-50)	~	89269	89320	51	-	11.13	10.82	0.010
I208	2202031	2202081	50	-	0.026 (0-50)	~	2202026	2202071	45	-	9.41	9.32	0.004
I232	4531752	4531802	50	+	0.019 (0-50)	4531359-4531856 - + Rivas et al.	4531299	4531876	577	+	10.77	10.57	0.003
I305	3697997	3698047	50	+	0.00056 (0-50)	~	3694818	3698442	3624	+	11	10.74	0.001
I140	1064640	1064690	50	+	0.047 (0-50)	~	1064575	1064672	97	+	10.52	10.24	0.005
I245	2559088	2559138	50	-	0.017 (0-50)	~	2559084	2559131	47	-	9.82	9.88	0.002
I141	2773567	2773667	100	+	0.021 (0-50), 0.046 (50-100)	2773603-2773682 + Carter et al.	2773613	2773660	47	+	9.56	9.59	0.001
I276	3494742	3494792	50	-	0.0064 (0-50)	3494742-3494814 - Tjaden et al.	3494565	3494830	265	-	9.97	9.93	0.002
I253	4532252	4532302	50	+	0.013 (0-50)	4532142-4532310 + Tjaden et al.	4532054	4532253	199	+	11.42	10.93	0.005

I300	4531733	4531783	50	-	0.0012 (0-50)	4531359-4531856 - + Rivas et al.,<br>4531472-4531746 + Chen et al.	4531263	4531764	501	-	9.87	9.78	0.002
I277	271786	271836	50	-	0.0063 (0-50)	271640-271799 + Carter et al.	271775	271837	62	-	9.65	9.72	0.002
I301	606881	606931	50	+	0.0011 (0-50)	606897-607016 + Carter et al.	606878	607029	151	+	10.45	10.17	0.004
I155	4006626	4006676	50	+	0.042 (0-50)	~	4006663	4007112	449	+	10.22	9.66	0.009
I154	311060	311110	50	+	0.042 (0-50)	~	311062	311156	94	+	9.97	9.8	0.004
I157	4465020	4465070	50	+	0.041 (0-50)	4464974-4465173 + Tjaden et al.,<br>4464989-4465068 - Carter et al.,<br>4465014-4465199 - + Rivas et al.,<br>4465069-4465148 - Carter et al.	4464995	4465307	312	+	9.7	9.66	0.001
I156	2190916	2190979	63	+	0.0086 (0-50), 0.041 (13-63)	2190859-2190978 - Chen et al.	2190781	2190976	195	+	9.68	9.46	0.003
I151	4532377	4532427	50	+	0.043 (0-50)	4532323-4532402 + Carter et al.,<br>4532323-4532459 - Chen et al.	4532317	4532408	91	+	11.81	11.22	0.011
I150	2876341	2876391	50	+	0.043 (0-50)	2876232-2876391 - Carter et al.,<br>2876310-2876479 - Chen et al.,<br>2876322-2876401 + Carter et al.	2876227	2876496	269	+	9.98	9.74	0.003
I152	2773415	2773465	50	-	0.043 (0-50)	2773402-2773509 - Tjaden et al.	2773368	2773543	175	-	11.33	11.35	0.000
I256	344348	344398	50	-	0.013 (0-50)	~	344301	344357	56	-	9.39	9.31	0.002
I254	579532	579582	50	-	0.013 (0-50)	579526-579667 + Chen et al.	579498	579545	47	-	9.8	9.95	0.005
I169	2099565	2099615	50	+	0.037 (0-50)	~	2099499	2099706	207	+	9.99	9.88	0.001
I250	3326454	3326504	50	-	0.014 (0-50)	3326392-3326473 - Carter et al.	3324973	3327254	2281	-	12.09	11.88	0.001
I251	573760	573810	50	-	0.014 (0-50)	~	573744	575023	1279	-	9.65	9.7	0.001
I029	2531634	2531684	50	-	0.11 (0-50)	2531669-2531767 - Tjaden et al.	2531624	2534126	2502	-	10.56	10.4	0.001
I028	4517914	4517964	50	-	0.12 (0-50)	~	4517274	4518425	1151	-	11.01	10.86	0.001
I024	3992147	3992222	75	-	0.037 (0-50), 0.12 0.034 (25-75)	3992162-3992241 - Carter et al.	3989072	3992460	3388	-	10.57	10.45	0.001
I027	925791	925841	50	+	0.12 (0-50)	~	925688	925896	208	+	10.57	10.29	0.003
I026	557064	557139	75	+	0.12 0.011 (0-50), 0.021 (25-75)	~	557051	557117	66	+	10.18	10.1	0.002
I021	2751652	2751715	63	+	0.0074 (0-50), 0.12 (13-63)	2751490-2751789 + Tjaden et al.,<br>2751645-2751764 - Carter et al.	2751512	2752043	531	+	12.86	12.67	0.001
I023	1642475	1642525	50	-	0.12 (0-50)	1642458-1642537 + Carter et al.	1642429	1642502	73	-	10.15	9.95	0.005
I023	1642475	1642525	50	-	0.12 (0-50)	1642458-1642537 + Carter et al.	1642516	1642666	150	-	10.46	10.23	0.003
I022	2902296	2902346	50	+	0.12 (0-50)	2902277-2902356 + Carter et al.	2902142	2902440	298	+	9.88	9.61	0.003
I121	2404961	2405011	50	+	0.054 0.0041 (0-50)	2404931-2405010 - Carter et al.	2404985	2405023	38	+	10.55	10.52	0.002
I122	581906	581956	50	-	0.054 (0-50)	~	581698	581985	287	-	9.95	9.57	0.008
I123	2781328	2781403	75	+	0.018 (0-50), 0.054 (25-75)	~	2781268	2781334	66	+	10.33	10.2	0.006
I124	2773742	2773792	50	+	0.053 (0-50)	2773723-2773802 + Carter et al.,<br>2773770-2773849 - Carter et al.	2773677	2773768	91	+	10.58	10.53	0.002
I126	2166559	2166609	50	-	0.053 (0-50)	~	2166568	2166713	145	-	9.36	9.38	0.000
I127	2302978	2303028	50	+	0.052 (0-50)	2302928-2303047 - Carter et al.	2301879	2303060	1181	+	10.97	10.97	0.000
I129	4160999	4161049	50	+	0.052 (0-50)	4160940-4161059 + Carter et al.	4159063	4161360	2297	+	11.05	10.85	0.002
I243	400247	400297	50	+	0.017 (0-50)	~	400241	400286	45	+	9.89	9.84	0.002
I242	4499421	4499471	50	-	0.017 (0-50)	4499430-4499669 + Tjaden et al.	4499302	4499457	155	-	9.56	9.67	0.003
I241	4465024	4465074	50	-	0.017 (0-50)	4464974-4465173 + Tjaden et al.,<br>4464989-4465068 - Carter et al.,<br>4465014-4465199 - + Rivas et al.,<br>4465069-4465148 - Carter et al.	4464681	4465384	703	-	9.95	9.67	0.004
I244	1108339	1108389	50	+	0.017 (0-50)	~	1108297	1108374	77	+	9.73	9.55	0.004
I244	1108339	1108389	50	+	0.017 (0-50)	~	1108386	1112773	4387	+	11	10.63	0.003
I247	2723939	2723989	50	+	0.015 (0-50)	2723788-2724007 + Tjaden et al.,<br>2723937-2724016 + Carter et al.	2723859	2724022	163	+	11.75	11.09	0.008
I246	2765476	2765526	50	-	0.016 (0-50)	2765435-2765514 - Carter et al.,<br>2765455-2765720 + Chen et al.	2764035	2765595	1560	-	10.56	10.26	0.002
I058	1702673	1702723	50	-	0.088 (0-50)	1702604-1702818 + Tjaden et al.	1702666	1702814	148	-	9.73	9.33	0.008
I059	3998813	3998863	50	-	0.088 0.055 (0-50)	3998739-3999039 - + Rivas et al.,<br>3998801-3998946 + Chen et al.,<br>3998828-3998907 - Carter et al.	3998719	3999031	312	-	10.12	9.99	0.001
I050	4499271	4499346	75	-	0.011 (0-50), 0.093 0.041 (25-75)	4499158-4499277 + Carter et al.,<br>4499271-4499390 - Carter et al.	4499302	4499457	155	-	9.56	9.67	0.003
I051	17314	17364	50	-	0.093 (0-50)	~	17273	17330	57	-	9.97	9.6	0.010

I051	17314	17364	50	-	0.093 (0-50)	~	17332	17390	58	-	10.98	10.78	0.005
I052	2087250	2087325	75	+	0.092 (0-50), 0.038 (25-75)	2087241-2087360 + Carter et al.,<br>2087284-2087363 - Carter et al.	2087235	2087402	167	+	9.79	9.79	0.000
I053	623833	623883	50	-	0.092 0.092 (0-50)	623784-623903 + Carter et al.,<br>623829-624038 - Chen et al.	622282	624056	1774	-	11.26	11.09	0.001
I054	1293467	1293524	57	-	0.073 0.059 (0-50), 0.092 (7-57)	1293409-1293488 - Carter et al.	1293384	1293505	121	-	10.09	9.97	0.002
I055	557064	557114	50	-	0.092 0.071 (0-50)	~	557077	557139	62	-	9.98	9.94	0.001
I056	931648	931698	50	+	0.09 0.088 (0-50)	931688-931767 - Carter et al.	931590	939799	8209	+	12.17	12.15	0.000
I057	4177361	4177436	75	+	0.089 (0-50), 0.04 (25-75)	4177248-4177547 - Tjaden et al.,<br>4177322-4177441 + Carter et al.,<br>4177364-4177443 - Carter et al.	4176401	4187628	11227	+	13.04	12.78	0.001
I234	312101	312151	50	+	0.019 (0-50)	~	312068	312123	55	+	9.84	9.66	0.005
I234	312101	312151	50	+	0.019 (0-50)	~	312130	312168	38	+	9.78	9.71	0.004
I272	3054984	3055034	50	+	0.0074 (0-50)	3054867-3055018 + Chen et al.,<br>3054958-3055037 - Carter et al.	3054863	3056476	1613	+	11	10.71	0.002
I235	1671625	1671675	50	+	0.019 (0-50)	~	1671567	1671663	96	+	9.67	9.53	0.003
I235	1671625	1671675	50	+	0.019 (0-50)	~	1671664	1671735	71	+	10.56	10.35	0.005
I201	4438892	4438942	50	+	0.027 (0-50)	~	4438798	4439297	499	+	9.83	9.8	0.000
I240	3774061	3774111	50	+	0.018 (0-50)	~	3770213	3774131	3918	+	10.14	10.17	0.000
I236	3723091	3723141	50	-	0.019 (0-50)	~	3720538	3723442	2904	-	11.38	11.35	0.000
I200	2076654	2076704	50	-	0.027 (0-50)	~	2076540	2076729	189	-	9.45	9.25	0.008
I237	4422544	4422596	52	+	0.014 (0-50), 0.019 (2-52)	~	4422538	4422604	66	+	9.71	9.58	0.004
I230	812270	812320	50	+	0.019 (0-50)	812251-812372 + Chen et al.,<br>812251-812370 + Carter et al.	812273	812334	61	+	10.68	10.72	0.001
I209	4006562	4006612	50	+	0.025 (0-50)	4006513-4006565 - Carter et al.	4003842	4006585	2743	+	10.79	10.53	0.002
I231	4501349	4501399	50	+	0.019 (0-50)	4501193-4501457 + Chen et al.	4501327	4501438	111	+	9.45	9.53	0.002
I133	2875841	2875916	75	+	0.05 (0-50), 0.033 (25-75)	2875792-2875951 - Carter et al.	2874565	2875883	1318	+	10.39	10.27	0.001
I132	190699	190757	58	+	0.0041 (0-50), 0.05 0.027 (8-58)	190613-190847 - Tjaden et al.	189776	194806	5030	+	12.4	12.29	0.000
I131	1500331	1500381	50	+	0.052 (0-50)	~	1499692	1500809	1117	+	10.71	10.78	0.000
I130	1948673	1948723	50	-	0.052 (0-50)	1948618-1948806 - Tjaden et al.	1944734	1948847	4113	-	11.61	11.42	0.001
I137	3382038	3382138	100	-	0.048 (0-50), 0.021 (50-100)	3381939-3382059 + Tjaden et al.	3380502	3382191	1689	-	10.29	10.39	0.001
I136	2076731	2076806	75	+	0.048 (0-50), 0.0022 (25-75)	2076717-2076796 + Carter et al.	2076715	2076790	75	+	9.6	9.52	0.002
I135	1525401	1525451	50	+	0.048 (0-50)	1525417-1525496 + Carter et al.	1525424	1525501	77	+	9.51	9.53	0.000
I134	12011	12061	50	+	0.049 (0-50)	~	12044	16873	4829	+	11.33	11.24	0.000
I139	2244889	2244939	50	+	0.048 (0-50)	~	2244900	2244968	68	+	9.61	9.21	0.010
I138	1864621	1864671	50	+	0.048 (0-50)	~	1864636	1864677	41	+	9.77	9.57	0.008
I304	4609820	4609870	50	+	0.00058 (0-50)	4609572-4609981 - + Rivas et al.	4609267	4610142	875	+	10.83	10.58	0.002
I049	127712	127812	100	+	0.093 0.038 (0-50), 0.011 (25-75),<br>0.065 0.065 (50-100)	127788-127861 + Carter et al.	127706	129241	1535	+	11.96	11.69	0.002
I048	1360592	1360642	50	+	0.094 0.065 (0-50)	1360573-1360652 + Carter et al.	1360555	1360604	49	+	10.92	10.66	0.009
I048	1360592	1360642	50	+	0.094 0.065 (0-50)	1360573-1360652 + Carter et al.	1360602	1360660	58	+	11.01	10.94	0.003
I238	3645983	3646033	50	-	0.018 (0-50)	3645948-3646027 - Carter et al.	3645967	3646015	48	-	13.28	13.14	0.003
I239	1957154	1957204	50	+	0.018 0.013 (0-50)	~	1957131	1957186	55	+	9.5	9.43	0.002
I043	2539548	2539599	51	+	0.011 (0-50), 0.1 (1-51)	~	2539537	2539586	49	+	9.8	9.22	0.023
I043	2539548	2539599	51	+	0.011 (0-50), 0.1 (1-51)	~	2539583	2539646	63	+	9.86	9.88	0.001
I042	3697867	3697967	100	-	0.036 (0-50), 0.1 (50-100)	3697742-3697968 - Tjaden et al.,<br>3697782-3697901 - Carter et al.	3695865	3698030	2165	-	9.83	9.62	0.002
I041	931643	931718	75	-	0.098 0.021 (0-50), 0.1 (25-75)	931688-931767 - Carter et al.	931640	931751	111	-	10.79	10.74	0.001
I040	753791	753850	59	-	0.066 (0-50), 0.1 (9-59)	753782-753861 + Carter et al.	752383	753959	1576	-	11.56	11.3	0.002
I047	1349163	1349238	75	+	0.022 (0-50), 0.097 (25-75)	1349187-1349375 + Chen et al.,<br>1349231-1349310 - Carter et al.	1349154	1349392	238	+	10.75	10.44	0.004
I046	3834155	3834230	75	-	0.032 (0-50), 0.098 (25-75)	~	3834202	3834375	173	-	10.34	10.28	0.001
I044	4366175	4366225	50	+	0.1 0.024 (0-50)	~	4364857	4366492	1635	+	10.24	10.01	0.002
I228	753925	753975	50	-	0.02 (0-50)	~	752383	753959	1576	-	11.56	11.3	0.002
I270	3279366	3279416	50	+	0.0079 (0-50)	~	3278818	3279613	795	+	10.15	9.98	0.002
I303	2876041	2876091	50	+	0.00058 (0-50)	2876042-2876121 + Carter et al.,<br>2876072-2876151 - Carter et al.	2875920	2876131	211	+	10.17	10.06	0.002

I171	2302513	2302563	50	+	0.034 0.0088 (0-50)	2302448-2302527 - Carter et al.,<br>2302528-2302647 - Carter et al.	2301879	2303060	1181	+	10.97	10.97	0.000
I302	312626	312676	50	+	0.00059 (0-50)	312612-312691 + Carter et al.	312612	312656	44	+	9.22	9.04	0.008
I302	312626	312676	50	+	0.00059 (0-50)	312612-312691 + Carter et al.	312657	312746	89	+	10.01	9.9	0.003
I076	2137631	2137681	50	+	0.074 0.031 (0-50)	2137548-2137667 + Carter et al.	2135941	2137743	1802	+	10.86	10.52	0.003
I077	2474423	2474473	50	+	0.074 (0-50)	2474407-2474539 + Tjaden et al.	2474411	2474505	94	+	13.68	12.86	0.013
I074	710008	710058	50	-	0.077 (0-50)	709961-710159 - + Rivas et al.	709997	710097	100	-	13.17	12.9	0.004
I075	4529699	4529774	75	+	0.0062 (0-50), 0.075 0.015 (25-75)	4529685-4529764 - Carter et al.,<br>4529766-4529862 + Tjaden et al.	4528875	4529748	873	+	9.8	9.51	0.005
I075	4529699	4529774	75	+	0.0062 (0-50), 0.075 0.015 (25-75)	4529685-4529764 - Carter et al.,<br>4529766-4529862 + Tjaden et al.	4529756	4529897	141	+	10.09	9.3	0.013
I072	608500	608557	57	-	0.00058 (0-50), 0.078 (7-57)	~	607308	611880	4572	-	11.78	11.61	0.001
I070	2588903	2588978	75	+	0.00058 (0-50), 0.079 (25-75)	~	2588920	2589088	168	+	9.87	9.96	0.001
I071	77471	77521	50	+	0.078 (0-50)	77461-77533 + Tjaden et al.	77356	79044	1688	+	10.64	10.59	0.000
I107	4327916	4327980	64	+	0.0017 (0-50), 0.061 (14-64)	4327822-4328067 - + Rivas et al.,<br>4327950-4328029 - Carter et al.	4325245	4328300	3055	+	11.81	11.5	0.002
I104	214066	214116	50	-	0.064 (0-50)	214001-214080 - Carter et al.	213678	214196	518	-	11.54	11.67	0.001
I102	1407157	1407207	50	-	0.066 (0-50)	~	1406783	1407158	375	-	10.08	9.75	0.006
I078	311135	311185	50	+	0.074 (0-50)	~	311062	311156	94	+	9.97	9.8	0.004
I079	781108	781208	100	-	0.055 (0-50), 0.073 0.045 (50-100)	781029-781252 - Chen et al.	781155	781222	67	-	10.36	10.28	0.002
I186	1860603	1860653	50	+	0.031 (0-50)	1860602-1860796 - + Rivas et al.,<br>1860608-1860782 - Argaman et al.	1860583	1862663	2080	+	12.71	12.54	0.001
I119	637956	638006	50	+	0.054 (0-50)	637912-638025 + Chen et al.	637891	638850	959	+	12.7	12.49	0.001
I182	1634881	1634931	50	-	0.032 (0-50)	~	1633814	1634933	1119	-	9.88	9.8	0.003
I183	2453791	2453841	50	+	0.032 (0-50)	~	2453790	2453828	38	+	9.33	9.36	0.001
I118	4516681	4516781	100	-	0.03 (0-50), 0.055 (50-100)	~	4516476	4517277	801	-	9.38	9.21	0.006
I226	2302863	2302913	50	+	0.02 (0-50)	2302768-2302927 - Carter et al.	2301879	2303060	1181	+	10.97	10.97	0.000
I225	3850993	3851068	75	-	0.021 (0-50), 0.015 (25-75)	~	3848827	3851045	2218	-	10.56	10.62	0.001
I224	2076579	2076629	50	-	0.021 (0-50)	~	2076540	2076729	189	-	9.45	9.25	0.008
I223	3352117	3352167	50	+	0.022 (0-50)	~	3351554	3352243	689	+	10.94	10.74	0.002
I222	608525	608582	57	+	0.0034 (0-50), 0.022 0.0069 (7-57)	~	607124	608681	1557	+	10.8	10.68	0.002
I188	1735439	1735514	75	+	0.014 (0-50), 0.031 (25-75)	~	1735430	1735468	38	+	10.17	10.09	0.003
I188	1735439	1735514	75	+	0.014 (0-50), 0.031 (25-75)	~	1735478	1735539	61	+	9.45	9.46	0.000
I221	3637575	3637625	50	+	0.022 (0-50)	3637601-3637680 + Carter et al.	3635547	3637596	2049	+	10.73	10.46	0.002
I249	1439133	1439183	50	+	0.015 (0-50)	~	1439042	1439765	723	+	11.21	11.12	0.001
I258	59404	59454	50	+	0.012 (0-50)	59360-59439 + Carter et al.	59338	59492	154	+	11.49	11.07	0.005
I160	1268547	1268597	50	-	0.039 (0-50)	1268489-1268738 - + Rivas et al.,<br>1268532-1268691 - Carter et al.	1268279	1269894	1615	-	10.22	10.03	0.007
I259	4177286	4177336	50	+	0.012 (0-50)	4177242-4177321 + Carter et al.,<br>4177248-4177547 - Tjaden et al.,<br>4177322-4177441 + Carter et al.	4176401	4187628	11227	+	13.04	12.78	0.001
I161	2342660	2342735	75	-	0.0078 (0-50), 0.039 (25-75)	~	2342642	2342696	54	-	9.84	9.66	0.006
I161	2342660	2342735	75	-	0.0078 (0-50), 0.039 (25-75)	~	2342697	2342743	46	-	10.5	10.09	0.015
I063	2559093	2559143	50	+	0.086 0.0079 (0-50)	~	2559084	2559131	47	+	10.4	10.36	0.001
I062	312101	312156	55	-	0.086 (0-50), 0.061 0.052 (5-55)	~	312092	312154	62	-	9.41	9.41	0.000
I065	2137607	2137657	50	-	0.083 0.038 (0-50)	2137548-2137667 + Carter et al.	2136595	2140967	4372	-	11.07	10.87	0.001
I064	271729	271779	50	+	0.083 (0-50)	271640-271799 + Carter et al.	269421	271808	2387	+	10.63	10.65	0.000
I067	754175	754225	50	-	0.081 (0-50)	754150-754229 - Carter et al.	754152	754245	93	-	11.06	10.71	0.007
I066	1268797	1268847	50	-	0.082 (0-50)	~	1268279	1269894	1615	-	10.22	10.03	0.007
I069	2751665	2751715	50	-	0.079 (0-50)	2751490-2751789 + Tjaden et al.,<br>2751645-2751764 - Carter et al.	2751661	2751792	131	-	9.83	9.78	0.001
I113	578257	578307	50	+	0.058 (0-50)	~	578256	578930	674	+	10.17	10.02	0.002
I115	3067952	3068002	50	+	0.058 (0-50)	~	3067964	3068010	46	+	10.82	11.08	0.012
I114	2076406	2076506	100	+	0.058 (0-50), 0.046 (50-100)	~	2076271	2076419	148	+	10.76	10.37	0.006
I114	2076406	2076506	100	+	0.058 (0-50), 0.046 (50-100)	~	2076419	2076456	37	+	10.12	9.99	0.006
I114	2076406	2076506	100	+	0.058 (0-50), 0.046 (50-100)	~	2076494	2076710	216	+	10	9.84	0.004
I116	3923486	3923536	50	+	0.058 (0-50)	~	3920938	3923542	2604	+	10.6	10.3	0.002
I191	3930766	3930816	50	-	0.03 (0-50)	~	3930776	3931249	473	-	9.35	9.3	0.001

I190	279234	279284	50	-	0.03 (0-50)	~	278024	280043	2019	-	10.59	10.71	0.001
I193	1739287	1739337	50	-	0.029 0.022 (0-50)	1739222-1739379 + Chen et al., 1739267-1739346 - Carter et al.	1739251	1739289	38	-	9.14	9.16	0.001
I193	1739287	1739337	50	-	0.029 0.022 (0-50)	1739222-1739379 + Chen et al., 1739267-1739346 - Carter et al.	1739291	1740311	1020	-	9.7	9.46	0.003
I192	2378563	2378613	50	+	0.029 (0-50)	2378529-2378608 + Carter et al., 2378534-2378740 + Tjaden et al., 2378544-2378743 - + Rivas et al.	2377336	2378691	1355	+	10.05	9.86	0.002
I195	2065843	2065893	50	+	0.029 (0-50)	~	2065863	2065931	68	+	9.91	9.18	0.021
I194	728057	728107	50	-	0.029 (0-50)	~	727998	728069	71	-	10.22	10.05	0.004
I194	728057	728107	50	-	0.029 (0-50)	~	728100	728142	42	-	10.67	10.44	0.008
I197	2064136	2064186	50	+	0.028 (0-50)	~	2064117	2064196	79	+	10.49	9.89	0.014
I196	3119420	3119470	50	+	0.028 (0-50)	3119392-3119548 + Chen et al.	3119393	3120194	801	+	10.15	9.98	0.002
I199	631362	631412	50	-	0.027 (0-50)	631320-631487 - Tjaden et al.	631240	631455	215	-	9.72	9.5	0.004
I198	4516595	4516645	50	+	0.027 (0-50)	4516371-4516650 + Carter et al.	4516333	4517332	999	+	11.76	10.93	0.008
I210	4498549	4498603	54	-	0.025 (0-50), 0.018 (4-54)	4498468-4498595 - Tjaden et al., 4498538-4498617 - Carter et al.	4498020	4499072	1052	-	10.6	10.32	0.002
I211	214141	214191	50	-	0.025 (0-50)	~	213678	214196	518	-	11.54	11.67	0.001
I216	281307	281357	50	-	0.023 (0-50)	281271-281350 - Carter et al., 281298-281377 + Carter et al., 281311-281390 - Carter et al.	281233	281338	105	-	9.98	10.04	0.001
I217	1525414	1525464	50	-	0.023 (0-50)	1525417-1525496 + Carter et al.	1525424	1525482	58	-	9.93	9.84	0.002
I215	3645906	3645956	50	+	0.023 (0-50)	3645948-3646027 - Carter et al.	3645892	3645956	64	+	10.68	10.33	0.010
I292	3773917	3773967	50	-	0.0029 (0-50)	~	3773538	3774536	998	-	11	11.23	0.002
I296	1634866	1634916	50	+	0.0022 (0-50)	~	1633846	1634941	1095	+	10.03	9.95	0.002
I297	2175355	2175405	50	+	0.0017 (0-50)	2175319-2175434 + Tjaden et al., 2175335-2175430 - Chen et al.	2175289	2176693	1404	+	10.22	10.12	0.001
I295	1644973	1645023	50	-	0.0022 (0-50)	1644948-1645054 - Tjaden et al., 1644975-1645147 - Chen et al.	1644840	1645151	311	-	12.03	11.74	0.003
I298	3119395	3119445	50	-	0.0017 (0-50)	3119392-3119548 + Chen et al.	3119393	3119546	153	-	10.28	9.83	0.007
I299	4516695	4516745	50	+	0.0017 (0-50)	~	4516333	4517332	999	+	11.76	10.93	0.008
I288	4569609	4569659	50	+	0.0034 (0-50)	4569550-4569682 + Chen et al.	4569613	4569679	66	+	10.13	9.67	0.013
I252	1501306	1501356	50	-	0.014 (0-50)	~	1501277	1501346	69	-	10.19	10.07	0.003
I218	1501381	1501431	50	-	0.023 (0-50)	~	1501363	1501408	45	-	10.51	10.43	0.003
I205	3697667	3697742	75	-	0.026 (0-50), 0.022 (25-75)	3697742-3697968 - Tjaden et al.	3695865	3698030	2165	-	9.83	9.62	0.002
I204	2885426	2885476	50	-	0.026 (0-50)	2885306-2885437 + Chen et al.	2885380	2885432	52	-	9.61	9.6	0.000
I204	2885426	2885476	50	-	0.026 (0-50)	2885306-2885437 + Chen et al.	2885439	2885520	81	-	10.57	10.47	0.002
I207	2051450	2051500	50	-	0.026 (0-50)	~	2051387	2051631	244	-	10.87	10.65	0.002
I206	1762535	1762585	50	+	0.026 (0-50)	~	1762464	1762583	119	+	9.9	9.42	0.009
I098	2698313	2698363	50	-	0.068 (0-50)	~	2698243	2698406	163	-	12.39	11.82	0.007
I099	3126136	3126186	50	-	0.068 (0-50)	~	3126107	3126170	63	-	10.84	10.83	0.000
I203	4372012	4372062	50	+	0.026 (0-50)	4371910-4372189 + Chen et al.	4372044	4372098	54	+	9.22	9.12	0.003
I202	2773242	2773342	100	+	0.026 (0-50), 0.026 (25-75), 0.022 (50-100)	2773203-2773322 + Carter et al.	2773276	2773357	81	+	11	9.98	0.022
I094	3054973	3055023	50	-	0.068 0.0099 (0-50)	3054867-3055018 + Chen et al., 3054958-3055037 - Carter et al.	3054960	3055039	79	-	10.14	10.05	0.002
I096	1019376	1019426	50	-	0.068 (0-50)	~	1018181	1019425	1244	-	13.63	13.58	0.000
I097	1269322	1269372	50	-	0.068 (0-50)	~	1268279	1269894	1615	-	10.22	10.03	0.007
I090	753791	753841	50	+	0.068 (0-50)	753782-753861 + Carter et al.	753789	753847	58	+	10.15	10.17	0.001
I092	2902046	2902096	50	+	0.068 0.018 (0-50)	2902089-2902168 - Carter et al.	2902013	2902106	93	+	10.28	10.12	0.004
I093	1268347	1268422	75	-	0.004 (0-50), 0.068 (25-75)	1268332-1268451 - Carter et al.	1268279	1269894	1615	-	10.22	10.03	0.007
I014	4373943	4374003	60	-	0.14 0.066 (0-50), 0.027 (10-60)	~	4372615	4374522	1907	-	10.74	10.55	0.001
I015	1342470	1342520	50	+	0.14 0.12 (0-50)	1342431-1342640 - Chen et al.	1342397	1342644	247	+	10.64	10.65	0.000
I016	1218274	1218324	50	-	0.14 (0-50)	~	1218264	1218328	64	-	9.89	9.85	0.001
I017	1391014	1391080	66	-	0.022 (0-50), 0.13 (16-66)	~	1390940	1391112	172	-	9.68	9.69	0.000
I010	4527911	4527961	50	+	0.15 (0-50)	4527862-4527941 + Carter et al.	4527764	4527945	181	+	9.49	9.43	0.002
I011	3705984	3706059	75	+	0.14 (0-50), 0.036 (25-75)	3705925-3706044 - Carter et al.	3705916	3706086	170	+	9.83	9.36	0.007
I012	3723097	3723172	75	+	0.034 (0-50), 0.14 0.033 (25-75)	3723150-3723337 - Tjaden et al.	3722997	3723610	613	+	10.26	10.06	0.002

I013	1195915	1195990	75	-	0.038 0.0034 (0-50), 0.14 (25-75)	~	1195876	1197489	1613	-	10.76	10.46	0.003
I164	4160968	4161018	50	-	0.038 (0-50)	4160940-4161059 + Carter et al.	4158786	4161346	2560	-	11.14	10.69	0.003
I165	59529	59579	50	+	0.038 (0-50)	~	59532	59742	210	+	10.26	9.86	0.006
I166	569751	569801	50	+	0.038 (0-50)	~	569755	569792	37	+	9.67	9.35	0.017
I167	1913455	1913505	50	-	0.037 (0-50)	~	1910728	1914199	3471	-	12.45	12.24	0.001
I019	1197743	1197793	50	-	0.13 0.05 (0-50)	~	1197721	1197803	82	-	9.28	9.33	0.001
I162	2302638	2302688	50	+	0.038 (0-50)	2302528-2302647 - Carter et al., 2302648-2302767 - Carter et al.	2301879	2303060	1181	+	10.97	10.97	0.000
I163	1488640	1488690	50	-	0.038 (0-50)	1488549-1488713 + Tjaden et al., 1488680-1488759 - Carter et al.	1488348	1488828	480	-	14.06	13.79	0.001
I212	4158403	4158453	50	+	0.025 (0-50)	~	4157738	4158819	1081	+	10.17	9.88	0.004
I260	1391039	1391089	50	+	0.012 (0-50)	~	1390957	1391112	155	+	11.1	10.98	0.002
I289	4532058	4532108	50	-	0.0034 (0-50)	~	4532083	4532163	80	-	10.15	10.04	0.002
I283	2751577	2751627	50	+	0.0041 (0-50)	2751490-2751789 + Tjaden et al.	2751512	2752043	531	+	12.86	12.67	0.001
I213	4359929	4359979	50	-	0.024 (0-50)	4359749-4360129 + Chen et al.	4358387	4359985	1598	-	10.9	10.67	0.003
I285	1269722	1269772	50	-	0.004 (0-50)	1269546-1269837 - + Rivas et al., 1269612-1269731 - Carter et al.	1268279	1269894	1615	-	10.22	10.03	0.007
I284	4005466	4005516	50	+	0.0041 (0-50)	4005406-4005485 - Carter et al., 4005462-4005541 + Carter et al.	4003842	4006585	2743	+	10.79	10.53	0.002
I287	2065455	2065505	50	-	0.0035 (0-50)	~	2065310	2065509	199	-	11.56	11.25	0.004
I286	2763349	2763399	50	+	0.004 (0-50)	~	2763382	2763859	477	+	10.54	10.35	0.002
I281	2590892	2590942	50	-	0.0047 (0-50)	2590810-2590906 - Tjaden et al.	2590806	2590944	138	-	10.04	9.75	0.005
I280	3119450	3119500	50	-	0.0051 (0-50)	3119392-3119548 + Chen et al.	3119393	3119546	153	-	10.28	9.83	0.007
I282	223546	223596	50	-	0.0045 (0-50)	223489-223608 + Carter et al.	223217	229162	5945	-	10.23	10.08	0.002

Supplementary Table S3

Differentially expressed transcripts that overlapped with the ncRNAs presented by Hershberg *et al.* (2003)

Transcripts detected using the sliding window method. All predicted ncRNAs from the list compiled by Hersberg *et al.* that overlaps with differentially expressed regions in this study. Columns 1 through 7 are data from the Hershberg list. Columns 8 through 16 are from generated from in this study. Signal intensity values and fold change are log_2 values.

Candidate name	Predicted by	Start	End	Length	Strand	Upstream gene / Downstream gene (+info)	Fold change this study	Length this study	Start this study	End this study	Strand this study	P(diff reg)	Regulation	Ref signal	Stress signal
C0345	Tjaden et al.	1417525	1417597	72	-	ydaG/racR	-0.55	25	1417526	1417551	-	0.97	DOWN	11.59	11.04
HB_277	Carter et al.	4532323	4532402	79	+	yjhQ/yjhR	-1.09	85	4532242	4532327	+	1	DOWN	12.11	11.02
C0042	Tjaden et al.	174939	175081	142	+	hemL/yadQ	-0.85	60	174989	175049	+	1	DOWN	13.38	12.54
C0880	Tjaden et al.	3733853	3733953	100	-	xylR/bax	-0.77	330	3733824	3734154	-	1	DOWN	10.83	10.07
te3	Rivas et al.	1553785	?	?	unknown	sfcA/yddX IS070 1553777-1554007 - Score: 17.63 Overlaps a 5' UTR	-1.29	49	1553760	1553809	-	1	DOWN	11.28	9.98
C0851	Tjaden et al.	3645700	3645859	159	+	gor/arsR	-0.92	25	3645680	3645705	+	1	DOWN	10.55	9.63
C0851	Tjaden et al.	3645700	3645859	159	+	gor/arsR	-0.90	25	3645828	3645853	+	1	DOWN	10.26	9.36
e1	Rivas et al.	4437372	?	?	unknown	ytfK/ytfL Score: 16.84	-0.84	51	4437326	4437377	+	1	DOWN	12.19	11.35
HB_449	Carter et al.	3054958	3055037	79	-	ygfA/serA	-0.91	60	3055024	3055084	-	1	DOWN	10.81	9.91
tp55	Rivas et al.	450836	?	?	unknown	cyoA/ampG Score: 5.16	-1.23	200	450661	450861	-	1	DOWN	10.73	9.51
IS115	Chen et al.	2403317	2403693	376	+	nuoA/lrhA k30 2403505 C0543 2403551-2403694 +	-1.01	33	2403446	2403479	+	1	DOWN	11.55	10.54
HB_86	Carter et al.	1328903	1328982	79	+	yciN/topA HB_87 1328943-1329021 + Overlaps a 5' UTR	-0.92	33	1328928	1328961	+	1	DOWN	12.56	11.64
HB_87	Carter et al.	1328943	1329021	78	+	yciN/topA HB_86 1328903-1328982 + Overlaps a 5' UTR	-0.92	33	1328928	1328961	+	1	DOWN	12.56	11.64
IS182	Chen et al.	3655606	3655816	210	-	hdeD/yhiE C0857 3655198-3655608 -	-1.58	154	3655638	3655792	-	1	DOWN	11.98	10.41
tpke79	Rivas et al.	1921027	?	?	unknown	pphA/yebY Score: 11.82	-1.11	270	1920760	1921030	-	1	DOWN	12.48	11.37
HB_74	Carter et al.	924764	924843	79	+	clpA/serW	-1.07	74	924805	924879	+	1	DOWN	10.24	9.17
HB_354	Carter et al.	1407365	1407444	79	-	ydaN/dbpA	-0.78	104	1407276	1407380	-	1	DOWN	10.79	10.01
t70	Rivas et al.	925385	?	?	unknown	serW/infA Score: 5.57	-1.39	90	925313	925403	-	1	DOWN	12.19	10.80
p11	Rivas et al.	3309327	?	?	unknown	rpsO/truB Score: 11.55 Overlaps a 5' UTR	-0.81	39	3309322	3309361	-	1	DOWN	12.05	11.25
C0215	Tjaden et al.	924883	924970	87	+	clpA/serW	-0.63	33	924920	924953	+	0.97	DOWN	11.17	10.54
C0294	Tjaden et al.	1223306	1223396	90	-	ycgI/minE	-0.96	38	1223371	1223409	-	1	DOWN	11.56	10.60
C0510	Tjaden et al.	2226932	2227408	476	+	yohG/yohI k3 2227055	-0.98	40	2227026	2227066	+	1	DOWN	12.13	11.15
C1070	Tjaden et al.	4532142	4532310	168	+	yjhQ/yjhR	-1.09	85	4532242	4532327	+	1	DOWN	12.11	11.02
psrA9	Argaman et al.	2531422	2531608	186	-	cysK/ptsH	-1.27	66	2531494	2531560	-	1	DOWN	10.81	9.54
C0304	Tjaden et al.	1260026	1260101	75	-	ychM/prs	-1.16	98	1260029	1260127	-	1	DOWN	10.88	9.72
IS022	Chen et al.	584964	585220	256	+	ompT/envY	-0.73	51	584995	585046	+	0.97	DOWN	11.39	10.66
IS026	Chen et al.	698444	698601	157	-	asnB/nagD	-1.36	36	698490	698526	-	1	DOWN	11.49	10.14
t48	Rivas et al.	1140213	?	?	unknown	flgL/rne Score: 7.55	-0.72	37	1140213	1140250	+	0.99	DOWN	11.68	10.96
IS109	Chen et al.	2190859	2190978	119	-	yehE/mrp	-0.79	25	2190917	2190942	-	0.99	DOWN	11.49	10.70
IS073	Chen et al.	1630377	1630602	225	+	ydfJ/ydfK	-1.52	87	1630469	1630556	+	1	DOWN	11.62	10.10
HB_155	Carter et al.	2773243	2773322	79	+	yfjW/ypjI HB_154 2773203-2773282 +	-0.93	91	2773201	2773292	+	1	DOWN	11.89	10.96
HB_154	Carter et al.	2773203	2773282	79	+	yfjW/ypjI HB_155 2773243-2773322 +	-0.93	91	2773201	2773292	+	1	DOWN	11.89	10.96
tpke37	Rivas et al.	2702135	?	?	unknown	rnc/lepB Score: 11.11 Experimentally tested Overlaps a 5' UTR	-0.79	48	2702112	2702160	-	1	DOWN	13.09	12.30
C0485	Tjaden et al.	2060175	2060249	74	-	nac/asnV	-1.18	32	2060193	2060225	-	1	DOWN	11.16	9.98
C0645	Tjaden et al.	2751490	2751789	299	+	Not intergenic	1.00	487	2751036	2751523	+	1	UP	10.76	11.76
IS067	Chen et al.	1489461	1489633	172	-	cybB/ydcA	-1.07	211	1489291	1489502	-	1	DOWN	11.08	10.00
IS062	Chen et al.	1432742	1433125	383	-	ynaE/ynaF	-1.39	344	1432785	1433129	-	1	DOWN	12.39	11.00
C0325	Tjaden et al.	1321159	1321239	80	-	trpL/trpH	-0.79	41	1321142	1321183	-	0.99	DOWN	10.87	10.08

k30	Rivas et al.	2403505	?	?	unknown	nuoA/lrhA IS115 2403317-2403693 + Score: 7.95 Overlaps a 5' UTR	-1.44	73	2403446	2403519	-	1	DOWN	11.61	10.17
k3	Rivas et al.	2227055	?	?	unknown	yohG/yohI C0510 2226932-2227408 + Score: 20.83 Experimentally tested	-0.98	40	2227026	2227066	+	1	DOWN	12.13	11.15
k2	Rivas et al.	914218	?	?	unknown	ybjE/aqpZ Score: 21.47 Experimentally tested	-1.15	48	914191	914239	+	1	DOWN	11.00	9.85
IS081	Chen et al.	1739222	1739379	157	+	ydhC/cfa Overlaps a 5' UTR	-1.17	25	1739240	1739265	+	0.99	DOWN	13.16	11.98
C0966	Tjaden et al.	4173141	4173244	103	-	tyrU/glyT	-1.38	25	4173154	4173179	-	1	DOWN	10.63	9.25
C0566	Tjaden et al.	2474407	2474539	132	+	yfdT/dsdC	-0.67	65	2474488	2474553	+	1	DOWN	14.29	13.62
C0066	Tjaden et al.	237091	237297	206	+	aspV/yafT	-0.94	71	237044	237115	+	1	DOWN	11.55	10.61
C0382	Tjaden et al.	1640115	1640303	188	-	cspF/ydfT	-0.62	73	1640269	1640342	-	1	DOWN	13.06	12.45
C0243	Tjaden et al.	989694	989812	118	+	pncB/pepN	-1.04	32	989711	989743	+	1	DOWN	11.57	10.52
tk1	Rivas et al.	3330379	?	?	unknown	yhbE/rpmA Score: 17.89 Experimentally tested	-0.72	684	3329834	3330518	-	1	DOWN	11.56	10.84
IS070	Chen et al.	1553777	1554007	230	-	sfcA/yddX te3 1553785 Overlaps a 5' UTR Resides within an operon	-1.29	49	1553760	1553809	-	1	DOWN	11.28	9.98
p29	Rivas et al.	4103904	?	?	unknown	cpxP/yiiP HB_526 4103901-4103958 - Score: 5.54	-1.37	318	4103807	4104125	+	1	DOWN	13.51	12.13
C0757	Tjaden et al.	3315732	3315825	93	-	yhbC/metY	-0.87	105	3315702	3315807	-	1	DOWN	12.41	11.54
IS094	Chen et al.	1994968	1995087	119	+	sdiA/yecC HB_115 1995026-1995084 +	-1.43	32	1994975	1995007	+	1	DOWN	11.89	10.46
C0376	Tjaden et al.	1636223	1636310	87	-	ydfY/cspI	-2.81	469	1635861	1636330	-	1	DOWN	12.72	9.91
p32	Rivas et al.	2465734	?	?	unknown	intS/yfdG Score: 5.03	-0.89	209	2465555	2465764	-	0.99	DOWN	11.78	10.90
IS042	Chen et al.	1014724	1014910	186	+	ymbA/rmf	-0.80	25	1014870	1014895	+	0.95	DOWN	13.77	12.96
IS129	Chen et al.	2651687	2652078	391	-	sseA/sseB HB_418 2651857-2651936 -	-3.17	40	2651665	2651705	-	0.98	DOWN	12.67	9.50
IS129	Chen et al.	2651687	2652078	391	-	sseA/sseB HB_418 2651857-2651936 -	-0.76	70	2652032	2652102	-	0.99	DOWN	10.38	9.61
IS122	Chen et al.	2496319	2496526	207	-	yfdZ/ypdA	-0.96	88	2496524	2496612	-	1	DOWN	10.84	9.88
HB_482	Carter et al.	3645948	3646027	79	-	gor/arsR	0.64	92	3645892	3645984	-	1	UP	13.57	14.21
C0506	Tjaden et al.	2213677	2213763	86	+	yehV/yehW	-0.92	41	2213669	2213710	+	1	DOWN	10.21	9.29
HB_346	Carter et al.	1286602	1286680	78	-	tyrV/tyrT HB_345 1286641-1286720 -	-0.91	88	1286602	1286690	-	0.99	DOWN	12.19	11.28
HB_344	Carter et al.	1286681	1286760	79	-	tyrV/tyrT HB_345 1286641-1286720 -	-0.91	88	1286602	1286690	-	0.99	DOWN	12.19	11.28
HB_345	Carter et al.	1286641	1286720	79	-	tyrV/tyrT HB_344 1286681-1286760 - HB_346 1286602-1286680 -	-0.91	88	1286602	1286690	-	0.99	DOWN	12.19	11.28

Supplementary Table S4

Similarly expressed transcripts that overlapped with the ncRNA presented by Hershberg *et al.* (2003)

Transcripts detected using the sliding window method. All predicted ncRNAs from the list compiled by Hersberg *et al.* that overlaps with similarly expressed regions in this study. Columns 1 through 7 contain data from the Hershberg list. Columns 8 through 14 contain data from this study. Signal intensity values are $\log_2$ values.

Candidate name	Predicted by	Start	End	Length	Strand	Upstream gene / Downstream gene (+ info)	Start this study	End this study	Length this study	Strand this study	Ref signal	Stress signal	P(diff reg)
C0340	Tjaden et al.	1368076	1368174	98	-	pspE/ycjM	1368048	1368093	45	-	10.01	10.03	0.00
tpk13	Rivas et al.	1861792	?	?	unknown	gapA/yeaD Score: 11.64	1860583	1862663	2080	+	12.71	12.54	0.00
tke6	Rivas et al.	1797296	?	?	unknown	pheM/rplT Score: 8.06	1790236	1797387	7151	-	11.17	11.04	0.00
C0345	Tjaden et al.	1417525	1417597	72	-	ydaG/racR	1417534	1417667	133	-	10.63	10.2	0.01
C0318	Tjaden et al.	1290522	1290623	101	-	rssB/galU	1290492	1290550	58	-	9.83	9.73	0.00
C0318	Tjaden et al.	1290522	1290623	101	-	rssB/galU	1290568	1290607	39	-	10.58	10.53	0.00
C0347	Tjaden et al.	1439219	1439291	72	-	Not intergenic	1439212	1439278	66	-	10.94	10.24	0.02
C0347	Tjaden et al.	1439219	1439291	72	-	Not intergenic	1439284	1440906	1622	-	11.44	11.11	0.00
C0346	Tjaden et al.	1433171	1433207	36	+	ynaE/ynaF	1433023	1433687	664	+	10.68	10.33	0.00
HB_247	Carter et al.	4323530	4323609	79	+	yjdN/yjdM HB_246 4323490-4323569 +	4323473	4323658	185	+	9.97	9.89	0.00
HB_276	Carter et al.	4531483	4531562	79	+	yjhQ/yjhR tpke10 4531556 IS220 4531472-4531746 +	4531299	4531876	577	+	10.77	10.57	0.00
p17	Rivas et al.	4182853	?	?	unknown	rpoB/rpoC Score: 8.83 Resides within an operon	4176401	4187628	11227	+	13.04	12.78	0.00
p17	Rivas et al.	4182853	?	?	unknown	rpoB/rpoC Score: 8.83 Resides within an operon	4179235	4183352	4117	-	10.08	10.06	0.00
HB_244	Carter et al.	4323370	4323449	79	+	yjdN/yjdM HB_245 4323410-4323489 +	4323273	4323413	140	+	9.77	9.72	0.00
HB_277	Carter et al.	4532323	4532402	79	+	yjhQ/yjhR	4532317	4532408	91	+	11.81	11.22	0.01
C0187	Tjaden et al.	786899	786975	76	+	gpmA/galM	786898	787967	1069	+	10.39	10.33	0.00
IS163	Chen et al.	3267562	3267742	180	+	yhaC/yhaD IS164 3267675-3267855 +	3267476	3267620	144	+	9.61	9.31	0.01
IS163	Chen et al.	3267562	3267742	180	+	yhaC/yhaD IS164 3267675-3267855 +	3267681	3267898	217	+	10.01	9.94	0.00
C0712	Tjaden et al.	3096461	3096559	98	+	yggW/yggM	3093528	3096841	3313	+	10.67	10.61	0.00
C0183	Tjaden et al.	780667	780793	126	-	lysZ/lysQ	780690	781099	409	-	10.16	9.91	0.00
C0182	Tjaden et al.	779628	779747	119	-	ybgF/lysT IS031 779607-779718 -	779298	780458	1160	-	10.27	10.03	0.00
IS164	Chen et al.	3267675	3267855	180	+	yhaC/yhaD IS163 3267562-3267742 +	3267681	3267898	217	+	10.01	9.94	0.00
C0180	Tjaden et al.	776842	776939	97	-	tolA/tolB	775471	776908	1437	-	9.65	9.39	0.00
C0180	Tjaden et al.	776842	776939	97	-	tolA/tolB	776917	777190	273	-	9.93	9.86	0.00
C0594	Tjaden et al.	2562393	2562498	105	-	yffR/yffS	2562408	2562499	91	-	10.66	10.71	0.00
C0595	Tjaden et al.	2576422	2576619	197	-	maeB/talA	2574240	2576500	2260	-	10.19	10.12	0.00
C0595	Tjaden et al.	2576422	2576619	197	-	maeB/talA	2576548	2576637	89	-	9.83	9.88	0.00
IS168	Chen et al.	3411240	3411490	250	+	envR/acrE	3411329	3411463	134	+	10.19	9.98	0.01
C0189	Tjaden et al.	804999	805120	121	-	ybhJ/ybhC	804881	806551	1670	-	10.69	10.78	0.00
C0188	Tjaden et al.	793891	793971	80	-	modE/ybhT	791499	793974	2475	-	10.41	10.31	0.00
HB_248	Carter et al.	4323610	4323689	79	+	yjdN/yjdM	4323473	4323658	185	+	9.97	9.89	0.00
HB_246	Carter et al.	4323490	4323569	79	+	yjdN/yjdM HB_247 4323530-4323609 +	4323473	4323658	185	+	9.97	9.89	0.00
HB_539	Carter et al.	4244233	4244312	79	-	malE/malK HB_540 4244193-4244272 -	4241034	4244311	3277	-	10.02	10.13	0.00
C0817	Tjaden et al.	3494742	3494814	72	-	Not intergenic	3494565	3494830	265	-	9.97	9.93	0.00
tpk16	Rivas et al.	3107235	?	?	unknown	speC/yqgA Score: 10.39	3107032	3107286	254	+	10.04	10.19	0.00
tpk16	Rivas et al.	3107235	?	?	unknown	speC/yqgA Score: 10.39	3103628	3107328	3700	-	10.3	10.16	0.00
k52	Rivas et al.	484845	?	?	unknown	acrA/acrR Score: 5.09 Overlaps a 5' UTR	482671	484884	2213	+	9.95	9.8	0.00
k52	Rivas et al.	484845	?	?	unknown	acrA/acrR Score: 5.09 Overlaps a 5' UTR	480445	485300	4855	-	11.82	11.73	0.00
k51	Rivas et al.	107497	?	?	unknown	lpxC/srrA Score: 5.17	89565	107561	17996	+	11.89	11.95	0.00
k51	Rivas et al.	107497	?	?	unknown	lpxC/srrA Score: 5.17	107496	107561	65	-	9.44	9.32	0.00
tpe80	Rivas et al.	4609572	?	?	unknown	osmY/yjjU HB_562 4609571-4609609 - Score: 8.46	4609267	4610142	875	+	10.83	10.58	0.00
IS196	Chen et al.	4044340	4044455	115	-	yihG/polA	4042655	4044595	1940	-	10.62	10.41	0.00
HB_7	Carter et al.	63315	63394	79	+	hepA/polB	63297	63389	92	+	10.35	10.3	0.00

HB_7	Carter et al.	63315	63394	79	+	hepA/polB	63393	63455	62	+	9.91	9.73	0.01
HB_6	Carter et al.	59360	59439	79	+	yabQ/rluA Overlaps a 3' UTR	59338	59492	154	+	11.49	11.07	0.01
HB_5	Carter et al.	58260	58339	79	+	djlA/yabP HB_4 58220-58299 + Overlaps a 5' UTR	57193	58514	1321	+	10.8	10.46	0.00
HB_4	Carter et al.	58220	58299	79	+	djlA/yabP HB_5 58260-58339 + Overlaps a 5' UTR	57193	58514	1321	+	10.8	10.46	0.00
HB_3	Carter et al.	16548	16627	79	+	dnaJ/gef	12044	16873	4829	+	11.33	11.24	0.00
HB_2	Carter et al.	16228	16307	79	+	dnaJ/gef	12044	16873	4829	+	11.33	11.24	0.00
HB_1	Carter et al.	10585	10642	57	+	yaaH/yaaW	10550	10606	56	+	10	9.5	0.01
HB_1	Carter et al.	10585	10642	57	+	yaaH/yaaW	10639	10841	202	+	10.16	10.08	0.00
C1068	Tjaden et al.	4529766	4529862	96	+	sgcX/yjhP	4529756	4529897	141	+	10.09	9.3	0.01
C1066	Tjaden et al.	4518187	4518237	50	+	yjhU/yjhF HB_274 4518197-4518238 + HB_273 4518157-4518236 +	4518185	4518253	68	+	9.56	9.56	0.00
IS221	Chen et al.	4532323	4532459	136	-	yjhQ/yjhR	4532364	4532408	44	-	10.42	10.02	0.01
t13	Rivas et al.	4608582	?	?	unknown	prfC/osmY HB_561 4608573-4608634 - Score: 13.98	4605801	4609070	3269	+	10.54	10.45	0.00
t13	Rivas et al.	4608582	?	?	unknown	prfC/osmY HB_561 4608573-4608634 - Score: 13.98	4607679	4609099	1420	-	10.15	10.15	0.00
HB_9	Carter et al.	127788	127861	73	+	aceF/lpd Resides within an operon	127706	129241	1535	+	11.96	11.69	0.00
HB_8	Carter et al.	117680	117751	71	+	ppdD/nadC	117665	118160	495	+	9.88	9.83	0.00
C0049	Tjaden et al.	190613	190847	234	-	rpsB/tsf	189768	190739	971	-	10.34	10.31	0.00
C0049	Tjaden et al.	190613	190847	234	-	rpsB/tsf	190755	191791	1036	-	10.32	10.4	0.00
C0765	Tjaden et al.	3325738	3325824	86	-	yhbY/greA	3324973	3327254	2281	-	12.09	11.88	0.00
C0042	Tjaden et al.	174939	175081	142	+	hemL/yadQ	174948	175000	52	+	11.13	10.67	0.01
C0042	Tjaden et al.	174939	175081	142	+	hemL/yadQ	175032	176984	1952	+	12.36	12.2	0.00
p7	Rivas et al.	2847874	?	?	unknown	hycB/hycA Score: 14.84 Resides within an operon	2844994	2847898	2904	-	10.15	9.83	0.00
C0292	Tjaden et al.	1195705	1195804	99	-	icd/ymfD IS050 1195609-1195836 -	1195683	1195773	90	-	9.45	9.17	0.01
IS018	Chen et al.	573636	573747	111	+	ybcQ/nmpC HB_41 573643-573722 +	573661	573703	42	+	9.53	9.39	0.01
IS019	Chen et al.	576356	576507	151	+	nmpC/essD C0129 576300-576615 +	576302	576427	125	+	10.82	10.15	0.01
IS019	Chen et al.	576356	576507	151	+	nmpC/essD C0129 576300-576615 +	576432	577057	625	+	11.12	10.83	0.00
t6	Rivas et al.	932314	?	?	unknown	lrp/ftsK Score: 19.45 Experimentally tested	931590	939799	8209	+	12.17	12.15	0.00
IS014	Chen et al.	480028	480388	360	+	ybaJ/acrB	480072	480113	41	+	10.06	10.06	0.00
IS014	Chen et al.	480028	480388	360	+	ybaJ/acrB	480378	480416	38	+	9.81	9.56	0.01
IS012	Chen et al.	454059	454263	204	-	bolA/tig psrA2 454066-454262 -	454056	454136	80	-	10.02	9.89	0.00
IS012	Chen et al.	454059	454263	204	-	bolA/tig psrA2 454066-454262 -	454141	454242	101	-	9.95	9.69	0.01
t3	Rivas et al.	1755683	?	?	unknown	lpp/ynhG Score: 23.48 Experimentally tested	1755406	1756833	1427	+	11.68	11.58	0.00
t3	Rivas et al.	1755683	?	?	unknown	lpp/ynhG Score: 23.48 Experimentally tested	1755584	1756029	445	-	10.66	10.42	0.00
IS011	Chen et al.	450965	451296	331	+	cyoA/ampG tpe43 451113	450922	451057	135	+	10.49	10.46	0.00
IS011	Chen et al.	450965	451296	331	+	cyoA/ampG tpe43 451113	451094	451164	70	+	10.39	10.34	0.00
IS011	Chen et al.	450965	451296	331	+	cyoA/ampG tpe43 451113	451179	451247	68	+	10.04	10.1	0.00
C0887	Tjaden et al.	3782745	3782849	104	+	yibN/yibO	3782497	3783241	744	+	10.15	10	0.00
HB_446	Carter et al.	3044068	3044147	79	-	ygfF/gcvP tpe85 3044127 HB_447 3044028-3044107 -	3043139	3047963	4824	-	11.32	11.37	0.00
IS112	Chen et al.	2288224	2288371	147	-	yejO/narP	2288244	2288290	46	-	10.13	9.99	0.00
IS112	Chen et al.	2288224	2288371	147	-	yejO/narP	2288353	2288406	53	-	9.92	9.63	0.01
HB_441	Carter et al.	2902209	2902288	79	-	ygcE/ygcF	2902022	2902448	426	-	11.51	11.06	0.00
p8	Rivas et al.	3494525	?	?	unknown	nirD/nirC Score: 13.12	3491965	3494924	2959	+	9.99	10.14	0.00
C0888	Tjaden et al.	3785732	3785805	73	-	Not intergenic	3783401	3786316	2915	-	10.01	9.96	0.00
C0599	Tjaden et al.	2590810	2590906	96	-	dapE/ypfH	2590806	2590944	138	-	10.04	9.75	0.00
HB_544	Carter et al.	4368136	4368215	79	-	yjeH/groS	4366736	4368481	1745	-	10.55	10.24	0.00
HB_545	Carter et al.	4465069	4465148	79	-	treR/mgtA	4464681	4465384	703	-	9.95	9.67	0.00
HB_546	Carter et al.	4464989	4465068	79	-	treR/mgtA t34 4465014	4464681	4465384	703	-	9.95	9.67	0.00
HB_547	Carter et al.	4498658	4498737	79	-	yjgX/yjgZ	4498020	4499072	1052	-	10.6	10.32	0.00
HB_540	Carter et al.	4244193	4244272	79	-	malE/malK HB_539 4244233-4244312 -	4241034	4244311	3277	-	10.02	10.13	0.00
HB_443	Carter et al.	2969156	2969202	46	-	ygdQ/ygdR t77 2969166	2968404	2969246	842	-	9.88	9.72	0.00
HB_542	Carter et al.	4327950	4328029	79	-	yjcZ/proP	4327856	4328314	458	-	10.08	9.7	0.01
HB_543	Carter et al.	4339279	4339358	79	-	melR/melA tp9 4339324 C1010 4339207-4339284 -	4339335	4339676	341	-	9.89	9.99	0.00
tpe62	Rivas et al.	3850413	?	?	unknown	ilvB/ivbL Score: 10.19	3849695	3851037	1342	+	9.73	9.63	0.00
tpe62	Rivas et al.	3850413	?	?	unknown	ilvB/ivbL Score: 10.19	3848827	3851045	2218	-	10.56	10.62	0.00
tpe60	Rivas et al.	2151623	?	?	unknown	yegL/yegM HB_126 2151601-2151680 + Score: 10.20	2151589	2151628	39	+	9.54	9.27	0.01
tpe60	Rivas et al.	2151623	?	?	unknown	yegL/yegM HB_126 2151601-2151680 + Score: 10.20	2151475	2151628	153	-	10.41	9.91	0.01
HB_548	Carter et al.	4498538	4498617	79	-	yjgX/yjgZ C1061 4498468-4498595 -	4498020	4499072	1052	-	10.6	10.32	0.00
HB_549	Carter et al.	4499311	4499390	79	-	yjgZ/yjhB HB_550 4499271-4499350 -	4499302	4499457	155	-	9.56	9.67	0.00
HB_469	Carter et al.	3332323	3332379	56	-	ispB/sfsB	3331797	3332746	949	-	9.64	9.48	0.00

HB_468	Carter et al.	3326392	3326433	41	-	greA/dacB HB_467 3326394-3326473 - Overlaps a 5' UTR	3324973	3327254	2281	-	12.09	11.88	0.00
HB_467	Carter et al.	3326394	3326473	79	-	greA/dacB HB_468 3326392-3326433 - Overlaps a 5' UTR	3324973	3327254	2281	-	12.09	11.88	0.00
HB_465	Carter et al.	3237214	3237293	79	-	ygjT/ygjU HB_464 3237254-3237333 - C0740 3237188-3237292 -	3236826	3237772	946	-	9.7	9.6	0.00
HB_464	Carter et al.	3237254	3237333	79	-	ygjT/ygjU HB_463 3237294-3237373 - HB_465 3237214-3237293 - C0740 3237188-3237292 -	3236826	3237772	946	-	9.7	9.6	0.00
HB_463	Carter et al.	3237294	3237373	79	-	ygjT/ygjU HB_464 3237254-3237333 -	3236826	3237772	946	-	9.7	9.6	0.00
HB_119	Carter et al.	2087241	2087320	79	+	Not intergenic HB_120 2087281-2087360 +	2087235	2087402	167	+	9.79	9.79	0.00
HB_40	Carter et al.	550676	550749	73	+	ybcF/purK	549847	552365	2518	+	10.15	10.01	0.00
HB_41	Carter et al.	573643	573722	79	+	ybcQ/nmpC IS018 573636-573747 +	573661	573703	42	+	9.53	9.39	0.01
HB_42	Carter et al.	578367	578406	39	+	ybcU/ybcV	578256	578930	674	+	10.17	10.02	0.00
HB_43	Carter et al.	585227	585306	79	+	ompT/envY	585198	585265	67	+	11.73	11.62	0.00
HB_43	Carter et al.	585227	585306	79	+	ompT/envY	585276	585424	148	+	10.59	10.33	0.01
HB_44	Carter et al.	606897	606976	79	+	ybdK/ybdY HB_45 606937-607016 +	606878	607029	151	+	10.45	10.17	0.00
HB_45	Carter et al.	606937	607016	79	+	ybdK/ybdY HB_44 606897-606976 +	606878	607029	151	+	10.45	10.17	0.00
HB_46	Carter et al.	607097	607176	79	+	Not intergenic C0132 607070-607166 +	607124	608681	1557	+	10.8	10.68	0.00
HB_47	Carter et al.	607177	607237	60	+	Not intergenic	607124	608681	1557	+	10.8	10.68	0.00
HB_48	Carter et al.	608401	608480	79	+	ybdY/entD	607124	608681	1557	+	10.8	10.68	0.00
HB_49	Carter et al.	608601	608680	79	+	ybdY/entD	607124	608681	1557	+	10.8	10.68	0.00
HB_448	Carter et al.	3048738	3048814	76	-	gcvT/visC IS149 3048693-3048953 - Overlaps a 5' UTR	3048107	3048832	725	-	12.19	12.38	0.00
HB_10	Carter et al.	183621	183700	79	+	yaeG/yaeH HB_11 183661-183708 +	182450	184094	1644	+	10.21	10.04	0.00
C0592	Tjaden et al.	2559205	2559301	96	-	yffL/yffM	2559227	2559305	78	-	10.27	10.09	0.00
C0593	Tjaden et al.	2561161	2561457	296	-	yffP/yffQ IS125 2561257-2561485 - HB_413 2561267-2561346 -	2561149	2561235	86	-	9.99	9.93	0.00
C0593	Tjaden et al.	2561161	2561457	296	-	yffP/yffQ IS125 2561257-2561485 - HB_413 2561267-2561346 -	2561236	2561381	145	-	9.98	9.94	0.00
C0593	Tjaden et al.	2561161	2561457	296	-	yffP/yffQ IS125 2561257-2561485 - HB_413 2561267-2561346 -	2561450	2561490	40	-	9.6	9.78	0.01
HB_113	Carter et al.	1906226	1906284	58	+	yobF/yebO	1906170	1906567	397	+	10.36	10.29	0.00
C0795	Tjaden et al.	3440125	3440240	115	+	rpsM/rpmJ	3437704	3440327	2623	+	10.58	10.53	0.00
tpke120	Rivas et al.	3998739	?	?	unknown	yigE/corA Score: 8.69 Overlaps a 5' UTR	3998532	3998796	264	+	10.23	9.77	0.01
tpke120	Rivas et al.	3998739	?	?	unknown	yigE/corA Score: 8.69 Overlaps a 5' UTR	3998719	3999031	312	-	10.12	9.99	0.00
tpke122	Rivas et al.	3719753	?	?	unknown	yiaZ/glyS HB_495 3719717-3719796 - Score: 7.50 Experimentally tested	3719626	3719805	179	-	9.59	9.45	0.00
tpke123	Rivas et al.	4175870	?	?	unknown	nusG/rplK Score: 6.66 Resides within an operon	4172793	4176370	3577	+	12.22	11.89	0.00
tpke123	Rivas et al.	4175870	?	?	unknown	nusG/rplK Score: 6.66 Resides within an operon	4175729	4176074	345	-	10.09	10.05	0.00
te8	Rivas et al.	4401885	?	?	unknown	hflC/yjeT Score: 11.59	4390925	4404098	13173	+	11.61	11.39	0.00
te8	Rivas et al.	4401885	?	?	unknown	hflC/yjeT Score: 11.59	4400829	4402585	1756	-	9.95	9.81	0.00
HB_389	Carter et al.	2230768	2230847	79	-	cdd/sanA C0513 2230771-2230854 - HB_390 2230749-2230807 -	2230672	2230859	187	-	9.54	9.33	0.00
HB_386	Carter et al.	2217632	2217711	79	-	yehZ/bglX	2217551	2220054	2503	-	10.44	10.35	0.00
te1	Rivas et al.	455657	?	?	unknown	tig/clpP Score: 32.63 Experimentally tested	453559	457964	4405	+	12.15	12.07	0.00
HB_384	Carter et al.	2163560	2163639	79	-	yegP/yegQ t49 2163581	2163542	2163655	113	-	9.88	9.67	0.00
HB_382	Carter et al.	2151401	2151480	79	-	yegL/yegM HB_381 2151441-2151520 -	2151475	2151628	153	-	10.41	9.91	0.01
te5	Rivas et al.	1918171	?	?	unknown	yebT/yebU Score: 12.95	1914257	1918207	3950	+	10.79	10.51	0.00
te5	Rivas et al.	1918171	?	?	unknown	yebT/yebU Score: 12.95	1917308	1918207	899	-	9.9	9.85	0.00
HB_380	Carter et al.	2151721	2151800	79	-	yegL/yegM HB_379 2151761-2151840 -	2151763	2151871	108	-	10.66	10.47	0.00
HB_381	Carter et al.	2151441	2151520	79	-	yegL/yegM HB_382 2151401-2151480 -	2151475	2151628	153	-	10.41	9.91	0.01
HB_143	Carter et al.	2590955	2591034	79	+	dapE/ypfH	2590272	2591090	818	+	11.02	10.95	0.00
HB_112	Carter et al.	1878103	1878144	41	+	yeaR/yeaS	1878085	1878195	110	+	9.78	9.54	0.01
HB_145	Carter et al.	2642354	2642433	79	+	yfgB/ndk	2642221	2642696	475	+	9.85	9.7	0.00
HB_146	Carter et al.	2660362	2660441	79	+	yfhP/yfhQ	2660285	2660752	467	+	11	10.73	0.00
HB_147	Carter et al.	2687577	2687656	79	+	yfhG/yfhK	2687541	2687923	382	+	9.72	9.58	0.00
HB_148	Carter et al.	2702174	2702253	79	+	rnc/lepB HB_149 2702214-2702293 +	2702182	2702301	119	+	9.81	9.71	0.00
HB_149	Carter et al.	2702214	2702293	79	+	rnc/lepB HB_148 2702174-2702253 +	2702182	2702301	119	+	9.81	9.71	0.00
HB_263	Carter et al.	4499158	4499237	79	+	yjgZ/yjhB HB_264 4499198-4499277 +	4499104	4499195	91	+	9.67	9.55	0.00
HB_262	Carter et al.	4495669	4495744	75	+	intB/yjgX	4495350	4496005	655	+	11.42	10.93	0.01
HB_267	Carter et al.	4516371	4516450	79	+	fecI/yjhU HB_268 4516411-4516490 +	4516333	4517332	999	+	11.76	10.93	0.01
HB_266	Carter et al.	4507123	4507202	79	+	yjhE/fecE	4507018	4507905	887	+	9.97	9.91	0.00
HB_35	Carter et al.	387911	387976	65	+	tauD/hemB HB_34 387871-387950 +	385216	387940	2724	+	9.82	9.52	0.00
HB_34	Carter et al.	387871	387950	79	+	tauD/hemB HB_35 387911-387976 +	385216	387940	2724	+	9.82	9.52	0.00
HB_37	Carter et al.	497044	497123	79	+	adk/hemH Overlaps a 3' UTR	496365	497188	823	+	12.71	12.53	0.00
HB_36	Carter et al.	431488	431535	47	+	tsx/yajI	431456	431535	79	+	9.83	9.85	0.00
HB_31	Carter et al.	312612	312691	79	+	ykgM/eaeH	312612	312656	44	+	9.22	9.04	0.01

HB_31	Carter et al.	312612	312691	79	+	ykgM/eaeH	312657	312746	89	+	10.01	9.9	0.00
HB_30	Carter et al.	311211	311285	74	+	ykgK/ykgL	311210	311315	105	+	10.07	9.82	0.00
HB_33	Carter et al.	379217	379292	75	+	yaiN/yaiO C0086 379197-379268 +	379196	379298	102	+	10.16	10.15	0.00
HB_32	Carter et al.	376696	376758	62	+	yaiL/yaiM	375100	378320	3220	+	10.56	10.33	0.00
t21	Rivas et al.	3472191	?	?	unknown	rpsL/yheL C0807 3472189-3472298 + Score: 12.28 Experimentally tested Resides within an operon	3472157	3472634	477	+	10.66	10.46	0.00
t21	Rivas et al.	3472191	?	?	unknown	rpsL/yheL C0807 3472189-3472298 + Score: 12.28 Experimentally tested Resides within an operon	3468097	3472677	4580	-	13.47	13.4	0.00
C1009	Tjaden et al.	4335553	4335728	175	+	adiY/adiA	4335129	4336028	899	+	10.04	9.95	0.00
t23	Rivas et al.	1158528	?	?	unknown	ptsG/fhuE Score: 11.81	1156937	1158560	1623	+	12.51	12.51	0.00
t23	Rivas et al.	1158528	?	?	unknown	ptsG/fhuE Score: 11.81	1157003	1160862	3859	-	10.82	10.93	0.00
t22	Rivas et al.	460468	?	?	unknown	lon/hupB Score: 12.20	459705	463044	3339	+	12.13	11.96	0.00
HB_39	Carter et al.	547732	547787	55	+	Not intergenic HB_38 547692-547771 +	547678	547850	172	+	10.34	10.28	0.00
t24	Rivas et al.	463535	?	?	unknown	ybaV/ybaW Score: 11.71	463141	463549	408	+	10.59	10.47	0.00
IS227	Chen et al.	4611908	4612011	103	+	yjjV/yjjW	4610408	4612921	2513	+	10.84	10.8	0.00
C0509	Tjaden et al.	2224433	2224527	94	+	yohD/yohF	2223798	2224896	1098	+	10.93	10.63	0.00
C0263	Tjaden et al.	1067392	1067559	167	-	Not intergenic	1067167	1067410	243	-	9.78	9.44	0.01
C0263	Tjaden et al.	1067392	1067559	167	-	Not intergenic	1067512	1067558	46	-	10.17	10.12	0.00
C0260	Tjaden et al.	1051340	1051510	170	+	Not intergenic	1051181	1051466	285	+	12.87	12.59	0.00
C0260	Tjaden et al.	1051340	1051510	170	+	Not intergenic	1051466	1051627	161	+	10.65	10.29	0.01
C0267	Tjaden et al.	1080196	1080293	97	-	putP/ycdN HB_324 1080160-1080239 -	1080120	1080506	386	-	11	10.74	0.00
C0265	Tjaden et al.	1078410	1078489	79	+	putA/putP	1078302	1078488	186	+	10.71	10.44	0.00
C0973	Tjaden et al.	4178555	4178804	249	-	rplL/rpoB HB_529 4178504-4178572 -	4178083	4178564	481	-	10.07	10.03	0.00
C0973	Tjaden et al.	4178555	4178804	249	-	rplL/rpoB HB_529 4178504-4178572 -	4178583	4179112	529	-	9.89	9.63	0.00
pke1	Rivas et al.	3154534	?	?	unknown	yqhD/yqhE Score: 10.95	3153356	3155502	2146	+	10.7	10.72	0.00
pke1	Rivas et al.	3154534	?	?	unknown	yqhD/yqhE Score: 10.95	3153384	3154634	1250	-	9.7	9.73	0.00
pke3	Rivas et al.	2922553	?	?	unknown	yqcC/syd Score: 6.77	2921943	2922616	673	-	13.22	13.01	0.00
C1036	Tjaden et al.	4432093	4432177	84	+	ytfH/cpdB	4432142	4433642	1500	+	10.05	9.71	0.00
C0851	Tjaden et al.	3645700	3645859	159	+	gor/arsR	3645689	3645732	43	+	10.71	9.56	0.04
C0851	Tjaden et al.	3645700	3645859	159	+	gor/arsR	3645742	3645803	61	+	11.3	10.41	0.03
e5	Rivas et al.	3048689	?	?	unknown	gcvT/visC Score: 10.14 Overlaps a 5' UTR	3048080	3048832	752	+	9.66	9.77	0.00
e5	Rivas et al.	3048689	?	?	unknown	gcvT/visC Score: 10.14 Overlaps a 5' UTR	3048107	3048832	725	-	12.19	12.38	0.00
C0149	Tjaden et al.	659535	659634	99	+	lipA/ybeF	659512	660476	964	+	10.53	10.19	0.00
C0857	Tjaden et al.	3655198	3655608	410	-	hdeD/yhiE IS182 3655606-3655816 - HB_483 3655198-3655265 -	3655188	3655406	218	-	10.34	10.04	0.01
C0857	Tjaden et al.	3655198	3655608	410	-	hdeD/yhiE IS182 3655606-3655816 - HB_483 3655198-3655265 -	3655467	3655622	155	-	10.86	10.15	0.02
IS207	Chen et al.	4233138	4233291	153	-	pgi/yjbE	4231898	4233563	1665	-	10.61	10.61	0.00
C0142	Tjaden et al.	631320	631487	167	-	Not intergenic HB_310 631252-631331 -	631240	631455	215	-	9.72	9.5	0.00
IS205	Chen et al.	4174754	4174918	164	+	tufB/secE	4172793	4176370	3577	+	12.22	11.89	0.00
C0140	Tjaden et al.	628936	629050	114	-	ybdB/cstA	627457	628975	1518	-	10.06	10.13	0.00
HB_410	Carter et al.	2526021	2526100	79	-	Not intergenic HB_411 2525981-2526060 -	2524927	2526131	1204	-	9.87	9.79	0.00
IS201	Chen et al.	4106137	4106321	184	-	pfkA/sbp	4105771	4106500	729	-	9.93	9.93	0.00
C0496	Tjaden et al.	2116472	2116688	216	-	wcaK/wzxC HB_378 2116477-2116541 - HB_377 2116502-2116581 - HB_376 2116622-2116701 -	2116464	2117857	1393	-	10.06	9.96	0.00
HB_315	Carter et al.	754150	754229	79	-	gltA/sdhC	754152	754245	93	-	11.06	10.71	0.01
HB_314	Carter et al.	685982	686061	79	-	gltJ/ybeJ Overlaps a 3' UTR	685939	688289	2350	-	10.53	10.3	0.00
HB_317	Carter et al.	835444	835523	79	-	ybiB/ybiC HB_318 835434-835483 -	835450	835539	89	-	9.89	9.67	0.00
HB_311	Carter et al.	638776	638855	79	-	ahpC/ahpF HB_312 638736-638815 -	638089	638842	753	-	10.22	9.97	0.00
HB_310	Carter et al.	631252	631331	79	-	cstA/ybdD C0142 631320-631487 -	631240	631455	215	-	9.72	9.5	0.00
HB_313	Carter et al.	638732	638775	43	-	ahpC/ahpF HB_312 638736-638815 -	638089	638842	753	-	10.22	9.97	0.00
HB_312	Carter et al.	638736	638815	79	-	ahpC/ahpF HB_311 638776-638855 - HB_313 638732-638775 -	638089	638842	753	-	10.22	9.97	0.00
e1	Rivas et al.	4437372	?	?	unknown	ytfK/ytfL Score: 16.84	4437361	4437551	190	+	10.66	10.32	0.00
e1	Rivas et al.	4437372	?	?	unknown	ytfK/ytfL Score: 16.84	4437150	4437458	308	+	10.46	10.11	0.00
HB_319	Carter et al.	931688	931767	79	-	trxB/lrp	931640	931751	111	-	10.79	10.74	0.00
HB_319	Carter et al.	931688	931767	79	-	trxB/lrp	931762	932040	278	-	10.29	10.38	0.00
HB_318	Carter et al.	835434	835483	49	-	ybiB/ybiC HB_317 835444-835523 -	835450	835539	89	-	9.89	9.67	0.00
C0536	Tjaden et al.	2378534	2378740	206	+	Not intergenic p26 2378544 HB_135 2378529-2378608 +	2377336	2378691	1355	+	10.05	9.86	0.00
C0536	Tjaden et al.	2378534	2378740	206	+	Not intergenic p26 2378544 HB_135 2378529-2378608 +	2378699	2379021	322	+	9.58	9.1	0.01
C0466	Tjaden et al.	1975204	1975282	78	+	motA/flhC	1975193	1975590	397	+	9.76	9.84	0.00

C0467	Tjaden et al.	1976435	1976516	81	+	flhD/yecG	1976313	1976540	227	+	10.97	10.6	0.00
HB_414	Carter et al.	2563421	2563500	79	-	yffS/eutA HB_415 2563381-2563460 -	2562756	2564328	1572	-	9.83	9.76	0.00
C0462	Tjaden et al.	1946657	1946772	115	+	ntpA/aspS	1946544	1946730	186	+	9.43	9.03	0.01
C0463	Tjaden et al.	1948618	1948806	188	-	aspS/yecD	1944734	1948847	4113	-	11.61	11.42	0.00
C1002	Tjaden et al.	4310762	4310865	103	+	rpiR/rpiB	4310587	4310922	335	+	9.43	9.17	0.01
C0543	Tjaden et al.	2403551	2403694	143	+	nuoA/lrhA IS115 2403317-2403693 +	2403461	2404171	710	+	11.47	11.2	0.00
C0469	Tjaden et al.	1984828	1984900	72	+	araF/yecI	1984828	1984916	88	+	10.96	11.24	0.01
HB_449	Carter et al.	3054958	3055037	79	-	ygfA/serA	3054960	3055039	79	-	10.14	10.05	0.00
C0724	Tjaden et al.	3154691	3154740	49	-	Not intergenic	3154724	3154761	37	-	9.63	9.68	0.00
C0086	Tjaden et al.	379197	379268	71	+	yaiN/yaiO HB_33 379217-379292 +	379196	379298	102	+	10.16	10.15	0.00
C0087	Tjaden et al.	395627	395801	174	+	ampH/sbmA	395597	395829	232	+	11.69	11.33	0.00
C0085	Tjaden et al.	374105	374609	504	-	mhpE/mhpT	374118	375211	1093	-	10.03	9.84	0.00
C0083	Tjaden et al.	353936	354076	140	-	prpE/codB	353660	354045	385	-	10.48	10.52	0.00
IS052	Chen et al.	1216232	1216376	144	-	ymgC/ycgG	1216335	1216382	47	-	10.57	10.54	0.00
IS050	Chen et al.	1195609	1195836	227	-	icd/ymfD C0292 1195705-1195804 -	1195683	1195773	90	-	9.45	9.17	0.01
C0649	Tjaden et al.	2769734	2769823	89	+	yfjT/yfjU	2769774	2770842	1068	+	10.16	9.99	0.00
IS054	Chen et al.	1223125	1223269	144	-	ycgI/minE IS055 1223195-1223288 -	1223189	1223240	51	-	9.95	9.75	0.01
IS055	Chen et al.	1223195	1223288	93	-	ycgI/minE IS054 1223125-1223269 -	1223189	1223240	51	-	9.95	9.75	0.01
HB_302	Carter et al.	421584	421648	64	-	proY/malZ HB_301 421609-421688 -	421504	421617	113	-	9.3	9.29	0.00
HB_302	Carter et al.	421584	421648	64	-	proY/malZ HB_301 421609-421688 -	421629	421679	50	-	10.3	10.27	0.00
C0314	Tjaden et al.	1286562	1286744	182	+	tyrV/tyrT	1286741	1286930	189	+	9.92	9.79	0.00
IS059	Chen et al.	1342431	1342640	209	-	yciT/yciR Overlaps a 5' UTR	1342397	1342714	317	-	11.9	11.88	0.00
C0313	Tjaden et al.	1285808	1285883	75	+	narI/tpr HB_83 1285830-1285881 + HB_82 1285790-1285869 +	1285784	1285833	49	+	10.68	10.54	0.00
C0313	Tjaden et al.	1285808	1285883	75	+	narI/tpr HB_83 1285830-1285881 + HB_82 1285790-1285869 +	1285843	1285906	63	+	9.85	9.87	0.00
C0643	Tjaden et al.	2744245	2744315	70	+	rpsP/ffh HB_151 2744296-2744375 +	2743738	2744272	534	+	9.74	9.75	0.00
C0643	Tjaden et al.	2744245	2744315	70	+	rpsP/ffh HB_151 2744296-2744375 +	2744272	2744316	44	+	10.09	9.78	0.01
tp55	Rivas et al.	450836	?	?	unknown	cyoA/ampG Score: 5.16	450836	450914	78	+	10.8	10.81	0.00
HB_61	Carter et al.	779853	779932	79	+	lysT/valT HB_62 779893-779937 +	773944	779900	5956	+	12.09	11.86	0.00
tp53	Rivas et al.	3597348	?	?	unknown	livJ/rpoH Score: 5.57	3596855	3597568	713	+	9.89	9.3	0.01
tp53	Rivas et al.	3597348	?	?	unknown	livJ/rpoH Score: 5.57	3596578	3597714	1136	-	10.36	10.27	0.00
tp51	Rivas et al.	201998	?	?	unknown	lpxD/fabZ Score: 5.83 Resides within an operon	195136	209614	14478	+	11.88	11.73	0.00
tp51	Rivas et al.	201998	?	?	unknown	lpxD/fabZ Score: 5.83 Resides within an operon	201751	202075	324	-	9.99	9.85	0.00
tp50	Rivas et al.	3246466	?	?	unknown	yqjB/yqjC Score: 6.36	3245631	3246898	1267	+	11.67	11.36	0.00
tp50	Rivas et al.	3246466	?	?	unknown	yqjB/yqjC Score: 6.36	3245631	3246688	1057	-	10.01	9.82	0.00
C0635	Tjaden et al.	2729395	2729560	165	-	rrsG/clpB t55 2729424	2727325	2729521	2196	-	11.5	10	0.04
C0635	Tjaden et al.	2729395	2729560	165	-	rrsG/clpB t55 2729424	2729524	2732344	2820	-	11.75	11.41	0.00
IS110	Chen et al.	2267589	2267948	359	-	yeiU/spr	2267254	2267614	360	-	9.79	9.6	0.00
IS110	Chen et al.	2267589	2267948	359	-	yeiU/spr	2267635	2267707	72	-	10.29	9.72	0.01
IS110	Chen et al.	2267589	2267948	359	-	yeiU/spr	2267722	2267773	51	-	10.77	10.27	0.01
IS110	Chen et al.	2267589	2267948	359	-	yeiU/spr	2267777	2267870	93	-	10.26	10.1	0.00
IS110	Chen et al.	2267589	2267948	359	-	yeiU/spr	2267903	2267958	55	-	9.37	9.52	0.00
IS117	Chen et al.	2460675	2460836	161	-	fadL/yfdF HB_409 2460667-2460741 -	2459360	2460700	1340	-	9.57	9.46	0.00
IS117	Chen et al.	2460675	2460836	161	-	fadL/yfdF HB_409 2460667-2460741 -	2460774	2460829	55	-	9.48	9.27	0.01
IS117	Chen et al.	2460675	2460836	161	-	fadL/yfdF HB_409 2460667-2460741 -	2460833	2460875	42	-	10.57	10.6	0.00
IS116	Chen et al.	2411264	2411416	152	+	yfbV/ackA Overlaps a 5' UTR	2411266	2414837	3571	+	12.09	11.71	0.00
IS115	Chen et al.	2403317	2403693	376	+	nuoA/lrhA k30 2403505 C0543 2403551-2403694 +	2403360	2403460	100	+	10.02	9.45	0.01
IS115	Chen et al.	2403317	2403693	376	+	nuoA/lrhA k30 2403505 C0543 2403551-2403694 +	2403461	2404171	710	+	11.47	11.2	0.00
IS114	Chen et al.	2311236	2311393	157	-	ompC/yojN	2311146	2311349	203	-	10.61	10.44	0.00
IS114	Chen et al.	2311236	2311393	157	-	ompC/yojN	2311363	2311402	39	-	9.67	9.42	0.01
C0792	Tjaden et al.	3436196	3436292	96	+	Not intergenic	3435985	3436558	573	+	11.68	11.58	0.00
tpe95	Rivas et al.	1269546	?	?	unknown	kdsA/chaA Score: 6.04 Overlaps a 3' UTR	1262830	1269867	7037	+	11.69	11.38	0.00
tpe95	Rivas et al.	1269546	?	?	unknown	kdsA/chaA Score: 6.04 Overlaps a 3' UTR	1268279	1269894	1615	-	10.22	10.03	0.01
HB_177	Carter et al.	3154573	3154652	79	+	yqhD/yqhE	3153356	3155502	2146	+	10.7	10.72	0.00
C1099	Tjaden et al.	4632964	4633003	39	+	rob/creA	4632720	4633421	701	+	10.28	10.23	0.00
HB_423	Carter et al.	2765435	2765514	79	-	yfjO/yfjP	2764035	2765595	1560	-	10.56	10.26	0.00
pk2	Rivas et al.	1798025	?	?	unknown	rpmI/infC C0421 1798023-1798118 + Score: 12.18<br>Resides within an operon	1797832	1800643	2811	+	10.09	9.96	0.00

pk2	Rivas et al.	1798025	?	?	unknown	rpmI/infC C0421 1798023-1798118 + Score: 12.18<br>Resides within an operon	1797832	1800774	2942	-	13.76	13.46	0.00
HB_421	Carter et al.	2751645	2751724	79	-	Not intergenic HB_420 2751685-2751764 -	2751661	2751792	131	-	9.83	9.78	0.00
HB_420	Carter et al.	2751685	2751764	79	-	Not intergenic HB_421 2751645-2751724 -	2751661	2751792	131	-	9.83	9.78	0.00
HB_427	Carter et al.	2773770	2773849	79	-	Not intergenic	2773684	2773904	220	-	9.8	9.61	0.00
HB_426	Carter et al.	2771169	2771248	79	-	Not intergenic	2770309	2771302	993	-	10.14	10.21	0.00
HB_425	Carter et al.	2769637	2769700	63	-	yfjT/yfjU HB_424 2769661-2769740 -	2767480	2769798	2318	-	9.87	9.71	0.00
HB_424	Carter et al.	2769661	2769740	79	-	yfjT/yfjU HB_425 2769637-2769700 -	2767480	2769798	2318	-	9.87	9.71	0.00
C0634	Tjaden et al.	2723788	2724007	219	+	kgtP/rrfG HB_150 2723937-2724016 +	2722986	2723829	843	+	10.32	10	0.00
C0634	Tjaden et al.	2723788	2724007	219	+	kgtP/rrfG HB_150 2723937-2724016 +	2723859	2724022	163	+	11.75	11.09	0.01
IS125	Chen et al.	2561257	2561485	228	-	yffP/yffQ HB_413 2561267-2561346 - C0593 2561161-2561457 -	2561236	2561381	145	-	9.98	9.94	0.00
IS125	Chen et al.	2561257	2561485	228	-	yffP/yffQ HB_413 2561267-2561346 - C0593 2561161-2561457 -	2561450	2561490	40	-	9.6	9.78	0.01
HB_429	Carter et al.	2815686	2815765	79	-	yqaB/argQ	2815658	2816758	1100	-	13.73	13.37	0.00
HB_428	Carter et al.	2798534	2798613	79	-	ygaM/nrdH	2798205	2798726	521	-	11.57	11.1	0.00
HB_84	Carter et al.	1299109	1299155	46	+	ychE/oppA Overlaps a 5' UTR	1298977	1304834	5857	+	12.24	11.96	0.00
HB_85	Carter et al.	1306800	1306879	79	+	cls/kch	1306726	1306978	252	+	12.92	12.48	0.00
HB_86	Carter et al.	1328903	1328982	79	+	yciN/topA HB_87 1328943-1329021 + Overlaps a 5' UTR	1328810	1328943	133	+	11.08	10.48	0.01
HB_86	Carter et al.	1328903	1328982	79	+	yciN/topA HB_87 1328943-1329021 + Overlaps a 5' UTR	1328946	1331281	2335	+	12	11.74	0.00
HB_87	Carter et al.	1328943	1329021	78	+	yciN/topA HB_86 1328903-1328982 + Overlaps a 5' UTR	1328946	1331281	2335	+	12	11.74	0.00
HB_80	Carter et al.	1226232	1226293	61	+	ycgJ/ycgK	1226208	1226694	486	+	10.69	10.52	0.00
HB_81	Carter et al.	1239173	1239252	79	+	dadX/ycgO	1238292	1239230	938	+	10.24	10.09	0.00
HB_81	Carter et al.	1239173	1239252	79	+	dadX/ycgO	1239249	1239290	41	+	9.74	9.44	0.01
HB_82	Carter et al.	1285790	1285869	79	+	narI/tpr C0313 1285808-1285883 + HB_83 1285830-1285881 +	1285784	1285833	49	+	10.68	10.54	0.00
HB_82	Carter et al.	1285790	1285869	79	+	narI/tpr C0313 1285808-1285883 + HB_83 1285830-1285881 +	1285843	1285906	63	+	9.85	9.87	0.00
HB_83	Carter et al.	1285830	1285881	51	+	narI/tpr C0313 1285808-1285883 + HB_82 1285790-1285869 +	1285784	1285833	49	+	10.68	10.54	0.00
HB_83	Carter et al.	1285830	1285881	51	+	narI/tpr C0313 1285808-1285883 + HB_82 1285790-1285869 +	1285843	1285906	63	+	9.85	9.87	0.00
HB_88	Carter et al.	1333254	1333333	79	+	Not intergenic HB_89 1333294-1333373 +	1333131	1333509	378	+	11.11	10.84	0.00
HB_89	Carter et al.	1333294	1333373	79	+	Not intergenic HB_88 1333254-1333333 +	1333131	1333509	378	+	11.11	10.84	0.00
C1087	Tjaden et al.	4603371	4603606	235	-	Not intergenic	4602473	4603714	1241	-	14.2	13.89	0.00
C0448	Tjaden et al.	1927890	1928002	112	+	yebE/yebF	1927881	1928168	287	+	10.19	10.14	0.00
C0976	Tjaden et al.	4188185	4188292	107	+	htrC/thiH	4187719	4188388	669	+	10.75	10.44	0.00
psrA6	Argaman et al.	1860608	1860782	174	-	yeaA/gapA C0430 1860674-1860747 -	1860678	1860728	50	-	9.62	9.59	0.00
C0651	Tjaden et al.	2775855	2776034	179	+	ypjF/ypjA	2775848	2775909	61	+	10.15	10.24	0.00
C0651	Tjaden et al.	2775855	2776034	179	+	ypjF/ypjA	2775914	2775954	40	+	9.87	9.8	0.00
C0651	Tjaden et al.	2775855	2776034	179	+	ypjF/ypjA	2775996	2776124	128	+	9.58	9.62	0.00
C0970	Tjaden et al.	4177248	4177547	299	-	rplA/rplJ HB_528 4177364-4177443 -	4176522	4178018	1496	-	10.27	10.06	0.00
C0447	Tjaden et al.	1923381	1923459	78	+	holE/yobB	1923386	1923708	322	+	11.1	11.07	0.00
IS186	Chen et al.	3719854	3719951	97	+	yiaZ/glyS	3719781	3719975	194	+	9.72	9.15	0.02
IS180	Chen et al.	3596063	3596191	128	+	yhhK/livJ	3595908	3596633	725	+	10.4	10.29	0.00
IS181	Chen et al.	3633531	3633749	218	-	yhiM/yhiN	3632732	3633588	856	-	9.63	9.58	0.00
IS181	Chen et al.	3633531	3633749	218	-	yhiM/yhiN	3633687	3633995	308	-	10.17	9.82	0.01
IS182	Chen et al.	3655606	3655816	210	-	hdeD/yhiE C0857 3655198-3655608 -	3655467	3655622	155	-	10.86	10.15	0.02
IS182	Chen et al.	3655606	3655816	210	-	hdeD/yhiE C0857 3655198-3655608 -	3655774	3655873	99	-	10.72	10.49	0.00
te20	Rivas et al.	3208645	?	?	unknown	rpsU/dnaG Score: 7.94 Resides within an operon	3207242	3208675	1433	-	10.57	10.41	0.00
C1083	Tjaden et al.	4593357	4593440	83	-	yjjM/yjjN	4593402	4593693	291	-	9.32	9.52	0.01
IS167	Chen et al.	3407629	3407776	147	-	prmA/yhdG	3407503	3408052	549	-	10.03	9.87	0.00
psrA23	Argaman et al.	4098266	4098318	52	-	rhaT/sodA	4098299	4098592	293	-	9.81	9.62	0.00
tk11	Rivas et al.	2618182	?	?	unknown	uraA/upp Score: 10.47 Resides within an operon	2617278	2618226	948	-	9.94	9.67	0.00
tpe18	Rivas et al.	83537	?	?	unknown	leuA/leuL Score: 15.23 Resides within an operon	83160	83557	397	-	10.86	10.78	0.00
HB_518	Carter et al.	4005406	4005485	79	-	Not intergenic	4004536	4005555	1019	-	9.83	9.77	0.00
tk15	Rivas et al.	3151442	?	?	unknown	metC/yghB Score: 7.95 Resides within an operon	3149795	3152374	2579	+	10.64	10.32	0.00
tk15	Rivas et al.	3151442	?	?	unknown	metC/yghB Score: 7.95 Resides within an operon	3150676	3151481	805	-	10.49	10.33	0.00
tk16	Rivas et al.	892670	?	?	unknown	ybjN/potF Score: 6.35	891604	892735	1131	+	11.21	10.92	0.00
tk16	Rivas et al.	892670	?	?	unknown	ybjN/potF Score: 6.35	892468	892681	213	-	9.74	9.62	0.00
HB_104	Carter et al.	1647266	1647345	79	+	rzpQ/dicB Overlaps a 5' UTR	1647187	1647454	267	+	10.2	9.92	0.00
HB_105	Carter et al.	1762901	1762957	56	+	ydiC/ydiH	1762736	1763139	403	+	10.2	9.95	0.00
HB_106	Carter et al.	1766800	1766879	79	+	ydiJ/ydiK HB_107 1766840-1766919 +	1766770	1768304	1534	+	10.57	10.34	0.00
HB_107	Carter et al.	1766840	1766919	79	+	ydiJ/ydiK HB_106 1766800-1766879 +	1766770	1768304	1534	+	10.57	10.34	0.00

HB_100	Carter et al.	1620744	1620823	79	+	ydeE/ydeH	1620573	1620879	306	+	13.1	12.59	0.00
HB_101	Carter et al.	1642458	1642537	79	+	ydfU/rem	1642436	1642473	37	+	9.86	9.7	0.01
HB_101	Carter et al.	1642458	1642537	79	+	ydfU/rem	1642488	1642584	96	+	9.9	9.86	0.00
HB_102	Carter et al.	1643057	1643136	79	+	rem/relF	1643064	1643142	78	+	10.26	9.99	0.01
HB_103	Carter et al.	1647106	1647185	79	+	rzpQ/dicB	1647094	1647157	63	+	10.11	10.05	0.00
HB_517	Carter et al.	3998828	3998907	79	-	yigE/corA	3998719	3999031	312	-	10.12	9.99	0.00
HB_516	Carter et al.	3992162	3992241	79	-	Not intergenic	3989072	3992460	3388	-	10.57	10.45	0.00
HB_515	Carter et al.	3975362	3975432	70	-	Not intergenic	3974118	3975888	1770	-	9.76	9.39	0.01
HB_108	Carter et al.	1803218	1803297	79	+	arpB/ydiY C0424 1803239-1803347 +	1803160	1803472	312	+	11.25	11.01	0.00
HB_458	Carter et al.	3220091	3220147	56	-	ebgR/ebgA	3219774	3220219	445	-	10.97	10.77	0.01
HB_459	Carter et al.	3229176	3229255	79	-	ygjK/fadH C0736 3229118-3229257 -	3229157	3229664	507	-	9.87	9.82	0.00
HB_269	Carter et al.	4516451	4516530	79	+	fecI/yjhU HB_270 4516491-4516570 + HB_268 4516411-4516490 +	4516333	4517332	999	+	11.76	10.93	0.01
HB_230	Carter et al.	4163945	4164024	79	+	murI/rrsB	4161552	4164343	2791	+	10.94	10.79	0.00
HB_365	Carter et al.	1797050	1797129	79	-	pheS/pheM HB_364 1797090-1797169 - Resides within an operon	1790236	1797387	7151	-	11.17	11.04	0.00
tpke79	Rivas et al.	1921027	?	?	unknown	pphA/yebY Score: 11.82	1920072	1921029	957	+	10.89	10.48	0.00
HB_79	Carter et al.	1223171	1223250	79	+	ycgI/minE	1223156	1223248	92	+	10.08	10.01	0.00
HB_79	Carter et al.	1223171	1223250	79	+	ycgI/minE	1223248	1223288	40	+	9.72	9.53	0.01
psrA17	Argaman et al.	3474078	3474144	66	-	yheO/fkpA tpk9 3474091 Resides within an operon	3472755	3475556	2801	-	11.55	11.31	0.00
HB_367	Carter et al.	1820352	1820431	79	-	osmE/nadE	1820383	1820444	61	-	10.04	9.96	0.00
psrA13	Argaman et al.	3079665	3079899	234	+	tktA/yggG	3079666	3079764	98	+	9.32	9.06	0.01
psrA13	Argaman et al.	3079665	3079899	234	+	tktA/yggG	3079867	3080174	307	+	10.38	10.41	0.00
HB_71	Carter et al.	910323	910402	79	+	poxB/ybjV	910307	910359	52	+	10.2	10.16	0.00
HB_70	Carter et al.	858375	858435	60	+	ybiU/ybiV	858391	858545	154	+	10.26	10.38	0.00
HB_73	Carter et al.	914299	914378	79	+	ybjE/aqpZ HB_72 914259-914338 +	914223	914521	298	+	10.64	10.31	0.01
HB_72	Carter et al.	914259	914338	79	+	ybjE/aqpZ HB_73 914299-914378 +	914223	914521	298	+	10.64	10.31	0.01
HB_75	Carter et al.	1031266	1031311	45	+	serT/hyaA Overlaps a 5' UTR	1031203	1031581	378	+	11.29	11.01	0.00
HB_74	Carter et al.	924764	924843	79	+	clpA/serW	921524	924793	3269	+	11.69	11.65	0.00
HB_77	Carter et al.	1201334	1201413	79	+	ymfI/ymfK	1200723	1201412	689	+	11.23	11	0.00
HB_76	Carter et al.	1039736	1039789	53	+	Not intergenic Resides within an operon	1037076	1041117	4041	+	10.6	10.32	0.00
psrA22	Argaman et al.	4048823	4048916	93	-	yihA/yihI	4047986	4048973	987	-	10.83	10.68	0.00
C0459	Tjaden et al.	1942229	1942307	78	-	znuB/ruvB	1941674	1944061	2387	-	10.81	10.89	0.00
HB_271	Carter et al.	4516531	4516610	79	+	fecI/yjhU HB_272 4516571-4516650 + HB_270 4516491-4516570 +	4516333	4517332	999	+	11.76	10.93	0.01
C0456	Tjaden et al.	1938259	1938335	76	+	msbB/yebA	1937555	1938463	908	+	9.91	9.77	0.00
C0102	Tjaden et al.	454271	454319	48	-	bolA/tig	454265	455347	1082	-	10.09	10.15	0.00
HB_118	Carter et al.	2078664	2078743	79	+	yeeA/sbmC	2078683	2078753	70	+	10.1	9.94	0.00
cc4_13	Rivas et al.	3948045	?	?	unknown	ilvL/ilvG HB_512 3948044-3948092 - Score: 9.68 Experimentally tested Resides within an operon	3947921	3948183	262	+	10.19	9.99	0.00
HB_511	Carter et al.	3906057	3906136	79	-	pstB/pstA	3904877	3906416	1539	-	10.63	10.62	0.00
HB_238	Carter et al.	4228846	4228925	79	+	yjbC/yjbD HB_237 4228806-4228885 +	4228413	4228872	459	+	10.7	10.67	0.00
IS174	Chen et al.	3537428	3537623	195	-	yhgF/feoA	3537375	3537456	81	-	9.7	9.61	0.00
IS174	Chen et al.	3537428	3537623	195	-	yhgF/feoA	3537524	3537661	137	-	10.65	10.47	0.00
HB_239	Carter et al.	4237430	4237509	79	+	yjbH/yjbA HB_240 4237470-4237549 +	4237198	4237545	347	+	10.08	9.97	0.00
HB_255	Carter et al.	4349936	4350015	79	+	yjdJ/yjdK	4349720	4350517	797	+	11.57	11.27	0.00
HB_359	Carter et al.	1627063	1627108	45	-	ydfH/ydfZ	1626471	1627114	643	-	10.26	10.08	0.00
HB_358	Carter et al.	1501191	1501270	79	-	Not intergenic	1501169	1501248	79	-	9.91	9.57	0.01
HB_212	Carter et al.	3957107	3957149	42	+	ilvC/ppiC	3955937	3957528	1591	+	10.07	10.19	0.00
HB_213	Carter et al.	3963706	3963785	79	+	trxA/rho	3962971	3964181	1210	+	12.28	11.9	0.00
HB_214	Carter et al.	3984231	3984298	67	+	aslA/hemY	3984194	3984277	83	+	10	9.48	0.01
HB_215	Carter et al.	3988451	3988530	79	+	hemC/cyaA IS193 3988483-3988583 + Overlaps a 5' UTR	3988268	3991869	3601	+	12.49	12.18	0.00
HB_216	Carter et al.	4005462	4005541	79	+	Not intergenic	4003842	4006585	2743	+	10.79	10.53	0.00
HB_217	Carter et al.	4008434	4008513	79	+	Not intergenic	4007121	4008969	1848	+	11.17	11.01	0.00
HB_351	Carter et al.	1349311	1349390	79	-	fabI/ycjD HB_350 1349351-1349430 -	1349170	1349357	187	-	10.14	10.01	0.00
HB_351	Carter et al.	1349311	1349390	79	-	fabI/ycjD HB_350 1349351-1349430 -	1349351	1349739	388	-	10.83	10.53	0.00
HB_350	Carter et al.	1349351	1349430	79	-	fabI/ycjD HB_351 1349311-1349390 -	1349351	1349739	388	-	10.83	10.53	0.00
HB_353	Carter et al.	1357434	1357513	79	-	ycjJ/ycjK C0337 1357334-1357442 -	1357397	1357501	104	-	10.4	10	0.01
HB_352	Carter et al.	1349231	1349310	79	-	fabI/ycjD Overlaps a 5' UTR	1349170	1349357	187	-	10.14	10.01	0.00
HB_355	Carter et al.	1488680	1488759	79	-	Not intergenic Overlaps a 5' UTR	1488348	1488828	480	-	14.06	13.79	0.00
HB_354	Carter et al.	1407365	1407444	79	-	ydaN/dbpA	1407362	1407511	149	-	10.54	10.12	0.01

HB_357	Carter et al.	1490204	1490243	39	-	ydcB/trg	1489876	1490261	385	-	11.31	11.26	0.00
HB_356	Carter et al.	1489906	1489985	79	-	Not intergenic C0352 1489877-1489979 -	1489876	1490261	385	-	11.31	11.26	0.00
t43	Rivas et al.	4119869	?	?	unknown	hslV/ftsN Score: 8.05	4119153	4121307	2154	+	9.99	9.86	0.00
t43	Rivas et al.	4119869	?	?	unknown	hslV/ftsN Score: 8.05	4118536	4120377	1841	-	11.61	11.26	0.00
t71	Rivas et al.	2809324	?	?	unknown	mprA/emrA Score: 5.55	2807594	2812104	4510	+	11.24	11.01	0.00
t71	Rivas et al.	2809324	?	?	unknown	mprA/emrA Score: 5.55	2808979	2809349	370	-	10.43	10.15	0.00
t77	Rivas et al.	2969166	?	?	unknown	ygdQ/ygdR HB_443 2969156-2969202 - Score: 5.05	2967736	2969246	1510	+	10.55	10.13	0.00
t77	Rivas et al.	2969166	?	?	unknown	ygdQ/ygdR HB_443 2969156-2969202 - Score: 5.05	2968404	2969246	842	-	9.88	9.72	0.00
t74	Rivas et al.	1152418	?	?	unknown	fabF/pabC Score: 5.17	1145826	1156894	11068	+	12.48	12.28	0.00
t75	Rivas et al.	3303464	?	?	unknown	mtr/deaD Score: 5.14	3302187	3303887	1700	+	10.31	10.51	0.00
t75	Rivas et al.	3303464	?	?	unknown	mtr/deaD Score: 5.14	3302272	3303887	1615	-	12.59	12.44	0.00
C0420	Tjaden et al.	1796981	1797146	165	+	pheS/pheM	1796221	1797024	803	+	10.13	10.08	0.00
C0420	Tjaden et al.	1796981	1797146	165	+	pheS/pheM	1797055	1797162	107	+	9.92	9.81	0.00
C0421	Tjaden et al.	1798023	1798118	95	+	rpmI/infC pk2 1798025	1797832	1800643	2811	+	10.09	9.96	0.00
HB_256	Carter et al.	4350659	4350738	79	+	yjdO/lysU	4350532	4350944	412	+	11.55	11.44	0.00
C0424	Tjaden et al.	1803239	1803347	108	+	arpB/ydiY HB_108 1803218-1803297 +	1803160	1803472	312	+	11.25	11.01	0.00
C0425	Tjaden et al.	1804173	1804380	207	+	ydiY/pfkB	1804174	1804230	56	+	9.45	9.23	0.01
C0425	Tjaden et al.	1804173	1804380	207	+	ydiY/pfkB	1804248	1804286	38	+	9.42	9.35	0.00
C0425	Tjaden et al.	1804173	1804380	207	+	ydiY/pfkB	1804345	1805398	1053	+	10.37	10.29	0.00
IS189	Chen et al.	3790319	3790421	102	+	kbl/htrL	3790097	3790371	274	+	9.51	9.61	0.00
C0352	Tjaden et al.	1489877	1489979	102	-	Not intergenic HB_356 1489906-1489985 -	1489876	1490261	385	-	11.31	11.26	0.00
C0350	Tjaden et al.	1488549	1488713	164	+	Not intergenic	1488539	1488757	218	+	9.88	9.92	0.00
C0358	Tjaden et al.	1522401	1522473	72	-	yncE/ansP	1521296	1523861	2565	-	10.38	10.34	0.00
HB_519	Carter et al.	4006513	4006565	52	-	Not intergenic	4005753	4007112	1359	-	10.24	10.09	0.00
p11	Rivas et al.	3309327	?	?	unknown	rpsO/truB Score: 11.55 Overlaps a 5' UTR	3306790	3309384	2594	+	9.99	9.85	0.00
p11	Rivas et al.	3309327	?	?	unknown	rpsO/truB Score: 11.55 Overlaps a 5' UTR	3306073	3309331	3258	-	12.52	12.2	0.00
p12	Rivas et al.	711192	?	?	unknown	ybfE/ybfF Score: 11.06	710712	714479	3767	+	10.7	10.63	0.00
p12	Rivas et al.	711192	?	?	unknown	ybfE/ybfF Score: 11.06	710828	712091	1263	-	10.6	10.41	0.00
p13	Rivas et al.	1268489	?	?	unknown	kdsA/chaA Score: 9.59 Overlaps a 3' UTR	1262830	1269867	7037	+	11.69	11.38	0.00
p13	Rivas et al.	1268489	?	?	unknown	kdsA/chaA Score: 9.59 Overlaps a 3' UTR	1268279	1269894	1615	-	10.22	10.03	0.01
C0194	Tjaden et al.	836732	836807	75	+	ybiC/ybiJ HB_68 836740-836819 +	835541	836780	1239	+	10.75	10.76	0.00
p15	Rivas et al.	4381201	?	?	unknown	yjeA/yjeM Score: 9.38	4380536	4381695	1159	+	10.81	10.4	0.00
p16	Rivas et al.	3208226	?	?	unknown	ygjD/rpsU Score: 8.91	3207332	3208337	1005	+	10.49	10.44	0.00
p16	Rivas et al.	3208226	?	?	unknown	ygjD/rpsU Score: 8.91	3207242	3208675	1433	-	10.57	10.41	0.00
C0197	Tjaden et al.	846352	846479	127	+	glnP/glnH HB_69 846393-846472 +	845772	846375	603	+	9.65	9.71	0.00
C0197	Tjaden et al.	846352	846479	127	+	glnP/glnH HB_69 846393-846472 +	846381	846703	322	+	10.33	10.22	0.00
C0198	Tjaden et al.	847276	847423	147	-	glnH/dps	842340	847421	5081	-	10.52	10.3	0.00
p19	Rivas et al.	3274496	?	?	unknown	yhaG/sohA Score: 8.20	3274262	3274582	320	+	9.66	9.71	0.00
IS154	Chen et al.	3181333	3181587	254	-	ygiE/ribB HB_455 3181349-3181428 -	3180827	3181641	814	-	11.63	11.33	0.00
IS153	Chen et al.	3161571	3161703	132	+	plsC/parC	3161638	3161734	96	+	9.87	9.91	0.00
IS152	Chen et al.	3119392	3119548	156	+	yghK/glcB	3119393	3120194	801	+	10.15	9.98	0.00
C0215	Tjaden et al.	924883	924970	87	+	clpA/serW	924862	924929	67	+	11.11	10.69	0.01
IS150	Chen et al.	3054867	3055018	151	+	ygfA/serA	3054863	3056476	1613	+	11	10.71	0.00
HB_254	Carter et al.	4339257	4339336	79	+	melR/melA tp9 4339324	4339258	4339315	57	+	10.61	10.65	0.00
tp19	Rivas et al.	2410635	?	?	unknown	yfbU/yfbV Score: 13.64	2409478	2411394	1916	-	11.92	11.77	0.00
tp18	Rivas et al.	1910602	?	?	unknown	htpX/prc Score: 13.83	1909735	1910658	923	-	11.62	11.52	0.00
k48	Rivas et al.	764274	?	?	unknown	sucD/farR Score: 5.72	757428	764347	6919	+	10.71	10.68	0.00
k49	Rivas et al.	1227965	?	?	unknown	ycgM/ycgN Score: 5.46	1227220	1228469	1249	+	10.78	10.79	0.00
tp13	Rivas et al.	1793178	?	?	unknown	btuC/ihfA Score: 15.52	1792758	1793217	459	+	9.76	9.71	0.00
tp13	Rivas et al.	1793178	?	?	unknown	btuC/ihfA Score: 15.52	1790236	1797387	7151	-	11.17	11.04	0.00
k45	Rivas et al.	938568	?	?	unknown	ycaJ/serS Score: 6.34 Resides within an operon	931590	939799	8209	+	12.17	12.15	0.00
k46	Rivas et al.	1932701	?	?	unknown	edd/zwf Score: 5.96	1932683	1932731	48	+	9.77	9.71	0.00
k46	Rivas et al.	1932701	?	?	unknown	edd/zwf Score: 5.96	1931956	1932787	831	-	10.25	9.95	0.00
C0294	Tjaden et al.	1223306	1223396	90	-	ycgI/minE	1223305	1223361	56	-	10.44	9.6	0.03
C0294	Tjaden et al.	1223306	1223396	90	-	ycgI/minE	1223395	1223434	39	-	10.47	10.21	0.01
k40	Rivas et al.	3577279	?	?	unknown	yhhW/yhhX Score: 7.09 Resides within an operon	3577227	3577283	56	+	9.86	9.81	0.00
k40	Rivas et al.	3577279	?	?	unknown	yhhW/yhhX Score: 7.09 Resides within an operon	3576922	3577320	398	-	10.44	10.21	0.00
k41	Rivas et al.	1944003	?	?	unknown	ruvA/yebB Score: 6.50	1941674	1944061	2387	-	10.81	10.89	0.00

tp15	Rivas et al.	3437153	?	?	unknown	yhdN/rplQ Score: 15.03	3436642	3437190	548	+	10.2	9.84	0.00
tp15	Rivas et al.	3437153	?	?	unknown	yhdN/rplQ Score: 15.03	3436492	3437567	1075	-	11.68	11.41	0.00
k43	Rivas et al.	982119	?	?	unknown	ycbB/ycbK Score: 6.43	980128	984931	4803	+	11.41	11.2	0.00
k43	Rivas et al.	982119	?	?	unknown	ycbB/ycbK Score: 6.43	981264	982149	885	-	9.47	9.15	0.01
C0516	Tjaden et al.	2238388	2238521	133	+	mglB/galS	2238390	2238443	53	+	9.44	9.49	0.00
C0650	Tjaden et al.	2773402	2773509	107	-	yfjW/ypjI	2773368	2773543	175	-	11.33	11.35	0.00
C0510	Tjaden et al.	2226932	2227408	476	+	yohG/yohI k3 2227055	2226978	2227043	65	+	11.43	10.42	0.02
C0510	Tjaden et al.	2226932	2227408	476	+	yohG/yohI k3 2227055	2227089	2227130	41	+	11.51	10.6	0.03
C0510	Tjaden et al.	2226932	2227408	476	+	yohG/yohI k3 2227055	2227173	2227420	247	+	10.08	9.86	0.01
C0978	Tjaden et al.	4190298	4190385	87	+	Not intergenic	4190269	4190798	529	+	9.72	9.65	0.00
HB_250	Carter et al.	4323730	4323809	79	+	yjdN/yjdM HB_249 4323690-4323769 +	4323705	4323782	77	+	9.65	9.63	0.00
C0513	Tjaden et al.	2230771	2230854	83	-	cdd/sanA HB_389 2230768-2230847 - HB_390 2230749-2230807 -	2230672	2230859	187	-	9.54	9.33	0.00
C0696	Tjaden et al.	3043939	3044066	127	-	ygfF/gcvP HB_447 3044028-3044107 -	3043139	3047963	4824	-	11.32	11.37	0.00
t1	Rivas et al.	4173138	?	?	unknown	tyrU/glyT Score: 26.36 Experimentally tested	4172793	4176370	3577	+	12.22	11.89	0.00
t4	Rivas et al.	3809317	?	?	unknown	rpmB/radC Score: 21.27 Experimentally tested Resides within an operon	3809279	3809696	417	+	10.39	10.36	0.00
t4	Rivas et al.	3809317	?	?	unknown	rpmB/radC Score: 21.27 Experimentally tested Resides within an operon	3809214	3810055	841	-	13.95	13.69	0.00
HB_523	Carter et al.	4054085	4054164	79	-	glnL/glnA HB_522 4054125-4054204 - Overlaps a 3' UTR HB_524 4054045-4054124 -	4051444	4054400	2956	-	11.28	11.19	0.00
C0077	Tjaden et al.	284425	284500	75	+	yagF/yagG	283429	284455	1026	+	9.71	9.49	0.00
C1079	Tjaden et al.	4567632	4567706	74	+	yjiP/yjiR	4566995	4567876	881	+	10.56	10.45	0.00
psrA7	Argaman et al.	1887849	1887959	110	+	fadD/yeaY IS087 1887856-1887966 +	1887920	1887956	36	+	9.54	9.53	0.00
C0074	Tjaden et al.	268372	268463	91	+	ykfA/perR	268235	268469	234	+	11.03	10.88	0.00
psrA1	Argaman et al.	191713	191793	80	-	tsf/pyrH IS004 191706-191797 -	190755	191791	1036	-	10.32	10.4	0.00
C0072	Tjaden et al.	256435	256515	80	-	gpt/yafA	256296	256503	207	-	9.98	10	0.00
C0070	Tjaden et al.	245831	245907	76	+	yafK/yafQ	245816	245991	175	+	10.58	10.22	0.00
C1070	Tjaden et al.	4532142	4532310	168	+	yjhQ/yjhR	4532054	4532253	199	+	11.42	10.93	0.01
t2	Rivas et al.	454015	?	?	unknown	bolA/tig Score: 24.93 Experimentally tested Resides within an operon	453559	457964	4405	+	12.15	12.07	0.00
t2	Rivas et al.	454015	?	?	unknown	bolA/tig Score: 24.93 Experimentally tested Resides within an operon	453664	454057	393	-	9.81	9.81	0.00
HB_521	Carter et al.	4049437	4049488	51	-	yihI/hemN p1 4049438	4049467	4049568	101	-	10.27	10.03	0.01
psrA9	Argaman et al.	2531422	2531608	186	-	cysK/ptsH	2531366	2531510	144	-	10.59	10.07	0.01
C1075	Tjaden et al.	4549106	4549179	73	+	gntP/uxuA	4548852	4549303	451	+	10.01	9.95	0.00
C0078	Tjaden et al.	289535	289661	126	-	argF/yagJ	289286	290652	1366	-	9.92	9.78	0.00
C0850	Tjaden et al.	3645316	3645610	294	-	gor/arsR	3644506	3645672	1166	-	9.79	9.49	0.00
C0674	Tjaden et al.	2892830	2892926	96	-	ygcP/ygcQ	2892821	2893530	709	-	10.82	10.62	0.00
C0676	Tjaden et al.	2905967	2906047	80	+	eno/pyrG	2905986	2906938	952	+	9.88	9.55	0.01
HB_251	Carter et al.	4323810	4323889	79	+	yjdN/yjdM HB_252 4323850-4323929 +	4323810	4324543	733	+	10.44	10.43	0.00
C0779	Tjaden et al.	3376333	3376491	158	+	rplM/yhcM	3375945	3376799	854	+	10.64	10.47	0.00
C0673	Tjaden et al.	2886336	2886408	72	+	cysH/cysI	2886343	2886408	65	+	10	9.87	0.01
HB_200	Carter et al.	3766346	3766425	79	+	Not intergenic	3766424	3766495	71	+	9.41	9.39	0.00
C1077	Tjaden et al.	4561419	4561465	46	-	yjiK/yjiL	4561415	4561722	307	-	9.86	9.34	0.01
IS029	Chen et al.	752270	752396	126	+	ybgD/gltA	752348	752999	651	+	9.8	9.81	0.00
IS028	Chen et al.	728577	728738	161	-	ybfA/rhsC	728466	728623	157	-	10.2	10.04	0.01
C0288	Tjaden et al.	1179616	1179699	83	-	cobB/ycfZ	1179496	1179769	273	-	9.97	9.64	0.01
C0928	Tjaden et al.	4007804	4007912	108	-	Not intergenic	4007168	4007883	715	-	9.67	9.51	0.00
IS023	Chen et al.	623829	624038	209	-	fepB/entC Overlaps a 5' UTR	622282	624056	1774	-	11.26	11.09	0.00
IS022	Chen et al.	584964	585220	256	+	ompT/envY	584971	585010	39	+	11.22	9.9	0.05
IS022	Chen et al.	584964	585220	256	+	ompT/envY	585029	585072	43	+	12.22	11.79	0.01
IS022	Chen et al.	584964	585220	256	+	ompT/envY	585089	585142	53	+	9.95	9.26	0.02
IS022	Chen et al.	584964	585220	256	+	ompT/envY	585139	585192	53	+	10.66	10.64	0.00
IS022	Chen et al.	584964	585220	256	+	ompT/envY	585198	585265	67	+	11.73	11.62	0.00
IS021	Chen et al.	583781	583900	119	+	appY/ompT	583377	584853	1476	+	11.66	11.61	0.00
IS027	Chen et al.	707168	707344	176	-	glnS/ybfM	707160	707223	63	-	10	10.08	0.00
IS026	Chen et al.	698444	698601	157	-	asnB/nagD	696598	698462	1864	-	10.54	10.36	0.00
IS026	Chen et al.	698444	698601	157	-	asnB/nagD	698509	698545	36	-	10.59	9.81	0.04
IS026	Chen et al.	698444	698601	157	-	asnB/nagD	698577	699352	775	-	10.91	10.73	0.00
IS024	Chen et al.	637912	638025	113	+	dsbG/ahpC Overlaps a 5' UTR	637891	638850	959	+	12.7	12.49	0.00
C1035	Tjaden et al.	4432089	4432180	91	-	ytfH/cpdB	4432142	4432233	91	-	10.43	10.36	0.00
HB_252	Carter et al.	4323850	4323929	79	+	yjdN/yjdM HB_251 4323810-4323889 +	4323810	4324543	733	+	10.44	10.43	0.00

tpke85	Rivas et al.	3081830	?	?	unknown	speB/speA Score: 9.64 Experimentally tested Resides within an operon	3080690	3084140	3450	-	10.64	10.48	0.00
HB_528	Carter et al.	4177364	4177443	79	-	rplA/rplJ C0970 4177248-4177547 -	4176522	4178018	1496	-	10.27	10.06	0.00
HB_529	Carter et al.	4178504	4178572	68	-	rplL/rpoB C0973 4178555-4178804 -	4178083	4178564	481	-	10.07	10.03	0.00
C0937	Tjaden et al.	4041634	4041725	91	-	dsbA/yihF	4041519	4041904	385	-	9.69	9.65	0.00
C0934	Tjaden et al.	4028683	4028710	27	+	fadB/pepQ	4027840	4029036	1196	+	9.94	9.86	0.00
tpke89	Rivas et al.	2744207	?	?	unknown	rpsP/ffh Score: 10.34 Experimentally tested Resides within an operon	2743738	2744272	534	+	9.74	9.75	0.00
tpke89	Rivas et al.	2744207	?	?	unknown	rpsP/ffh Score: 10.34 Experimentally tested Resides within an operon	2742213	2745881	3668	-	12.52	12.26	0.00
HB_553	Carter et al.	4508178	4508257	79	-	yjhE/fecE HB_554 4508138-4508217 -	4507922	4508284	362	-	9.96	9.38	0.01
HB_552	Carter et al.	4506276	4506355	79	-	yjhE/fecE HB_551 4506316-4506395 -	4505141	4506312	1171	-	11.38	11.32	0.00
IS217	Chen et al.	4485969	4486075	106	-	yjgQ/yjgR Overlaps a 3' UTR	4485077	4486250	1173	-	9.67	9.51	0.00
HB_550	Carter et al.	4499271	4499350	79	-	yjgZ/yjhB HB_549 4499311-4499390 -	4499302	4499457	155	-	9.56	9.67	0.00
HB_555	Carter et al.	4517759	4517838	79	-	Not intergenic	4517274	4518425	1151	-	11.01	10.86	0.00
HB_554	Carter et al.	4508138	4508217	79	-	yjhE/fecE HB_553 4508178-4508257 -	4507922	4508284	362	-	9.96	9.38	0.01
HB_559	Carter et al.	4547402	4547481	79	-	fimH/gntP HB_560 4547362-4547441 -	4546766	4547636	870	-	9.76	9.39	0.01
C0881	Tjaden et al.	3737180	3737284	104	-	malS/avtA HB_497 3737157-3737203 -	3737186	3737727	541	-	10.42	10.42	0.00
HB_253	Carter et al.	4329663	4329742	79	+	proP/basS Overlaps a 3' UTR	4329622	4330153	531	+	12.27	11.73	0.01
HB_418	Carter et al.	2651857	2651936	79	-	sseA/sseB IS129 2651687-2652078 -	2651859	2652042	183	-	10.1	9.71	0.01
k10	Rivas et al.	1080042	?	?	unknown	putP/ycdN Score: 13.52	1078996	1080071	1075	+	10.68	10.61	0.00
k10	Rivas et al.	1080042	?	?	unknown	putP/ycdN Score: 13.52	1079897	1080115	218	-	10.83	10.87	0.00
e9	Rivas et al.	3980426	?	?	unknown	proM/aslB Score: 8.54	3978835	3980521	1686	+	11.37	10.92	0.00
e8	Rivas et al.	463012	?	?	unknown	ppiD/ybaV Score: 8.86	459705	463044	3339	+	12.13	11.96	0.00
e8	Rivas et al.	463012	?	?	unknown	ppiD/ybaV Score: 8.86	461100	463036	1936	-	9.99	9.9	0.00
HB_412	Carter et al.	2537657	2537736	79	-	cysM/cysA Resides within an operon	2536503	2538975	2472	-	10.71	10.44	0.00
HB_413	Carter et al.	2561267	2561346	79	-	yffP/yffQ IS125 2561257-2561485 - C0593 2561161-2561457 -	2561236	2561381	145	-	9.98	9.94	0.00
e7	Rivas et al.	1905163	?	?	unknown	rrmA/cspC Score: 9.97	1904967	1905220	253	+	10.16	10.01	0.00
e7	Rivas et al.	1905163	?	?	unknown	rrmA/cspC Score: 9.97	1904011	1905836	1825	-	12.64	12.33	0.00
e6	Rivas et al.	1753526	?	?	unknown	ydhZ/pykF Score: 10.03 Overlaps a 5' UTR	1753467	1755184	1717	+	12.68	12.51	0.00
e6	Rivas et al.	1753526	?	?	unknown	ydhZ/pykF Score: 10.03 Overlaps a 5' UTR	1753427	1753721	294	-	10.79	10.59	0.00
HB_416	Carter et al.	2579674	2579753	79	-	tktB/ypfG Overlaps a 3' UTR	2578492	2580057	1565	-	10.09	9.87	0.00
HB_417	Carter et al.	2598972	2599051	79	-	bcp/hyfA	2593905	2599135	5230	-	11.55	11.45	0.00
e3	Rivas et al.	1018167	?	?	unknown	ycbG/ompA Score: 16.21	1017683	1019305	1622	+	11.43	11.43	0.00
HB_415	Carter et al.	2563381	2563460	79	-	yffS/eutA HB_414 2563421-2563500 -	2562756	2564328	1572	-	9.83	9.76	0.00
HB_456	Carter et al.	3182586	3182665	79	-	ribB/yqiC HB_457 3182546-3182625 - Overlaps a 5' UTR	3181731	3182750	1019	-	12.54	12.4	0.00
IS079	Chen et al.	1695141	1695301	160	+	uidR/hdhA	1695104	1695181	77	+	9.72	9.48	0.01
IS079	Chen et al.	1695141	1695301	160	+	uidR/hdhA	1695218	1695257	39	+	10.13	9.77	0.01
p31	Rivas et al.	1687778	?	?	unknown	manA/ydgA Score: 5.05	1686572	1687803	1231	+	11.28	11.42	0.00
k14	Rivas et al.	3951043	?	?	unknown	ilvE/ilvD Score: 11.45 Resides within an operon	3950274	3954584	4310	+	10.35	10.27	0.00
k14	Rivas et al.	3951043	?	?	unknown	ilvE/ilvD Score: 11.45 Resides within an operon	3950592	3951152	560	-	9.85	9.88	0.00
HB_454	Carter et al.	3161651	3161730	79	-	plsC/parC	3161599	3164035	2436	-	10.53	10.43	0.00
C0924	Tjaden et al.	3972116	3972202	86	-	rffH/rffC	3971762	3972158	396	-	9.96	9.84	0.00
HB_455	Carter et al.	3181349	3181428	79	-	ygiE/ribB IS154 3181333-3181587 -	3180827	3181641	814	-	11.63	11.33	0.00
HB_419	Carter et al.	2660403	2660482	79	-	yfhP/yfhQ Resides within an operon	2660268	2661344	1076	-	12.15	11.78	0.00
HB_452	Carter et al.	3154544	3154623	79	-	yqhD/yqhE	3153384	3154634	1250	-	9.7	9.73	0.00
te23	Rivas et al.	4112054	?	?	unknown	fpr/glpX Score: 5.38	4111970	4112156	186	+	10.25	10.22	0.00
te23	Rivas et al.	4112054	?	?	unknown	fpr/glpX Score: 5.38	4111438	4112348	910	-	10.93	11	0.00
te21	Rivas et al.	3325729	?	?	unknown	yhbY/greA Score: 7.02	3324992	3326230	1238	+	12.51	12.2	0.00
te21	Rivas et al.	3325729	?	?	unknown	yhbY/greA Score: 7.02	3324973	3327254	2281	-	12.09	11.88	0.00
tpke118	Rivas et al.	984934	?	?	unknown	aspC/ompF Score: 9.14 Experimentally tested	983536	984969	1433	-	11.87	11.87	0.00
t49	Rivas et al.	2163581	?	?	unknown	yegP/yegQ HB_384 2163560-2163639 - Score: 7.51	2163459	2165250	1791	+	10.6	10.27	0.00
t49	Rivas et al.	2163581	?	?	unknown	yegP/yegQ HB_384 2163560-2163639 - Score: 7.51	2163542	2163655	113	-	9.88	9.67	0.00
HB_450	Carter et al.	3069319	3069398	79	-	fbaA/pgk Resides within an operon	3068150	3073698	5548	-	12.23	12.17	0.00
IS109	Chen et al.	2190859	2190978	119	-	yehE/mrp	2190803	2190929	126	-	11.88	11.27	0.01
IS109	Chen et al.	2190859	2190978	119	-	yehE/mrp	2190924	2191056	132	-	10.44	9.83	0.01
tp48	Rivas et al.	3516988	?	?	unknown	aroK/hofQ IS173 3516932-3517022 - Score: 6.56 Overlaps a 5' UTR	3510579	3517410	6831	-	11.85	11.7	0.00
HB_451	Carter et al.	3085880	3085932	52	-	metK/galP	3085642	3085932	290	-	9.93	9.9	0.00
HB_451	Carter et al.	3085880	3085932	52	-	metK/galP	3085931	3086064	133	-	10.4	10.31	0.00
t47	Rivas et al.	229006	?	?	unknown	aspU/yafB Score: 7.56	223217	229162	5945	-	10.23	10.08	0.00
t45	Rivas et al.	460949	?	?	unknown	hupB/ppiD Score: 7.80	459705	463044	3339	+	12.13	11.96	0.00

tp49	Rivas et al.	3774748	?	?	unknown	yibL/lldP Score: 6.54	3774629	3775182	553	+	11.61	11.39	0.00
tp49	Rivas et al.	3774748	?	?	unknown	yibL/lldP Score: 6.54	3774700	3774778	78	-	10.84	10.64	0.00
HB_526	Carter et al.	4103901	4103958	57	-	cpxP/yiiP p29 4103904 C0950 4103905-4103989 -	4101389	4104095	2706	-	10.82	10.77	0.00
IS179	Chen et al.	3595386	3595485	99	-	livK/yhhK	3594145	3595750	1605	-	9.87	9.59	0.00
HB_524	Carter et al.	4054045	4054124	79	-	glnL/glnA HB_523 4054085-4054164 - Overlaps a 3' UTR	4051444	4054400	2956	-	11.28	11.19	0.00
HB_399	Carter et al.	2302648	2302727	79	-	eco/yojH HB_398 2302688-2302767 -	2302646	2303930	1284	-	10.39	10.25	0.00
HB_398	Carter et al.	2302688	2302767	79	-	eco/yojH HB_399 2302648-2302727 -	2302646	2303930	1284	-	10.39	10.25	0.00
HB_520	Carter et al.	4010513	4010592	79	-	metR/metE	4009835	4010540	705	-	9.84	9.78	0.00
IS071	Chen et al.	1580770	1580915	145	+	ydeN/ydeO	1580858	1580899	41	+	10.63	10.63	0.00
HB_395	Carter et al.	2302848	2302927	79	-	eco/yojH HB_396 2302808-2302887 -	2302646	2303930	1284	-	10.39	10.25	0.00
HB_394	Carter et al.	2302928	2303007	79	-	eco/yojH HB_393 2302968-2303047 -	2302646	2303930	1284	-	10.39	10.25	0.00
HB_397	Carter et al.	2302768	2302847	79	-	eco/yojH HB_396 2302808-2302887 -	2302646	2303930	1284	-	10.39	10.25	0.00
HB_396	Carter et al.	2302808	2302887	79	-	eco/yojH HB_395 2302848-2302927 - HB_397 2302768-2302847 -	2302646	2303930	1284	-	10.39	10.25	0.00
HB_391	Carter et al.	2234521	2234562	41	-	yeiA/mglC	2234421	2234570	149	-	9.93	9.89	0.00
HB_390	Carter et al.	2230749	2230807	58	-	cdd/sanA HB_389 2230768-2230847 - C0513 2230771-2230854 -	2230672	2230859	187	-	9.54	9.33	0.00
HB_393	Carter et al.	2302968	2303047	79	-	eco/yojH HB_394 2302928-2303007 -	2302646	2303930	1284	-	10.39	10.25	0.00
HB_392	Carter et al.	2244840	2244882	42	-	cirA/lysP Overlaps a 5' UTR	2242562	2244983	2421	-	12.41	12.42	0.00
IS073	Chen et al.	1630377	1630602	225	+	ydfJ/ydfK	1630437	1630482	45	+	9.5	9.04	0.02
t8	Rivas et al.	3440110	?	?	unknown	rpsM/rpmJ Score: 18.39 Experimentally tested Resides within an operon	3437704	3440327	2623	+	10.58	10.53	0.00
t8	Rivas et al.	3440110	?	?	unknown	rpsM/rpmJ Score: 18.39 Experimentally tested Resides within an operon	3437613	3451499	13886	-	13.48	13.29	0.00
HB_159	Carter et al.	2776084	2776163	79	+	ypjF/ypjA	2775996	2776124	128	+	9.58	9.62	0.00
HB_157	Carter et al.	2773723	2773802	79	+	Not intergenic	2773677	2773768	91	+	10.58	10.53	0.00
HB_156	Carter et al.	2773603	2773682	79	+	Not intergenic	2773613	2773660	47	+	9.56	9.59	0.00
HB_156	Carter et al.	2773603	2773682	79	+	Not intergenic	2773677	2773768	91	+	10.58	10.53	0.00
HB_155	Carter et al.	2773243	2773322	79	+	yfjW/ypjI HB_154 2773203-2773282 +	2773276	2773357	81	+	11	9.98	0.02
HB_154	Carter et al.	2773203	2773282	79	+	yfjW/ypjI HB_155 2773243-2773322 +	2773276	2773357	81	+	11	9.98	0.02
HB_153	Carter et al.	2759275	2759354	79	+	yfjJ/yfjK	2759261	2760798	1537	+	10.87	10.51	0.00
HB_152	Carter et al.	2744336	2744415	79	+	rpsP/ffh HB_151 2744296-2744375 +	2744318	2745048	730	+	10.61	10.6	0.00
HB_151	Carter et al.	2744296	2744375	79	+	rpsP/ffh HB_152 2744336-2744415 + C0643 2744245-2744315 +	2744272	2744316	44	+	10.09	9.78	0.01
HB_151	Carter et al.	2744296	2744375	79	+	rpsP/ffh HB_152 2744336-2744415 + C0643 2744245-2744315 +	2744318	2745048	730	+	10.61	10.6	0.00
HB_150	Carter et al.	2723937	2724016	79	+	kgtP/rrfG C0634 2723788-2724007 +	2723859	2724022	163	+	11.75	11.09	0.01
HB_26	Carter et al.	281298	281377	79	+	yagA/yagE	281269	281379	110	+	10.21	9.94	0.01
HB_24	Carter et al.	271960	272035	75	+	perR/ykfC HB_23 271920-271999 + Resides within an operon	271889	272447	558	+	10.36	10.27	0.00
HB_25	Carter et al.	279430	279509	79	+	afuB/yagB	278060	279943	1883	+	10.11	10.03	0.00
HB_22	Carter et al.	271880	271959	79	+	perR/ykfC HB_23 271920-271999 + Resides within an operon	271841	271883	42	+	9.76	9.79	0.00
HB_22	Carter et al.	271880	271959	79	+	perR/ykfC HB_23 271920-271999 + Resides within an operon	271889	272447	558	+	10.36	10.27	0.00
HB_23	Carter et al.	271920	271999	79	+	perR/ykfC HB_24 271960-272035 + Resides within an operon HB_22 271880-271959 +	271889	272447	558	+	10.36	10.27	0.00
HB_20	Carter et al.	271680	271759	79	+	perR/ykfC HB_21 271720-271799 + Resides within an operon HB_19 271640-271719 +	269421	271808	2387	+	10.63	10.65	0.00
HB_21	Carter et al.	271720	271799	79	+	perR/ykfC HB_20 271680-271759 + Resides within an operon	269421	271808	2387	+	10.63	10.65	0.00
t36	Rivas et al.	2309559	?	?	unknown	apbE/ompC Score: 9.13	2308447	2309581	1134	-	10.34	9.98	0.00
t37	Rivas et al.	1902772	?	?	unknown	manZ/yobD Score: 8.82	1899945	1902995	3050	+	11.9	11.95	0.00
t37	Rivas et al.	1902772	?	?	unknown	manZ/yobD Score: 8.82	1902511	1902815	304	-	9.94	10.06	0.00
t34	Rivas et al.	4465014	?	?	unknown	treR/mgtA HB_546 4464989-4465068 - Score: 9.32 C1051 4464974-4465173 +	4464995	4465307	312	+	9.7	9.66	0.00
t34	Rivas et al.	4465014	?	?	unknown	treR/mgtA HB_546 4464989-4465068 - Score: 9.32 C1051 4464974-4465173 +	4464681	4465384	703	-	9.95	9.67	0.00
HB_510	Carter et al.	3882482	3882534	52	-	Not intergenic	3882406	3883013	607	-	10.17	9.88	0.00
t33	Rivas et al.	4117936	?	?	unknown	menA/hslU Score: 9.43	4117934	4118082	148	+	10.51	10.55	0.00
t33	Rivas et al.	4117936	?	?	unknown	menA/hslU Score: 9.43	4116639	4118419	1780	-	10.76	10.55	0.00
HB_29	Carter et al.	289620	289699	79	+	argF/yagJ IS008 289594-289856 +	289618	289869	251	+	10.53	10.56	0.00
C0157	Tjaden et al.	683647	683729	82	+	ybeK/gltL	683645	683739	94	+	10.69	10.58	0.00
C0258	Tjaden et al.	1050518	1050678	160	-	cspH/cspG IS043 1050440-1050555 -	1050655	1050971	316	-	10.23	10.34	0.00
C0526	Tjaden et al.	2282002	2282129	127	+	yejK/yejL	2281829	2284340	2511	+	11.65	11.44	0.00
C0999	Tjaden et al.	4282864	4282978	114	+	yjcH/acs	4282126	4282898	772	+	9.53	9.55	0.00
C0252	Tjaden et al.	1029189	1029267	78	+	yccW/yccX	1028975	1030613	1638	+	10.68	10.57	0.00
C0250	Tjaden et al.	1019457	1019577	120	+	ompA/sulA	1019439	1019502	63	+	9.56	9.23	0.01

C0250	Tjaden et al.	1019457	1019577	120	+	ompA/sulA	1019511	1020174	663	+	10.7	10.53	0.00
tpe40	Rivas et al.	1994067	?	?	unknown	yecF/sdiA Score: 12.29	1993767	1994380	613	+	11.06	10.66	0.00
tpe40	Rivas et al.	1994067	?	?	unknown	yecF/sdiA Score: 12.29	1993997	1994897	900	-	11.13	10.96	0.00
C0488	Tjaden et al.	2076954	2077027	73	+	yoeD/yeeX	2076936	2077008	72	+	10.51	10.09	0.01
C0846	Tjaden et al.	3635080	3635116	36	-	yhiN/pitA	3634290	3635507	1217	-	10.12	9.96	0.00
tp40	Rivas et al.	4327822	?	?	unknown	yjcZ/proP Score: 8.12	4325245	4328300	3055	+	11.81	11.5	0.00
IS135	Chen et al.	2759199	2759300	101	-	yfjJ/yfjK	2759261	2763399	4138	-	11.33	11.04	0.00
IS211	Chen et al.	4304230	4304455	225	+	yjcS/alsK	4303614	4304518	904	+	9.8	9.74	0.00
C0480	Tjaden et al.	2039194	2039225	31	-	yedZ/yodA	2038679	2039262	583	-	9.81	9.71	0.00
C0481	Tjaden et al.	2040047	2040135	88	-	yodA/yodB	2040052	2040156	104	-	9.97	9.98	0.00
C0486	Tjaden et al.	2072681	2072772	91	-	flu/yeeR	2070620	2072778	2158	-	10.63	10.49	0.00
C0487	Tjaden et al.	2076255	2076327	72	-	yeeW/yoeD HB_374 2076254-2076333 -	2076231	2076278	47	-	9.95	9.91	0.00
C0487	Tjaden et al.	2076255	2076327	72	-	yeeW/yoeD HB_374 2076254-2076333 -	2076279	2076335	56	-	9.84	9.81	0.00
C0485	Tjaden et al.	2060175	2060249	74	-	nac/asnV	2059671	2060209	538	-	10.2	9.81	0.00
C0485	Tjaden et al.	2060175	2060249	74	-	nac/asnV	2060218	2060281	63	-	11.24	10.85	0.01
HB_320	Carter et al.	970822	970884	62	-	kdsB/ycbJ	970827	970890	63	-	9.17	9.16	0.00
HB_321	Carter et al.	1006937	1007016	79	-	ycbX/ycbY	1006891	1007859	968	-	9.53	9.24	0.01
HB_322	Carter et al.	1049080	1049159	79	-	ymcD/cspH HB_323 1049040-1049119 -	1048707	1049879	1172	-	12.26	11.86	0.00
HB_323	Carter et al.	1049040	1049119	79	-	ymcD/cspH HB_322 1049080-1049159 -	1048707	1049879	1172	-	12.26	11.86	0.00
HB_324	Carter et al.	1080160	1080239	79	-	putP/ycdN C0267 1080196-1080293 -	1080120	1080506	386	-	11	10.74	0.00
HB_325	Carter et al.	1164978	1165057	79	-	ycfP/ndh	1164130	1165066	936	-	9.99	9.96	0.00
HB_326	Carter et al.	1166692	1166771	79	-	ndh/ycfJ HB_327 1166652-1166731 -	1166620	1166793	173	-	9.86	9.47	0.01
HB_327	Carter et al.	1166652	1166731	79	-	ndh/ycfJ HB_326 1166692-1166771 -	1166620	1166793	173	-	9.86	9.47	0.01
HB_328	Carter et al.	1201334	1201401	67	-	ymfI/ymfK	1200637	1201412	775	-	10.84	10.9	0.00
HB_329	Carter et al.	1269652	1269731	79	-	kdsA/chaA HB_330 1269612-1269691 -	1268279	1269894	1615	-	10.22	10.03	0.01
C0412	Tjaden et al.	1756794	1756890	96	+	ynhG/ynhA	1755406	1756833	1427	+	11.68	11.58	0.00
C0412	Tjaden et al.	1756794	1756890	96	+	ynhG/ynhA	1756848	1756895	47	+	9.69	9.58	0.00
C0417	Tjaden et al.	1787527	1787597	70	-	aroH/ydiE	1787549	1787589	40	-	10.91	10.36	0.02
C0645	Tjaden et al.	2751490	2751789	299	+	Not intergenic	2751512	2752043	531	+	12.86	12.67	0.00
IS224	Chen et al.	4566199	4566339	140	-	yjiO/yjiP C1078 4566160-4566320 -	4566009	4566596	587	-	9.92	9.58	0.01
HB_259	Carter et al.	4448496	4448575	79	+	ytfQ/ytfR Resides within an operon	4448558	4449151	593	+	10.47	10.38	0.00
tp20	Rivas et al.	4362936	?	?	unknown	cutA/dcuA Score: 13.50	4361476	4363528	2052	+	10.06	10.05	0.00
tp20	Rivas et al.	4362936	?	?	unknown	cutA/dcuA Score: 13.50	4360656	4364749	4093	-	11.07	10.87	0.00
C0736	Tjaden et al.	3229118	3229257	139	-	ygjK/fadH HB_459 3229176-3229255 -	3229157	3229664	507	-	9.87	9.82	0.00
C0737	Tjaden et al.	3229172	3229264	92	+	ygjK/fadH	3228761	3229642	881	+	10.04	9.79	0.00
k27	Rivas et al.	2720642	?	?	unknown	yfiQ/pssA Score: 8.61 Resides within an operon	2718474	2722205	3731	+	11.51	11.36	0.00
k27	Rivas et al.	2720642	?	?	unknown	yfiQ/pssA Score: 8.61 Resides within an operon	2720478	2722139	1661	-	10	9.88	0.00
IS105	Chen et al.	2151195	2151378	183	+	yegL/yegM tp11 2151247 HB_125 2151321-2151400 +	2151207	2151265	58	+	9.98	9.88	0.00
IS105	Chen et al.	2151195	2151378	183	+	yegL/yegM tp11 2151247 HB_125 2151321-2151400 +	2151275	2151589	314	+	10.12	9.9	0.00
IS107	Chen et al.	2175335	2175430	95	-	gatY/fbaB	2175395	2176693	1298	-	11.15	11.04	0.00
IS069	Chen et al.	1550236	1550402	166	+	fdnI/yddM	1550209	1550246	37	+	9.64	9.18	0.02
IS141	Chen et al.	2876310	2876479	169	-	iap/ygbF HB_433 2876312-2876391 - HB_434 2876272-2876351 - HB_435 2876232-2876311 -	2876139	2876496	357	-	10.6	10.38	0.00
IS067	Chen et al.	1489461	1489633	172	-	cybB/ydcA	1489488	1489539	51	-	9.96	9.7	0.01
IS067	Chen et al.	1489461	1489633	172	-	cybB/ydcA	1489556	1489628	72	-	10.76	10.45	0.01
IS064	Chen et al.	1449521	1449621	100	+	tynA/maoC	1449454	1449546	92	+	10.47	10.31	0.00
IS108	Chen et al.	2190381	2190515	134	+	yehD/yehE	2190513	2190549	36	+	9.64	9.97	0.02
IS062	Chen et al.	1432742	1433125	383	-	ynaE/ynaF	1432015	1432801	786	-	13.54	12.24	0.03
IS062	Chen et al.	1432742	1433125	383	-	ynaE/ynaF	1433116	1433715	599	-	11.93	11.64	0.00
C0325	Tjaden et al.	1321159	1321239	80	-	trpL/trpH	1321166	1322249	1083	-	10.1	9.88	0.00
IS060	Chen et al.	1349187	1349375	188	+	fabI/ycjD	1349154	1349392	238	+	10.75	10.44	0.00
k35	Rivas et al.	1300839	?	?	unknown	oppA/oppB Score: 7.44 Resides within an operon	1298977	1304834	5857	+	12.24	11.96	0.00
k35	Rivas et al.	1300839	?	?	unknown	oppA/oppB Score: 7.44 Resides within an operon	1299173	1301843	2670	-	10.26	10	0.00
k36	Rivas et al.	668153	?	?	unknown	Not intergenic Score: 7.40 Overlaps a 5' UTR	668149	668204	55	+	10.56	10.33	0.01
k36	Rivas et al.	668153	?	?	unknown	Not intergenic Score: 7.40 Overlaps a 5' UTR	663289	674202	10913	-	11.21	10.98	0.00
k31	Rivas et al.	3034304	?	?	unknown	prfB/recJ Score: 7.74 Overlaps a 5' UTRResides within an operon	3031679	3038740	7061	-	11.03	10.96	0.00
k30	Rivas et al.	2403505	?	?	unknown	nuoA/lrhA IS115 2403317-2403693 + Score: 7.95 Overlaps a 5' UTR	2403461	2404171	710	+	11.47	11.2	0.00
k33	Rivas et al.	1318464	?	?	unknown	Not intergenic Score: 7.49	1318356	1320623	2267	+	9.93	10.28	0.01

k33	Rivas et al.	1318464	?	?	unknown	Not intergenic Score: 7.49	1314370	1320532	6162	-	11.71	12	0.00
k32	Rivas et al.	643240	?	?	unknown	rnk/rna Score: 7.56 Resides within an operon	643059	643300	241	+	10.09	9.95	0.00
k32	Rivas et al.	643240	?	?	unknown	rnk/rna Score: 7.56 Resides within an operon	642782	644262	1480	-	11.14	11	0.00
IS144	Chen et al.	2931888	2932014	126	-	fucA/fucP	2931939	2931978	39	-	9.44	9.63	0.01
k39	Rivas et al.	4003412	?	?	unknown	pldA/recQ Score: 7.11	4002750	4003785	1035	+	10.89	10.71	0.00
k39	Rivas et al.	4003412	?	?	unknown	pldA/recQ Score: 7.11	4003012	4003683	671	-	9.9	9.81	0.00
HB_462	Carter et al.	3232300	3232379	79	-	ygjN/ygjO	3232207	3232327	120	-	10.22	10.01	0.00
HB_462	Carter et al.	3232300	3232379	79	-	ygjN/ygjO	3232339	3232499	160	-	10.5	10.39	0.00
k3	Rivas et al.	2227055	?	?	unknown	yohG/yohI C0510 2226932-2227408 + Score: 20.83 Experimentally tested	2227002	2227065	63	-	10.02	9.76	0.01
HB_38	Carter et al.	547692	547771	79	+	Not intergenic HB_39 547732-547787 +	547678	547850	172	+	10.34	10.28	0.00
k7	Rivas et al.	2280826	?	?	unknown	rplY/yejK Score: 14.16	2278629	2280907	2278	+	10.91	10.73	0.00
k7	Rivas et al.	2280826	?	?	unknown	rplY/yejK Score: 14.16	2280446	2280854	408	-	10.28	10	0.00
k6	Rivas et al.	3087700	?	?	unknown	galP/sprT Score: 14.60	3086251	3087978	1727	+	10.41	10.3	0.00
k6	Rivas et al.	3087700	?	?	unknown	galP/sprT Score: 14.60	3086477	3087937	1460	-	10.14	9.97	0.00
k5	Rivas et al.	4353991	?	?	unknown	yjdL/cadA Score: 16.41	4353652	4354010	358	+	9.92	9.88	0.00
k5	Rivas et al.	4353991	?	?	unknown	yjdL/cadA Score: 16.41	4353933	4354066	133	-	9.63	9.66	0.00
k4	Rivas et al.	3436082	?	?	unknown	Not intergenic Score: 19.34 Experimentally tested	3435985	3436558	573	+	11.68	11.58	0.00
k4	Rivas et al.	3436082	?	?	unknown	Not intergenic Score: 19.34 Experimentally tested	3436008	3436494	486	-	10.45	10.03	0.01
HB_563	Carter et al.	4611064	4611143	79	-	Not intergenic	4610455	4611558	1103	-	9.93	9.79	0.00
k9	Rivas et al.	3717990	?	?	unknown	cspA/yiaZ Score: 13.73	3717864	3718281	417	+	14.13	13.73	0.00
C1051	Tjaden et al.	4464974	4465173	199	+	treR/mgtA t34 4465014	4464995	4465307	312	+	9.7	9.66	0.00
C1042	Tjaden et al.	4448591	4448625	34	-	ytfQ/ytfR	4447950	4449563	1613	-	9.8	9.72	0.00
HB_188	Carter et al.	3576488	3576567	79	+	gntR/yhhW HB_189 3576528-3576580 + C0835 3576479-3576551 +	3576333	3576677	344	+	9.83	9.79	0.00
HB_189	Carter et al.	3576528	3576580	52	+	gntR/yhhW HB_188 3576488-3576567 + C0835 3576479-3576551 +	3576333	3576677	344	+	9.83	9.79	0.00
HB_185	Carter et al.	3483506	3483585	79	+	yhfA/crp	3483467	3483837	370	+	10.15	10.14	0.00
HB_186	Carter et al.	3502502	3502572	70	+	yhfR/yhfS	3502020	3502867	847	+	10.25	10.21	0.00
HB_187	Carter et al.	3550157	3550236	79	+	malP/malT	3548971	3550521	1550	+	9.52	9.28	0.00
HB_180	Carter et al.	3192742	3192821	79	+	yqiK/rfaE HB_179 3192702-3192781 +	3192640	3192783	143	+	10.27	10.1	0.00
HB_181	Carter et al.	3232267	3232346	79	+	ygjN/ygjO C0738 3232214-3232289 +	3232331	3232426	95	+	9.96	9.8	0.00
HB_183	Carter et al.	3237308	3237387	79	+	ygjT/ygjU	3237147	3237344	197	+	9.82	9.29	0.01
C0740	Tjaden et al.	3237188	3237292	104	-	ygjT/ygjU HB_465 3237214-3237293 - HB_464 3237254-3237333 -	3236826	3237772	946	-	9.7	9.6	0.00
C0027	Tjaden et al.	77461	77533	72	+	yabN/yabM	77356	79044	1688	+	10.64	10.59	0.00
HB_209	Carter et al.	3906125	3906176	51	+	pstB/pstA HB_208 3906085-3906164 +	3906018	3906285	267	+	9.51	9.26	0.01
C0023	Tjaden et al.	70222	70340	118	+	araB/araC	70218	70349	131	+	9.9	10.14	0.00
HB_445	Carter et al.	3040429	3040508	79	-	ygfZ/yqfA Overlaps a 3' UTR	3039768	3042081	2313	-	11.78	11.57	0.00
IS219	Chen et al.	4501193	4501457	264	+	yjgZ/yjhB	4501123	4501243	120	+	10.4	10.39	0.00
IS219	Chen et al.	4501193	4501457	264	+	yjgZ/yjhB	4501327	4501438	111	+	9.45	9.53	0.00
psrA19	Argaman et al.	3850744	3850913	169	-	ivbL/ysdA	3848827	3851045	2218	-	10.56	10.62	0.00
HB_245	Carter et al.	4323410	4323489	79	+	yjdN/yjdM HB_244 4323370-4323449 +	4323273	4323413	140	+	9.77	9.72	0.00
HB_245	Carter et al.	4323410	4323489	79	+	yjdN/yjdM HB_244 4323370-4323449 +	4323473	4323658	185	+	9.97	9.89	0.00
tp54	Rivas et al.	2821794	?	?	unknown	recA/ygaD Score: 5.32	2820773	2821830	1057	+	9.88	10.01	0.00
tp54	Rivas et al.	2821794	?	?	unknown	recA/ygaD Score: 5.32	2821637	2821856	219	-	11.7	11.85	0.00
C0840	Tjaden et al.	3598435	3598507	72	+	rpoH/ftsX tpe86 3598462	3598201	3599013	812	+	10.53	10.35	0.00
C0943	Tjaden et al.	4055836	4055961	125	+	glnA/typA	4054942	4056164	1222	+	10.09	9.85	0.00
IS222	Chen et al.	4539345	4539532	187	+	fimB/fimE	4538611	4539634	1023	+	12.43	12.03	0.00
C0940	Tjaden et al.	4050998	4051342	344	-	hemN/glnG	4050900	4051297	397	-	9.87	9.57	0.01
C0947	Tjaden et al.	4090628	4090697	69	+	frvA/yiiL	4090622	4090817	195	+	9.56	9.55	0.00
k8	Rivas et al.	3184109	?	?	unknown	Not intergenic Score: 13.91	3184107	3184152	45	-	10.39	10.26	0.00
HB_287	Carter et al.	16671	16750	79	-	dnaJ/gef	16429	17146	717	-	10.93	10.59	0.00
C1011	Tjaden et al.	4346531	4346635	104	-	dcuB/dcuR	4346377	4346834	457	-	10.53	10.39	0.00
HB_208	Carter et al.	3906085	3906164	79	+	pstB/pstA HB_209 3906125-3906176 + HB_207 3906045-3906124 +	3906018	3906285	267	+	9.51	9.26	0.01
tpke51	Rivas et al.	4225207	?	?	unknown	metH/yjbB Score: 15.79 Experimentally testedOverlaps a 5' UTR	4221715	4225534	3819	+	10.34	10.11	0.00
IS166	Chen et al.	3358366	3358576	210	-	gltD/gltF	3358359	3358750	391	-	10.64	10.38	0.00
HB_298	Carter et al.	281311	281390	79	-	yagA/yagE HB_299 281271-281350 -	281233	281338	105	-	9.98	10.04	0.00
HB_298	Carter et al.	281311	281390	79	-	yagA/yagE HB_299 281271-281350 -	281365	281437	72	-	9.8	9.8	0.00
HB_299	Carter et al.	281271	281350	79	-	yagA/yagE HB_298 281311-281390 -	281233	281338	105	-	9.98	10.04	0.00
HB_111	Carter et al.	1876925	1877004	79	+	yeaP/yeaQ	1876785	1876991	206	+	12.11	11.64	0.00

HB_110	Carter et al.	1859567	1859646	79	+	ydjL/yeaC	1859478	1859571	93	+	10.43	10.37	0.00
HB_110	Carter et al.	1859567	1859646	79	+	ydjL/yeaC	1859586	1859689	103	+	10.12	9.97	0.00
HB_117	Carter et al.	2076717	2076796	79	+	yoeD/yeeX	2076715	2076790	75	+	9.6	9.52	0.00
HB_116	Carter et al.	2066434	2066513	79	+	yeeH/yoeA	2066432	2066507	75	+	9.94	9.91	0.00
HB_115	Carter et al.	1995026	1995084	58	+	sdiA/yecC IS094 1994968-1995087 +	1994996	1995978	982	+	10.57	10.34	0.00
HB_442	Carter et al.	2902089	2902168	79	-	ygcE/ygcF	2902022	2902448	426	-	11.51	11.06	0.00
HB_290	Carter et al.	182315	182394	79	-	degP/yaeG tpe90 182320 HB_291 182309-182354 -	182120	182350	230	-	9.55	9.53	0.00
HB_290	Carter et al.	182315	182394	79	-	degP/yaeG tpe90 182320 HB_291 182309-182354 -	182358	182398	40	-	10.43	10.07	0.01
HB_291	Carter et al.	182309	182354	45	-	degP/yaeG HB_290 182315-182394 - tpe90 182320	182120	182350	230	-	9.55	9.53	0.00
HB_292	Carter et al.	214001	214080	79	-	Not intergenic Overlaps a 5' UTR	213678	214196	518	-	11.54	11.67	0.00
HB_293	Carter et al.	223441	223520	79	-	yaeD/rrsH	223217	229162	5945	-	10.23	10.08	0.00
HB_294	Carter et al.	239339	239418	79	-	Not intergenic	239359	240214	855	-	11.05	10.96	0.00
HB_295	Carter et al.	263320	263399	79	-	Not intergenic	263134	264069	935	-	9.57	9.41	0.00
HB_296	Carter et al.	271480	271555	75	-	perR/ykfC	269437	271550	2113	-	10.07	9.95	0.00
HB_297	Carter et al.	279369	279448	79	-	afuB/yagB Overlaps a 5' UTR	278024	280043	2019	-	10.59	10.71	0.00
e19	Rivas et al.	3306567	?	?	unknown	yhbM/pnp Score: 5.32 Resides within an operon	3305197	3306678	1481	+	10.55	10.15	0.00
e19	Rivas et al.	3306567	?	?	unknown	yhbM/pnp Score: 5.32 Resides within an operon	3306073	3309331	3258	-	12.52	12.2	0.00
HB_237	Carter et al.	4228806	4228885	79	+	yjbC/yjbD HB_238 4228846-4228925 +	4228413	4228872	459	+	10.7	10.67	0.00
e11	Rivas et al.	2937334	?	?	unknown	fucU/fucR Score: 7.83	2935981	2937551	1570	+	10.49	10.68	0.00
e11	Rivas et al.	2937334	?	?	unknown	fucU/fucR Score: 7.83	2937333	2937377	44	-	10.12	10.1	0.00
e10	Rivas et al.	2496317	?	?	unknown	yfdZ/ypdA Score: 7.86	2496042	2496390	348	+	10	9.93	0.00
e10	Rivas et al.	2496317	?	?	unknown	yfdZ/ypdA Score: 7.86	2495127	2496374	1247	-	10.75	10.8	0.00
e12	Rivas et al.	2434671	?	?	unknown	usg/pdxB Score: 7.49 Resides within an operon	2426094	2435943	9849	-	11.04	10.87	0.00
e15	Rivas et al.	3844796	?	?	unknown	uhpT/uhpC Score: 6.53	3844053	3845112	1059	+	9.5	9.39	0.00
e16	Rivas et al.	829880	?	?	unknown	ybiH/rhlE Score: 5.82	824039	829913	5874	-	10.65	10.46	0.00
IS085	Chen et al.	1804122	1804298	176	-	ydiY/pfkB	1803984	1804198	214	-	11.8	11.28	0.01
HB_68	Carter et al.	836740	836819	79	+	ybiC/ybiJ C0194 836732-836807 +	835541	836780	1239	+	10.75	10.76	0.00
HB_69	Carter et al.	846393	846472	79	+	glnP/glnH C0197 846352-846479 +	846381	846703	322	+	10.33	10.22	0.00
IS081	Chen et al.	1739222	1739379	157	+	ydhC/cfa Overlaps a 5' UTR	1739206	1739250	44	+	12.7	11.45	0.04
IS081	Chen et al.	1739222	1739379	157	+	ydhC/cfa Overlaps a 5' UTR	1739251	1740912	1661	+	12.5	12.29	0.00
tke2	Rivas et al.	257	?	?	unknown	thrL/thrA Score: 15.16 Resides within an operon	119	4919	4800	+	10.95	10.86	0.00
tke2	Rivas et al.	257	?	?	unknown	thrL/thrA Score: 15.16 Resides within an operon	129	314	185	-	10.18	9.63	0.01
tke3	Rivas et al.	4370252	?	?	unknown	groL/yjeI Score: 14.91	4369066	4370413	1347	-	10.09	10.44	0.00
HB_62	Carter et al.	779893	779937	44	+	lysT/valT HB_61 779853-779932 +	773944	779900	5956	+	12.09	11.86	0.00
HB_63	Carter et al.	780668	780747	79	+	lysZ/lysQ	779956	781073	1117	+	13.36	12.74	0.00
HB_60	Carter et al.	754302	754349	47	+	gltA/sdhC HB_59 754262-754341 + Overlaps a 5' UTR	754259	754333	74	+	10.21	9.96	0.01
tpe90	Rivas et al.	182320	?	?	unknown	degP/yaeG HB_290 182315-182394 - Score: 6.62<br>HB_291 182309-182354 -	180772	182358	1586	+	11.02	11.03	0.00
tpe90	Rivas et al.	182320	?	?	unknown	degP/yaeG HB_290 182315-182394 - Score: 6.62<br>HB_291 182309-182354 -	182120	182350	230	-	9.55	9.53	0.00
HB_66	Carter et al.	812251	812330	79	+	bioD/uvrB IS035 812251-812372 + HB_67 812291-812370 +	812273	812334	61	+	10.68	10.72	0.00
HB_67	Carter et al.	812291	812370	79	+	bioD/uvrB IS035 812251-812372 + HB_66 812251-812330 +	812273	812334	61	+	10.68	10.72	0.00
HB_64	Carter et al.	780916	780995	79	+	lysQ/nadA	779956	781073	1117	+	13.36	12.74	0.00
HB_65	Carter et al.	791449	791528	79	+	galE/modF	791445	791523	78	+	10.41	10.27	0.00
C0118	Tjaden et al.	497096	497144	48	-	adk/hemH	496374	497144	770	-	9.94	9.9	0.00
pk1	Rivas et al.	502518	?	?	unknown	ybaL/fsr Score: 14.54	500066	502526	2460	-	10.49	10.34	0.00
tp10	Rivas et al.	3808781	?	?	unknown	mutM/rpmG Score: 16.00	3807732	3808807	1075	+	9.97	9.8	0.00
tp10	Rivas et al.	3808781	?	?	unknown	mutM/rpmG Score: 16.00	3808370	3809206	836	-	10.07	9.86	0.00
pk4	Rivas et al.	1712299	?	?	unknown	ydgR/gst Score: 7.63	1710674	1712324	1650	+	12.93	12.53	0.00
C0446	Tjaden et al.	1923028	1923126	98	-	yobA/holE	1923057	1923362	305	-	10.12	10.32	0.00
C0111	Tjaden et al.	489137	489315	178	-	aefA/ybaM	489146	490010	864	-	10.27	10.16	0.00
C0112	Tjaden et al.	490516	490630	114	-	ybaN/apt	490521	493298	2777	-	10.16	9.96	0.00
C0114	Tjaden et al.	491249	491314	65	-	apt/dnaX	490521	493298	2777	-	10.16	9.96	0.00
C0443	Tjaden et al.	1913291	1913450	159	+	Not intergenic	1911318	1913394	2076	+	10.37	10.26	0.00
C0116	Tjaden et al.	496227	496317	90	-	htpG/adk	496042	496325	283	-	10.31	10.26	0.00
C0441	Tjaden et al.	1906979	1907060	81	+	yobG/kdgR	1906969	1907115	146	+	10.84	10.66	0.00
HB_364	Carter et al.	1797090	1797169	79	-	pheS/pheM HB_365 1797050-1797129 - Resides within an operon	1790236	1797387	7151	-	11.17	11.04	0.00
tk7	Rivas et al.	191710	?	?	unknown	tsf/pyrH IS004 191706-191797 - Score: 13.03 Resides within an operon	189776	194806	5030	+	12.4	12.29	0.00

tk7	Rivas et al.	191710	?	?	unknown	tsf/pyrH IS004 191706-191797 - Score: 13.03 Resides within an operon	190755	191791	1036	-	10.32	10.4	0.00
HB_366	Carter et al.	1808073	1808132	59	-	yniC/ydjM	1807404	1808117	713	-	9.44	9.42	0.00
tk5	Rivas et al.	921522	?	?	unknown	ybjZ/cspD Score: 13.88	921485	921909	424	-	11.61	11.58	0.00
HB_360	Carter et al.	1716397	1716476	79	-	ydhA/ydhH	1712608	1716957	4349	-	10.94	10.86	0.00
HB_361	Carter et al.	1739267	1739346	79	-	ydhC/cfa	1739251	1739289	38	-	9.14	9.16	0.00
HB_361	Carter et al.	1739267	1739346	79	-	ydhC/cfa	1739291	1740311	1020	-	9.7	9.46	0.00
HB_362	Carter et al.	1753592	1753671	79	-	ydhZ/pykF	1753427	1753721	294	-	10.79	10.59	0.00
HB_363	Carter et al.	1797170	1797249	79	-	pheS/pheM te16 1797175 Resides within an operon	1790236	1797387	7151	-	11.17	11.04	0.00
HB_368	Carter et al.	1848718	1848793	75	-	sppA/ansA	1847064	1848858	1794	-	10.06	10.02	0.00
HB_369	Carter et al.	1913215	1913294	79	-	Not intergenic Resides within an operon	1910728	1914199	3471	-	12.45	12.24	0.00
tk8	Rivas et al.	661867	?	?	unknown	ybeD/dacA Score: 12.21 Resides within an operon	661495	663281	1786	-	12.23	11.82	0.00
tk9	Rivas et al.	3404907	?	?	unknown	accC/yhdT Score: 12.00	3403156	3405368	2212	+	11.9	11.51	0.00
tk9	Rivas et al.	3404907	?	?	unknown	accC/yhdT Score: 12.00	3403904	3405094	1190	-	10	9.68	0.00
HB_166	Carter et al.	2876042	2876121	79	+	iap/ygbF	2875920	2876131	211	+	10.17	10.06	0.00
HB_167	Carter et al.	2876122	2876201	79	+	iap/ygbF HB_168 2876162-2876241 +	2875920	2876131	211	+	10.17	10.06	0.00
HB_167	Carter et al.	2876122	2876201	79	+	iap/ygbF HB_168 2876162-2876241 +	2876139	2876205	66	+	10.55	10.47	0.00
HB_164	Carter et al.	2875642	2875721	79	+	iap/ygbF	2874565	2875883	1318	+	10.39	10.27	0.00
HB_165	Carter et al.	2875922	2876001	79	+	iap/ygbF	2875920	2876131	211	+	10.17	10.06	0.00
HB_162	Carter et al.	2816918	2816982	64	+	serV/csrA	2816835	2816955	120	+	10.64	10.25	0.01
HB_162	Carter et al.	2816918	2816982	64	+	serV/csrA	2816957	2817100	143	+	10.28	10.23	0.00
HB_163	Carter et al.	2833069	2833148	79	+	ygbD/hypF	2832780	2833186	406	+	9.79	9.62	0.00
HB_160	Carter et al.	2792077	2792156	79	+	gabT/gabP HB_161 2792117-2792196 +	2789901	2792183	2282	+	10.45	10.36	0.00
HB_161	Carter et al.	2792117	2792196	79	+	gabT/gabP HB_160 2792077-2792156 +	2789901	2792183	2282	+	10.45	10.36	0.00
HB_207	Carter et al.	3906045	3906124	79	+	pstB/pstA HB_208 3906085-3906164 +	3906018	3906285	267	+	9.51	9.26	0.01
HB_206	Carter et al.	3881768	3881847	79	+	dnaA/rpmH Overlaps a 5' UTR	3880809	3881780	971	+	9.6	9.39	0.00
HB_206	Carter et al.	3881768	3881847	79	+	dnaA/rpmH Overlaps a 5' UTR	3881809	3881880	71	+	10.67	10.5	0.00
HB_205	Carter et al.	3881408	3881487	79	+	dnaA/rpmH	3880809	3881780	971	+	9.6	9.39	0.00
HB_204	Carter et al.	3851106	3851185	79	+	Not intergenic C0900 3851123-3851297 +	3851128	3851207	79	+	9.84	9.79	0.00
HB_203	Carter et al.	3850746	3850825	79	+	ivbL/ysdA	3849695	3851037	1342	+	9.73	9.63	0.00
HB_202	Carter et al.	3834024	3834103	79	+	selC/yicK	3833514	3834034	520	+	10	9.91	0.00
HB_202	Carter et al.	3834024	3834103	79	+	selC/yicK	3834051	3834096	45	+	9.8	9.7	0.00
HB_168	Carter et al.	2876162	2876241	79	+	iap/ygbF HB_167 2876122-2876201 +	2876139	2876205	66	+	10.55	10.47	0.00
HB_168	Carter et al.	2876162	2876241	79	+	iap/ygbF HB_167 2876122-2876201 +	2876227	2876496	269	+	9.98	9.74	0.00
HB_169	Carter et al.	2876322	2876401	79	+	iap/ygbF	2876227	2876496	269	+	9.98	9.74	0.00
HB_422	Carter et al.	2754010	2754089	79	-	smpB/intA	2753858	2754097	239	-	9.91	9.78	0.00
pe16	Rivas et al.	3382141	?	?	unknown	mdh/argR Score: 5.70	3382027	3382228	201	+	10.39	9.84	0.02
pe16	Rivas et al.	3382141	?	?	unknown	mdh/argR Score: 5.70	3380502	3382191	1689	-	10.29	10.39	0.00
pe14	Rivas et al.	3255714	?	?	unknown	yhaO/tdcG Score: 7.10	3255334	3256020	686	-	9.9	9.48	0.01
IS206	Chen et al.	4187977	4188079	102	+	htrC/thiH	4187719	4188388	669	+	10.75	10.44	0.00
pe10	Rivas et al.	963339	?	?	unknown	ihfB/ycaI Score: 10.02	960353	963380	3027	+	12.88	12.76	0.00
pe11	Rivas et al.	420136	?	?	unknown	brnQ/proY Score: 9.73	418847	421645	2798	+	11.06	10.65	0.00
pe11	Rivas et al.	420136	?	?	unknown	brnQ/proY Score: 9.73	419779	420187	408	-	9.56	9.35	0.00
C0200	Tjaden et al.	849580	849657	77	-	ybiF/ompX	849563	849627	64	-	10.16	10	0.00
C0369	Tjaden et al.	1568530	1568627	97	+	xasA/gadB	1567942	1568575	633	+	9.77	9.5	0.00
C0369	Tjaden et al.	1568530	1568627	97	+	xasA/gadB	1568581	1568643	62	+	10.05	9.72	0.01
C0365	Tjaden et al.	1553987	1554087	100	+	sfcA/yddX	1554062	1554139	77	+	10.04	9.71	0.01
C1069	Tjaden et al.	4531631	4531703	72	-	yjhQ/yjhR	4531263	4531764	501	-	9.87	9.78	0.00
C0209	Tjaden et al.	898873	899054	181	-	ybjF/artJ	898775	899873	1098	-	10.21	10	0.00
C0361	Tjaden et al.	1545201	1545329	128	+	yddG/fdnG	1545301	1545339	38	+	10.32	10.52	0.01
p25	Rivas et al.	887194	?	?	unknown	ybjK/ybjL Score: 6.15	886621	887253	632	+	9.68	9.61	0.00
tp27	Rivas et al.	9193	?	?	unknown	talB/mog Score: 11.06	8068	9246	1178	+	11.5	11.38	0.00
tp27	Rivas et al.	9193	?	?	unknown	talB/mog Score: 11.06	9192	9265	73	-	10.1	10.07	0.00
p27	Rivas et al.	434786	?	?	unknown	nusB/thiL Score: 5.96	432193	434842	2649	+	11.37	11.26	0.00
p27	Rivas et al.	434786	?	?	unknown	nusB/thiL Score: 5.96	434687	435160	473	-	9.79	9.49	0.00
tp25	Rivas et al.	1896332	?	?	unknown	sdaA/yoaD Score: 11.84	1896328	1896393	65	+	10.59	10.26	0.01
tp25	Rivas et al.	1896332	?	?	unknown	sdaA/yoaD Score: 11.84	1895873	1896609	736	-	10.29	10.23	0.00
IS225	Chen et al.	4569550	4569682	132	+	yjiS/yjiT	4569613	4569679	66	+	10.13	9.67	0.01
C0168	Tjaden et al.	712103	712177	74	-	ybfF/seqA	712097	714327	2230	-	9.95	9.79	0.00

tp21	Rivas et al.	1255837	?	?	unknown	ycgV/ychF Score: 13.34	1255821	1255876	55	+	10.17	9.8	0.01
IS140	Chen et al.	2816358	2816471	113	+	argY/argV	2815650	2816463	813	+	10.13	9.91	0.00
C0164	Tjaden et al.	705149	705271	122	-	nagE/glnS	704986	705216	230	-	10.39	9.81	0.01
IS142	Chen et al.	2885306	2885437	131	+	ygcB/cysH	2885370	2885499	129	+	9.89	9.7	0.00
IS143	Chen et al.	2925871	2926018	147	+	ygdH/sdaC	2925942	2925998	56	+	10.24	10.03	0.01
C0161	Tjaden et al.	696662	696695	33	+	metT/asnB	696629	698266	1637	+	10.01	9.75	0.00
p28	Rivas et al.	4421969	?	?	unknown	sgaE/yjfY Score: 5.58	4421147	4422226	1079	+	9.91	9.96	0.00
p28	Rivas et al.	4421969	?	?	unknown	sgaE/yjfY Score: 5.58	4421959	4422013	54	-	9.9	9.93	0.00
tp28	Rivas et al.	709961	?	?	unknown	fur/fldA Score: 10.62 Overlaps a 5' UTR	709909	710088	179	+	9.91	9.91	0.00
tp28	Rivas et al.	709961	?	?	unknown	fur/fldA Score: 10.62 Overlaps a 5' UTR	709354	709998	644	-	13.55	12.9	0.00
C0162	Tjaden et al.	698476	698787	311	+	asnB/nagD	698452	698567	115	+	10.11	9.95	0.00
C0162	Tjaden et al.	698476	698787	311	+	asnB/nagD	698577	698863	286	+	10.05	9.82	0.00
IS193	Chen et al.	3988483	3988583	100	+	hemC/cyaA HB_215 3988451-3988530 + Overlaps a 5' UTR	3988268	3991869	3601	+	12.49	12.18	0.00
C0832	Tjaden et al.	3571143	3571363	220	+	glgB/asd IS175 3571214-3571405 +	3569462	3571553	2091	+	9.6	9.43	0.00
IS191	Chen et al.	3912925	3913171	246	+	glmU/atpC	3912397	3913209	812	+	9.52	9.66	0.00
C0835	Tjaden et al.	3576479	3576551	72	+	gntR/yhhW HB_188 3576488-3576567 + HB_189 3576528-3576580 +	3576333	3576677	344	+	9.83	9.79	0.00
C0834	Tjaden et al.	3572520	3572616	96	+	asd/yhgN	3571676	3572944	1268	+	10.31	10.24	0.00
C1090	Tjaden et al.	4608861	4608963	102	-	prfC/osmY	4607679	4609099	1420	-	10.15	10.15	0.00
C0566	Tjaden et al.	2474407	2474539	132	+	yfdT/dsdC	2474411	2474505	94	+	13.68	12.86	0.01
C0566	Tjaden et al.	2474407	2474539	132	+	yfdT/dsdC	2474536	2474647	111	+	11.89	11.15	0.01
C0680	Tjaden et al.	2922341	2922536	195	+	yqcC/syd	2922349	2922391	42	+	9.64	9.76	0.01
C0680	Tjaden et al.	2922341	2922536	195	+	yqcC/syd	2922404	2922442	38	+	11.32	10.27	0.05
C0680	Tjaden et al.	2922341	2922536	195	+	yqcC/syd	2922467	2922534	67	+	11.32	11.09	0.01
C0687	Tjaden et al.	2974210	2974368	158	+	aas/galR HB_174 2974207-2974286 +	2974244	2974372	128	+	10.31	10.12	0.00
HB_210	Carter et al.	3930543	3930622	79	+	Not intergenic	3929145	3931073	1928	+	10.78	10.41	0.00
C0065	Tjaden et al.	236814	236897	83	+	dnaQ/aspV	235711	237059	1348	+	10.93	10.89	0.00
C0066	Tjaden et al.	237091	237297	206	+	aspV/yafT	237105	237152	47	+	11.17	10.51	0.02
C0066	Tjaden et al.	237091	237297	206	+	aspV/yafT	237260	237297	37	+	10.89	10.11	0.04
HB_211	Carter et al.	3939137	3939216	79	+	yieP/rrsC	3938508	3941444	2936	+	10.3	10.04	0.00
HB_280	Carter et al.	4577016	4577068	52	+	mcrB/yjiW HB_279 4576976-4577055 +	4576318	4577425	1107	+	10.01	9.83	0.00
IS194	Chen et al.	3998801	3998946	145	+	yigE/corA Overlaps a 5' UTR	3998809	3998867	58	+	9.43	9.23	0.01
IS194	Chen et al.	3998801	3998946	145	+	yigE/corA Overlaps a 5' UTR	3998928	3998968	40	+	10.44	10.31	0.00
HB_201	Carter et al.	3778725	3778791	66	+	lldD/yibK Overlaps a 3' UTR	3778770	3780653	1883	+	9.96	9.81	0.00
C1062	Tjaden et al.	4499430	4499669	239	+	yjgZ/yjhB	4499396	4499681	285	+	9.78	9.56	0.00
IS035	Chen et al.	812251	812372	121	+	bioD/uvrB HB_67 812291-812370 + HB_66 812251-812330 +	812273	812334	61	+	10.68	10.72	0.00
IS036	Chen et al.	899863	899959	96	+	artJ/artM IS037 899906-900056 +	899471	899873	402	+	9.8	9.45	0.01
IS036	Chen et al.	899863	899959	96	+	artJ/artM IS037 899906-900056 +	899957	900086	129	+	10.43	10.52	0.00
IS037	Chen et al.	899906	900056	150	+	artJ/artM IS036 899863-899959 +	899957	900086	129	+	10.43	10.52	0.00
IS030	Chen et al.	769959	770055	96	+	ybgG/cydA	769702	770031	329	+	9.92	9.94	0.00
IS031	Chen et al.	779607	779718	111	-	ybgF/lysT C0182 779628-779747 -	779298	780458	1160	-	10.27	10.03	0.00
IS033	Chen et al.	781029	781252	223	-	lysQ/nadA	780690	781099	409	-	10.16	9.91	0.00
IS033	Chen et al.	781029	781252	223	-	lysQ/nadA	781155	781222	67	-	10.36	10.28	0.00
IS033	Chen et al.	781029	781252	223	-	lysQ/nadA	781223	781262	39	-	10.86	10.16	0.03
C0703	Tjaden et al.	3069268	3069464	196	+	fbaA/pgk	3068232	3071674	3442	+	10.53	10.53	0.00
IS038	Chen et al.	915522	915655	133	+	aqpZ/ybjD	915523	917709	2186	+	10.24	10.17	0.00
C0906	Tjaden et al.	3882582	3882654	72	-	Not intergenic	3882406	3883013	607	-	10.17	9.88	0.00
IS139	Chen et al.	2796784	2796963	179	-	stpA/ygaW	2796855	2796898	43	-	9.83	9.61	0.01
C0900	Tjaden et al.	3851123	3851297	174	+	Not intergenic HB_204 3851106-3851185 +	3851128	3851207	79	+	9.84	9.79	0.00
p24	Rivas et al.	1886017	?	?	unknown	rnd/fadD Score: 6.42	1884780	1886137	1357	-	11.05	10.77	0.00
IS137	Chen et al.	2781499	2781595	96	-	pinH/ypjB	2781453	2781520	67	-	10.52	10.33	0.00
IS137	Chen et al.	2781499	2781595	96	-	pinH/ypjB	2781564	2781605	41	-	10.51	10.24	0.01
IS136	Chen et al.	2765455	2765720	265	+	yfjO/yfjP	2765434	2765470	36	+	10.68	10.38	0.01
IS136	Chen et al.	2765455	2765720	265	+	yfjO/yfjP	2765493	2765534	41	+	9.96	9.43	0.02
IS136	Chen et al.	2765455	2765720	265	+	yfjO/yfjP	2765665	2765708	43	+	10.44	10.27	0.01
C1061	Tjaden et al.	4498468	4498595	127	-	yjgX/yjgZ HB_548 4498538-4498617 -	4498020	4499072	1052	-	10.6	10.32	0.00
IS130	Chen et al.	2689279	2689628	349	+	yfhK/purL	2689225	2689298	73	+	10.65	10.07	0.01
IS130	Chen et al.	2689279	2689628	349	+	yfhK/purL	2689322	2689378	56	+	9.69	9.82	0.00
IS130	Chen et al.	2689279	2689628	349	+	yfhK/purL	2689424	2689471	47	+	9.93	9.24	0.03

IS130	Chen et al.	2689279	2689628	349	+	yfhK/purL	2689543	2689996	453	+	9.75	9.75	0.00
IS133	Chen et al.	2729285	2729623	338	+	rrsG/clpB t55 2729424	2727334	2729521	2187	+	10.99	10.5	0.02
IS133	Chen et al.	2729285	2729623	338	+	rrsG/clpB t55 2729424	2729581	2732142	2561	+	9.54	9.59	0.00
HB_218	Carter et al.	4025139	4025198	59	+	ubiB/fadA Overlaps a 3' UTR	4022955	4025705	2750	+	11.34	11.25	0.00
HB_278	Carter et al.	4566379	4566458	79	+	yjiO/yjiP	4565050	4566448	1398	+	9.87	9.84	0.00
HB_447	Carter et al.	3044028	3044107	79	-	ygfF/gcvP HB_446 3044068-3044147 - C0696 3043939-3044066 -	3043139	3047963	4824	-	11.32	11.37	0.00
HB_560	Carter et al.	4547362	4547441	79	-	fimH/gntP HB_559 4547402-4547481 -	4546766	4547636	870	-	9.76	9.39	0.01
HB_409	Carter et al.	2460667	2460741	74	-	fadL/yfdF IS117 2460675-2460836 -	2459360	2460700	1340	-	9.57	9.46	0.00
HB_408	Carter et al.	2459190	2459269	79	-	yfcZ/fadL	2459186	2459236	50	-	9.85	9.84	0.00
HB_408	Carter et al.	2459190	2459269	79	-	yfcZ/fadL	2459246	2459282	36	-	9.57	9.76	0.01
C0016	Tjaden et al.	49682	49780	98	+	kefC/folA	49658	49735	77	+	9.88	9.64	0.01
tpe92	Rivas et al.	2635420	?	?	unknown	ygfK/yfgL Score: 6.31 Resides within an operon	2633112	2641130	8018	-	11.78	11.52	0.00
HB_401	Carter et al.	2302528	2302607	79	-	eco/yojH HB_400 2302568-2302647 -	2302357	2302541	184	-	10.06	10.07	0.00
HB_400	Carter et al.	2302568	2302647	79	-	eco/yojH HB_401 2302528-2302607 -	2302646	2303930	1284	-	10.39	10.25	0.00
HB_403	Carter et al.	2391145	2391224	79	-	nuoM/nuoL Resides within an operon	2388148	2398218	10070	-	10.7	10.39	0.00
HB_402	Carter et al.	2302448	2302527	79	-	eco/yojH	2302357	2302541	184	-	10.06	10.07	0.00
HB_405	Carter et al.	2404931	2405010	79	-	lrhA/yfbQ Overlaps a 5' UTR	2404925	2405060	135	-	11.87	11.27	0.01
HB_404	Carter et al.	2405451	2405530	79	-	lrhA/yfbQ	2405444	2405521	77	-	9.67	9.42	0.01
HB_404	Carter et al.	2405451	2405530	79	-	lrhA/yfbQ	2405522	2406857	1335	-	9.97	9.93	0.00
HB_407	Carter et al.	2428962	2429041	79	-	cvpA/dedD Resides within an operon	2426094	2435943	9849	-	11.04	10.87	0.00
HB_406	Carter et al.	2411203	2411279	76	-	yfbV/ackA Overlaps a 5' UTR	2409478	2411394	1916	-	11.92	11.77	0.00
C0387	Tjaden et al.	1647224	1647351	127	-	rzpQ/dicB	1647246	1647407	161	-	9.88	9.82	0.00
C0386	Tjaden et al.	1644948	1645054	106	-	flxA/ydfW IS077 1644975-1645147 -	1644840	1645151	311	-	12.03	11.74	0.00
p26	Rivas et al.	2378544	?	?	unknown	Not intergenic C0536 2378534-2378740 + Score: 6.12 HB_135 2378529-2378608 +	2377336	2378691	1355	+	10.05	9.86	0.00
p26	Rivas et al.	2378544	?	?	unknown	Not intergenic C0536 2378534-2378740 + Score: 6.12 HB_135 2378529-2378608 +	2378091	2379592	1501	-	11.46	11.13	0.00
C0382	Tjaden et al.	1640115	1640303	188	-	cspF/ydfT	1639921	1640280	359	-	12.06	11.78	0.00
C0389	Tjaden et al.	1650738	1650900	162	-	intQ/rspB	1650779	1650852	73	-	9.76	9.71	0.00
C0389	Tjaden et al.	1650738	1650900	162	-	intQ/rspB	1650888	1651245	357	-	9.43	9.41	0.00
HB_383	Carter et al.	2163042	2163121	79	-	baeR/yegP	2163032	2163148	116	-	9.87	9.8	0.00
HB_492	Carter et al.	3706085	3706164	79	-	dppA/proK HB_491 3706125-3706204 -	3706131	3706186	55	-	10.3	10.16	0.00
HB_493	Carter et al.	3705965	3706044	79	-	dppA/proK HB_494 3705925-3706004 -	3705959	3705997	38	-	10.33	9.53	0.04
HB_493	Carter et al.	3705965	3706044	79	-	dppA/proK HB_494 3705925-3706004 -	3706015	3706056	41	-	10.7	10.62	0.00
HB_491	Carter et al.	3706125	3706204	79	-	dppA/proK HB_490 3706165-3706244 - HB_492 3706085-3706164 -	3706131	3706186	55	-	10.3	10.16	0.00
HB_496	Carter et al.	3723226	3723305	79	-	glyQ/yiaH C0877 3723150-3723337 -	3720538	3723442	2904	-	11.38	11.35	0.00
HB_497	Carter et al.	3737157	3737203	46	-	malS/avtA C0881 3737180-3737284 -	3737186	3737727	541	-	10.42	10.42	0.00
HB_385	Carter et al.	2165244	2165323	79	-	yegQ/ogrK	2165246	2165582	336	-	9.92	9.91	0.00
HB_495	Carter et al.	3719717	3719796	79	-	yiaZ/glyS tpke122 3719753	3719626	3719805	179	-	9.59	9.45	0.00
te12	Rivas et al.	3813399	?	?	unknown	pyrE/rph Score: 10.37	3813226	3813568	342	+	9.86	9.65	0.00
te12	Rivas et al.	3813399	?	?	unknown	pyrE/rph Score: 10.37	3813062	3813816	754	-	9.99	9.88	0.00
te10	Rivas et al.	1976232	?	?	unknown	flhD/yecG Score: 10.96	1975185	1976481	1296	-	11.73	11.78	0.00
te11	Rivas et al.	2111236	?	?	unknown	rfbB/galF Score: 10.73 Overlaps a 5' UTR	2111204	2111290	86	-	12.38	11.73	0.01
te16	Rivas et al.	1797175	?	?	unknown	pheS/pheM HB_363 1797170-1797249 - Score: 8.85 Resides within an operon	1790236	1797387	7151	-	11.17	11.04	0.00
te14	Rivas et al.	3161499	?	?	unknown	plsC/parC Score: 9.11	3161412	3161547	135	+	9.96	9.96	0.00
te14	Rivas et al.	3161499	?	?	unknown	plsC/parC Score: 9.11	3159186	3161598	2412	-	10.44	10.25	0.00
tk2	Rivas et al.	3604015	?	?	unknown	yhhN/zntA Score: 16.69	3603634	3604625	991	+	10.7	10.45	0.00
tk2	Rivas et al.	3604015	?	?	unknown	yhhN/zntA Score: 16.69	3603872	3604104	232	-	9.94	9.98	0.00
tpe34	Rivas et al.	136467	?	?	unknown	speE/yacC Score: 13.13	134763	136553	1790	-	11.37	11.25	0.00
C1073	Tjaden et al.	4540582	4540669	87	-	fimE/fimA	4540359	4540651	292	-	9.91	9.94	0.00
HB_268	Carter et al.	4516411	4516490	79	+	fecI/yjhU HB_269 4516451-4516530 + HB_267 4516371-4516450 +	4516333	4517332	999	+	11.76	10.93	0.01
t50	Rivas et al.	1755137	?	?	unknown	pykF/lpp Score: 7.50	1753467	1755184	1717	+	12.68	12.51	0.00
t50	Rivas et al.	1755137	?	?	unknown	pykF/lpp Score: 7.50	1753901	1755177	1276	-	10.97	10.87	0.00
t51	Rivas et al.	986207	?	?	unknown	ompF/asnS Score: 7.35	985074	986265	1191	+	10.9	10.92	0.00
t51	Rivas et al.	986207	?	?	unknown	ompF/asnS Score: 7.35	985074	986371	1297	-	13.55	13.66	0.00
t52	Rivas et al.	3071713	?	?	unknown	epd/yggC Score: 7.12 Overlaps a 5' UTR	3068150	3073698	5548	-	12.23	12.17	0.00
t54	Rivas et al.	1146775	?	?	unknown	rpmF/plsX Score: 7.03 Resides within an operon	1145826	1156894	11068	+	12.48	12.28	0.00

t54	Rivas et al.	1146775	?	?	unknown	rpmF/plsX Score: 7.03 Resides within an operon	1146370	1146804	434	-	10.67	10.66	0.00
t55	Rivas et al.	2729424	?	?	unknown	rrsG/clpB C0635 2729395-2729560 - Score: 7.00 IS133 2729285-2729623 +	2727334	2729521	2187	+	10.99	10.5	0.02
t55	Rivas et al.	2729424	?	?	unknown	rrsG/clpB C0635 2729395-2729560 - Score: 7.00 IS133 2729285-2729623 +	2727325	2729521	2196	-	11.5	10	0.04
t56	Rivas et al.	1121839	?	?	unknown	pyrC/yceB Score: 6.90	1121622	1121888	266	+	10.12	9.66	0.01
t56	Rivas et al.	1121839	?	?	unknown	pyrC/yceB Score: 6.90	1120757	1121888	1131	-	11.15	10.9	0.00
t57	Rivas et al.	4277420	?	?	unknown	yjcD/yjcE Score: 6.79	4276275	4277931	1656	+	10.8	10.71	0.00
HB_535	Carter et al.	4230863	4230926	63	-	lysC/pgi Overlaps a 5' UTR	4229870	4231553	1683	-	10.67	10.62	0.00
HB_534	Carter et al.	4230927	4231006	79	-	lysC/pgi HB_533 4230967-4231046 - Overlaps a 5' UTR	4229870	4231553	1683	-	10.67	10.62	0.00
HB_537	Carter et al.	4237310	4237373	63	-	yjbH/yjbA HB_536 4237334-4237413 -	4237247	4237319	72	-	9.89	9.88	0.00
HB_536	Carter et al.	4237334	4237413	79	-	yjbH/yjbA C0989 4237392-4237543 - HB_537 4237310-4237373 -	4237387	4237537	150	-	10.07	10.01	0.00
HB_531	Carter et al.	4212789	4212846	57	-	metA/aceB	4212646	4212841	195	-	9.63	9.63	0.00
HB_530	Carter et al.	4197689	4197768	79	-	yjaG/hupA	4197657	4197708	51	-	9.83	9.8	0.00
HB_530	Carter et al.	4197689	4197768	79	-	yjaG/hupA	4197713	4197949	236	-	9.98	9.92	0.00
HB_533	Carter et al.	4230967	4231046	79	-	lysC/pgi HB_532 4231007-4231086 - Overlaps a 5' UTR HB_534 4230927-4231006 -	4229870	4231553	1683	-	10.67	10.62	0.00
HB_532	Carter et al.	4231007	4231086	79	-	lysC/pgi HB_533 4230967-4231046 - Overlaps a 5' UTR	4229870	4231553	1683	-	10.67	10.62	0.00
HB_144	Carter et al.	2613992	2614063	71	+	yfgO/yfgC Overlaps a 5' UTR	2614007	2615323	1316	+	10.87	10.95	0.00
HB_249	Carter et al.	4323690	4323769	79	+	yjdN/yjdM HB_250 4323730-4323809 +	4323705	4323782	77	+	9.65	9.63	0.00
HB_440	Carter et al.	2875672	2875751	79	-	iap/ygbF	2875395	2875697	302	-	10.31	10.25	0.00
HB_440	Carter et al.	2875672	2875751	79	-	iap/ygbF	2875691	2876103	412	-	10.61	10.5	0.00
HB_538	Carter et al.	4242728	4242807	79	-	malF/malE Resides within an operon	4241034	4244311	3277	-	10.02	10.13	0.00
C1003	Tjaden et al.	4311835	4311917	82	+	rpiB/phnP	4311844	4313296	1452	+	10.45	10.36	0.00
HB_128	Carter et al.	2151681	2151760	79	+	yegL/yegM HB_127 2151641-2151720 +	2151636	2162957	11321	+	10.41	10.08	0.00
HB_129	Carter et al.	2245040	2245082	42	+	cirA/lysP	2245026	2245084	58	+	9.41	9.17	0.01
C0398	Tjaden et al.	1694211	1694352	141	+	uidA/uidR	1694179	1694258	79	+	9.96	9.82	0.00
p5	Rivas et al.	1823032	?	?	unknown	ydjR/spy Score: 16.94 Experimentally tested	1822991	1823062	71	+	9.76	9.64	0.00
p5	Rivas et al.	1823032	?	?	unknown	ydjR/spy Score: 16.94 Experimentally tested	1822960	1823069	109	-	10.53	10.29	0.00
HB_122	Carter et al.	2116637	2116701	64	+	wcaK/wzxC	2116444	2117077	633	+	10.22	10.11	0.00
HB_123	Carter et al.	2137548	2137627	79	+	yegH/asmA HB_124 2137588-2137667 +	2135941	2137743	1802	+	10.86	10.52	0.00
HB_120	Carter et al.	2087281	2087360	79	+	Not intergenic HB_119 2087241-2087320 +	2087235	2087402	167	+	9.79	9.79	0.00
HB_121	Carter et al.	2088109	2088163	54	+	hisL/hisG Overlaps a 5' UTR	2088077	2088185	108	+	13.42	13.25	0.00
HB_126	Carter et al.	2151601	2151680	79	+	yegL/yegM tpe60 2151623 HB_127 2151641-2151720 +	2151589	2151628	39	+	9.54	9.27	0.01
HB_126	Carter et al.	2151601	2151680	79	+	yegL/yegM tpe60 2151623 HB_127 2151641-2151720 +	2151636	2162957	11321	+	10.41	10.08	0.00
HB_127	Carter et al.	2151641	2151720	79	+	yegL/yegM HB_126 2151601-2151680 + HB_128 2151681-2151760 +	2151636	2162957	11321	+	10.41	10.08	0.00
HB_124	Carter et al.	2137588	2137667	79	+	yegH/asmA HB_123 2137548-2137627 +	2135941	2137743	1802	+	10.86	10.52	0.00
HB_125	Carter et al.	2151321	2151400	79	+	yegL/yegM IS105 2151195-2151378 +	2151275	2151589	314	+	10.12	9.9	0.00
HB_11	Carter et al.	183661	183708	47	+	yaeG/yaeH HB_10 183621-183700 +	182450	184094	1644	+	10.21	10.04	0.00
tpe43	Rivas et al.	451113	?	?	unknown	cyoA/ampG IS011 450965-451296 + Score: 11.80 Experimentally tested	451094	451164	70	+	10.39	10.34	0.00
tpe43	Rivas et al.	451113	?	?	unknown	cyoA/ampG IS011 450965-451296 + Score: 11.80 Experimentally tested	451094	451164	70	-	9.58	9.16	0.01
HB_17	Carter et al.	271520	271599	79	+	perR/ykfC HB_18 271560-271639 + Resides within an operon HB_16 271480-271559 +	269421	271808	2387	+	10.63	10.65	0.00
HB_16	Carter et al.	271480	271559	79	+	perR/ykfC HB_17 271520-271599 + Resides within an operon	269421	271808	2387	+	10.63	10.65	0.00
HB_14	Carter et al.	248295	248357	62	+	yafM/fhiA	248042	248772	730	+	10	10.02	0.00
HB_19	Carter et al.	271640	271719	79	+	perR/ykfC HB_20 271680-271759 + Resides within an operon	269421	271808	2387	+	10.63	10.65	0.00
HB_18	Carter et al.	271560	271639	79	+	perR/ykfC HB_17 271520-271599 + Resides within an operon	269421	271808	2387	+	10.63	10.65	0.00
C0950	Tjaden et al.	4103905	4103989	84	-	cpxP/yiiP HB_526 4103901-4103958 -	4101389	4104095	2706	-	10.82	10.77	0.00
C0248	Tjaden et al.	1017551	1017675	124	+	ycbZ/ycbG	1016618	1017677	1059	+	10.09	10.06	0.00
C0120	Tjaden et al.	507786	508062	276	-	ybaQ/ybaR HB_304 507819-507898 - HB_303 507939-508018 -	507437	510661	3224	-	10.47	9.87	0.01
C0125	Tjaden et al.	552356	552429	73	+	purE/ybbF	549847	552365	2518	+	10.15	10.01	0.00
C0125	Tjaden et al.	552356	552429	73	+	purE/ybbF	552374	552547	173	+	9.91	9.75	0.00
p2	Rivas et al.	4403566	?	?	unknown	purA/yjeB Score: 24.15 Experimentally testedResides within an operon	4390925	4404098	13173	+	11.61	11.39	0.00
p2	Rivas et al.	4403566	?	?	unknown	purA/yjeB Score: 24.15 Experimentally testedResides within an operon	4402817	4404073	1256	-	9.62	9.36	0.00
C0538	Tjaden et al.	2389373	2389530	157	+	nuoN/nuoM	2388017	2390931	2914	+	9.86	9.81	0.00

C0129	Tjaden et al.	576300	576615	315	+	nmpC/essD IS019 576356-576507 +	576302	576427	125	+	10.82	10.15	0.01
C0129	Tjaden et al.	576300	576615	315	+	nmpC/essD IS019 576356-576507 +	576432	577057	625	+	11.12	10.83	0.00
C0243	Tjaden et al.	989694	989812	118	+	pncB/pepN	989690	989728	38	+	11.2	10.8	0.02
C0243	Tjaden et al.	989694	989812	118	+	pncB/pepN	989729	992601	2872	+	11.41	11.36	0.00
C0246	Tjaden et al.	1014847	1014923	76	-	ymbA/rmf	1014836	1014882	46	-	10.2	9.65	0.02
C0246	Tjaden et al.	1014847	1014923	76	-	ymbA/rmf	1014892	1015070	178	-	10.6	10.63	0.00
C1088	Tjaden et al.	4603993	4604236	243	+	leuQ/rsmC HB_285 4604101-4604180 + HB_286 4604141-4604220 +	4603801	4604471	670	+	13.81	13.62	0.00
HB_561	Carter et al.	4608573	4608634	61	-	prfC/osmY t13 4608582	4607679	4609099	1420	-	10.15	10.15	0.00
pe9	Rivas et al.	3924637	?	?	unknown	asnC/asnA Score: 10.10	3924493	3924999	506	+	9.69	9.49	0.00
pe9	Rivas et al.	3924637	?	?	unknown	asnC/asnA Score: 10.10	3924532	3925055	523	-	10.18	9.93	0.00
C0879	Tjaden et al.	3733817	3733889	72	+	xylR/bax	3732959	3734670	1711	+	10.24	10.07	0.00
C0878	Tjaden et al.	3723282	3723377	95	+	glyQ/yiaH	3722997	3723610	613	+	10.26	10.06	0.00
C0877	Tjaden et al.	3723150	3723337	187	-	glyQ/yiaH HB_496 3723226-3723305 -	3720538	3723442	2904	-	11.38	11.35	0.00
tk1	Rivas et al.	3330379	?	?	unknown	yhbE/rpmA Score: 17.89 Experimentally tested	3330202	3330730	528	+	9.84	9.74	0.00
IS220	Chen et al.	4531472	4531746	274	+	yjhQ/yjhR HB_276 4531483-4531562 + tpke10 4531556	4531299	4531876	577	+	10.77	10.57	0.00
HB_236	Carter et al.	4197713	4197792	79	+	yjaG/hupA Resides within an operon	4197518	4197760	242	+	11.74	11.79	0.00
HB_236	Carter et al.	4197713	4197792	79	+	yjaG/hupA Resides within an operon	4197765	4198696	931	+	12.48	12.33	0.00
HB_234	Carter et al.	4177322	4177401	79	+	rplA/rplJ HB_235 4177362-4177441 + Resides within an operon	4176401	4187628	11227	+	13.04	12.78	0.00
HB_235	Carter et al.	4177362	4177441	79	+	rplA/rplJ HB_234 4177322-4177401 + Resides within an operon	4176401	4187628	11227	+	13.04	12.78	0.00
HB_233	Carter et al.	4177242	4177321	79	+	rplA/rplJ Resides within an operon	4176401	4187628	11227	+	13.04	12.78	0.00
HB_339	Carter et al.	1268572	1268651	79	-	kdsA/chaA HB_338 1268612-1268691 - HB_340 1268532-1268611 -	1268279	1269894	1615	-	10.22	10.03	0.01
HB_338	Carter et al.	1268612	1268691	79	-	kdsA/chaA HB_339 1268572-1268651 -	1268279	1269894	1615	-	10.22	10.03	0.01
HB_337	Carter et al.	1268852	1268931	79	-	kdsA/chaA HB_336 1268892-1268971 -	1268279	1269894	1615	-	10.22	10.03	0.01
HB_336	Carter et al.	1268892	1268971	79	-	kdsA/chaA HB_335 1268932-1269011 - HB_337 1268852-1268931 -	1268279	1269894	1615	-	10.22	10.03	0.01
HB_335	Carter et al.	1268932	1269011	79	-	kdsA/chaA HB_336 1268892-1268971 -	1268279	1269894	1615	-	10.22	10.03	0.01
HB_334	Carter et al.	1269092	1269171	79	-	kdsA/chaA HB_333 1269132-1269211 -	1268279	1269894	1615	-	10.22	10.03	0.01
HB_333	Carter et al.	1269132	1269211	79	-	kdsA/chaA HB_334 1269092-1269171 -	1268279	1269894	1615	-	10.22	10.03	0.01
HB_332	Carter et al.	1269412	1269491	79	-	kdsA/chaA HB_331 1269452-1269531 -	1268279	1269894	1615	-	10.22	10.03	0.01
HB_331	Carter et al.	1269452	1269531	79	-	kdsA/chaA HB_332 1269412-1269491 -	1268279	1269894	1615	-	10.22	10.03	0.01
HB_330	Carter et al.	1269612	1269691	79	-	kdsA/chaA HB_329 1269652-1269731 -	1268279	1269894	1615	-	10.22	10.03	0.01
tpk9	Rivas et al.	3474091	?	?	unknown	yheO/fkpA psrA17 3474078-3474144 - Score: 13.84 Resides within an operon	3473616	3474461	845	+	9.77	9.76	0.00
tpk9	Rivas et al.	3474091	?	?	unknown	yheO/fkpA psrA17 3474078-3474144 - Score: 13.84 Resides within an operon	3472755	3475556	2801	-	11.55	11.31	0.00
tpk8	Rivas et al.	2531402	?	?	unknown	cysK/ptsH Score: 14.45	2530366	2531435	1069	+	12.42	12.35	0.00
tpk8	Rivas et al.	2531402	?	?	unknown	cysK/ptsH Score: 14.45	2531366	2531510	144	-	10.59	10.07	0.01
C0401	Tjaden et al.	1702604	1702818	214	+	ydgJ/ydgT	1702438	1702914	476	+	12.17	11.54	0.00
C0406	Tjaden et al.	1735523	1735633	110	-	ydhP/purR	1735406	1735659	253	-	12.97	12.63	0.00
tpk3	Rivas et al.	4055641	?	?	unknown	glnA/typA Score: 24.68 Experimentally testedOverlaps a 5' UTR	4054942	4056164	1222	+	10.09	9.85	0.00
tpk3	Rivas et al.	4055641	?	?	unknown	glnA/typA Score: 24.68 Experimentally testedOverlaps a 5' UTR	4054410	4056164	1754	-	10.76	10.63	0.00
tpk4	Rivas et al.	47805	?	?	unknown	Not intergenic Score: 21.77 Experimentally tested	47694	47857	163	+	10.34	10.17	0.01
C0337	Tjaden et al.	1357334	1357442	108	-	ycjJ/ycjK HB_353 1357434-1357513 -	1357276	1357395	119	-	10.12	9.96	0.00
C0337	Tjaden et al.	1357334	1357442	108	-	ycjJ/ycjK HB_353 1357434-1357513 -	1357397	1357501	104	-	10.4	10	0.01
IS078	Chen et al.	1669041	1669213	172	-	ynfM/asr	1667880	1669161	1281	-	11.78	11.61	0.00
IS173	Chen et al.	3516932	3517022	90	-	aroK/hofQ tp48 3516988 Overlaps a 5' UTR	3510579	3517410	6831	-	11.85	11.7	0.00
tpe3	Rivas et al.	193445	?	?	unknown	frr/dxr Score: 23.88 Experimentally testedResides within an operon	189776	194806	5030	+	12.4	12.29	0.00
tpe3	Rivas et al.	193445	?	?	unknown	frr/dxr Score: 23.88 Experimentally testedResides within an operon	193391	194742	1351	-	9.83	9.6	0.00
C0238	Tjaden et al.	985039	985113	74	+	aspC/ompF	985074	986265	1191	+	10.9	10.92	0.00
tpe1	Rivas et al.	962930	?	?	unknown	rpsA/ihfB C0230 962916-963038 - Score: 24.75 Experimentally tested Resides within an operon	960353	963380	3027	+	12.88	12.76	0.00
tpe1	Rivas et al.	962930	?	?	unknown	rpsA/ihfB C0230 962916-963038 - Score: 24.75 Experimentally tested Resides within an operon	961033	963223	2190	-	10.35	10.27	0.00
IS070	Chen et al.	1553777	1554007	230	-	sfcA/yddX te3 1553785 Overlaps a 5' UTRResides within an operon	1553791	1554424	633	-	12.52	12.03	0.00
C0234	Tjaden et al.	980171	980267	96	-	mukB/ycbB	980157	980212	55	-	9.77	9.11	0.02
C0234	Tjaden et al.	980171	980267	96	-	mukB/ycbB	980245	980578	333	-	9.73	9.65	0.00
IS072	Chen et al.	1596191	1596326	135	+	ydeK/ydeV	1596203	1596244	41	+	10.44	10.45	0.00
C0236	Tjaden et al.	983535	983721	186	-	ycbL/aspC	983536	984969	1433	-	11.87	11.87	0.00
C0230	Tjaden et al.	962916	963038	122	-	rpsA/ihfB tpe1 962930	961033	963223	2190	-	10.35	10.27	0.00

C0233	Tjaden et al.	980151	980226	75	+	mukB/ycbB	980128	984931	4803	+	11.41	11.2	0.00
IS077	Chen et al.	1644975	1645147	172	-	flxA/ydfW C0386 1644948-1645054 -	1644840	1645151	311	-	12.03	11.74	0.00
k28	Rivas et al.	2742137	?	?	unknown	yfiB/rplS Score: 8.37	2741409	2742170	761	+	10.94	10.52	0.00
k28	Rivas et al.	2742137	?	?	unknown	yfiB/rplS Score: 8.37	2741950	2742186	236	-	10.51	10.09	0.01
k29	Rivas et al.	2528198	?	?	unknown	ligA/zipA Score: 8.14 Resides within an operon	2528015	2528304	289	+	10	9.87	0.00
k29	Rivas et al.	2528198	?	?	unknown	ligA/zipA Score: 8.14 Resides within an operon	2526158	2529437	3279	-	10.93	10.87	0.00
t25	Rivas et al.	960253	?	?	unknown	ycaL/cmk Score: 10.97	959990	960305	315	+	10.26	10.13	0.00
t25	Rivas et al.	960253	?	?	unknown	ycaL/cmk Score: 10.97	959661	960297	636	-	9.81	9.7	0.00
k20	Rivas et al.	2638654	?	?	unknown	hisS/gcpE Score: 10.65 Overlaps a 5' UTR Resides within an operon	2638618	2638696	78	+	9.59	9.53	0.00
k20	Rivas et al.	2638654	?	?	unknown	hisS/gcpE Score: 10.65 Overlaps a 5' UTR Resides within an operon	2633112	2641130	8018	-	11.78	11.52	0.00
k21	Rivas et al.	2265733	?	?	unknown	yeiQ/yeiR Score: 10.60	2265677	2265771	94	+	9.28	9.36	0.00
k21	Rivas et al.	2265733	?	?	unknown	yeiQ/yeiR Score: 10.60	2265179	2265818	639	-	10.2	10.23	0.00
k26	Rivas et al.	2995624	?	?	unknown	ygeO/ygeP Score: 9.41	2994378	2995735	1357	+	10.98	9.73	0.06
k26	Rivas et al.	2995624	?	?	unknown	ygeO/ygeP Score: 9.41	2994267	2995803	1536	-	9.57	9.35	0.00
C0192	Tjaden et al.	831471	831653	182	-	rhlE/ybiA	830548	831835	1287	-	10.11	9.98	0.00
k24	Rivas et al.	2157702	?	?	unknown	Not intergenic Score: 9.90	2151636	2162957	11321	+	10.41	10.08	0.00
k24	Rivas et al.	2157702	?	?	unknown	Not intergenic Score: 9.90	2157534	2158584	1050	-	10.92	10.54	0.01
k25	Rivas et al.	950305	?	?	unknown	pflA/pflB Score: 9.64	949652	950389	737	+	9.88	9.47	0.01
k25	Rivas et al.	950305	?	?	unknown	pflA/pflB Score: 9.64	950001	950378	377	-	12.62	12.02	0.01
HB_494	Carter et al.	3705925	3706004	79	-	dppA/proK HB_493 3705965-3706044 -	3705916	3705955	39	-	10.89	10.73	0.01
HB_494	Carter et al.	3705925	3706004	79	-	dppA/proK HB_493 3705965-3706044 -	3705959	3705997	38	-	10.33	9.53	0.04
p14	Rivas et al.	402834	?	?	unknown	psiF/yaiC Score: 9.44	402447	402874	427	+	11.49	11.2	0.00
HB_199	Carter et al.	3713960	3714039	79	+	bisC/yiaD	3713975	3714122	147	+	9.71	9.75	0.00
HB_198	Carter et al.	3705865	3705944	79	+	dppA/proK C0871 3705866-3705915 +	3705916	3706086	170	+	9.83	9.36	0.01
HB_193	Carter et al.	3606321	3606381	60	+	zntA/sirA HB_192 3606281-3606360 + Overlaps a 3' UTR	3604893	3607048	2155	+	10.09	10.04	0.00
HB_192	Carter et al.	3606281	3606360	79	+	zntA/sirA HB_193 3606321-3606381 + Overlaps a 3' UTR	3604893	3607048	2155	+	10.09	10.04	0.00
HB_191	Carter et al.	3598585	3598658	73	+	rpoH/ftsX	3598201	3599013	812	+	10.53	10.35	0.00
HB_190	Carter et al.	3578567	3578646	79	+	yhhX/yhhY	3578192	3578788	596	+	10.09	9.74	0.01
HB_197	Carter et al.	3679741	3679790	49	+	yhjJ/dctA	3679611	3679862	251	+	10.07	10.08	0.00
HB_196	Carter et al.	3679661	3679740	79	+	yhjJ/dctA	3679611	3679862	251	+	10.07	10.08	0.00
HB_195	Carter et al.	3661398	3661477	79	+	yhiV/yhiW C0861 3661470-3661506 +	3657354	3661583	4229	+	10.18	9.71	0.00
p18	Rivas et al.	1938219	?	?	unknown	msbB/yebA Score: 8.50	1937555	1938463	908	+	9.91	9.77	0.00
p18	Rivas et al.	1938219	?	?	unknown	msbB/yebA Score: 8.50	1937191	1938309	1118	-	10.47	10.33	0.00
C0751	Tjaden et al.	3305554	3305679	125	+	deaD/yhbM	3305197	3306678	1481	+	10.55	10.15	0.00
C0052	Tjaden et al.	192666	192850	184	-	pyrH/frr	192687	192842	155	-	9.39	9.41	0.00
C0052	Tjaden et al.	192666	192850	184	-	pyrH/frr	192844	193329	485	-	9.72	9.46	0.00
C0057	Tjaden et al.	200372	200470	98	-	yaeT/hlpA	200140	201570	1430	-	10.09	10.06	0.00
IS009	Chen et al.	302835	303034	199	-	yagU/ykgJ	302310	302853	543	-	10.08	10.16	0.00
IS009	Chen et al.	302835	303034	199	-	yagU/ykgJ	303018	303107	89	-	9.69	9.74	0.00
IS008	Chen et al.	289594	289856	262	+	argF/yagJ HB_29 289620-289699 +	289618	289869	251	+	10.53	10.56	0.00
tp6	Rivas et al.	3445788	?	?	unknown	rplN/rpsQ Score: 19.62 Experimentally tested Resides within an operon	3444190	3446279	2089	+	10.41	10.2	0.00
tp6	Rivas et al.	3445788	?	?	unknown	rplN/rpsQ Score: 19.62 Experimentally tested Resides within an operon	3437613	3451499	13886	-	13.48	13.29	0.00
IS005	Chen et al.	225589	225706	117	-	alaV/rrlH	223217	229162	5945	-	10.23	10.08	0.00
IS004	Chen et al.	191706	191797	91	-	tsf/pyrH tk7 191710 psrA1 191713-191793 -	190755	191791	1036	-	10.32	10.4	0.00
IS007	Chen et al.	284398	284523	125	-	yagF/yagG	283812	284445	633	-	9.7	9.46	0.00
IS118	Chen et al.	2468477	2468607	130	-	yfdI/tfaS	2468509	2468708	199	-	13.33	12.71	0.01
IS001	Chen et al.	20666	20798	132	+	nhaR/rpsT	20639	20697	58	+	10.14	9.65	0.02
IS001	Chen et al.	20666	20798	132	+	nhaR/rpsT	20740	20804	64	+	9.37	9.3	0.00
k1	Rivas et al.	3903244	?	?	unknown	bglF/bglG Score: 41.61 Experimentally tested	3902356	3903604	1248	+	10.64	9.87	0.01
k1	Rivas et al.	3903244	?	?	unknown	bglF/bglG Score: 41.61 Experimentally tested	3901909	3903654	1745	-	10.12	9.9	0.00
IS003	Chen et al.	167267	167427	160	-	mrcB/fhuA	167017	167427	410	-	11.86	11.44	0.00
C0894	Tjaden et al.	3809324	3809504	180	+	rpmB/radC	3809279	3809696	417	+	10.39	10.36	0.00
p29	Rivas et al.	4103904	?	?	unknown	cpxP/yiiP HB_526 4103901-4103958 - Score: 5.54	4101389	4104095	2706	-	10.82	10.77	0.00
tp4	Rivas et al.	4174709	?	?	unknown	tufB/secE Score: 21.48 Experimentally tested	4172793	4176370	3577	+	12.22	11.89	0.00
tp4	Rivas et al.	4174709	?	?	unknown	tufB/secE Score: 21.48 Experimentally tested	4173934	4175146	1212	-	10.85	10.98	0.00
C0757	Tjaden et al.	3315732	3315825	93	-	yhbC/metY	3315797	3315837	40	-	12.28	11.59	0.02
C0959	Tjaden et al.	4125999	4126105	106	-	metJ/metB	4125832	4127060	1228	-	11.49	11.37	0.00
C0955	Tjaden et al.	4113177	4113291	114	+	glpX/glpK	4113194	4113397	203	+	9.51	9.48	0.00

C0899	Tjaden et al.	3850423	3850515	92	+	ilvB/ivbL	3849695	3851037	1342	+	9.73	9.63	0.00
C0898	Tjaden et al.	3838060	3838143	83	+	nlpA/yicM	3838043	3838108	65	+	10.65	10.33	0.01
C0898	Tjaden et al.	3838060	3838143	83	+	nlpA/yicM	3838113	3838287	174	+	10.95	10.66	0.00
tpe79	Rivas et al.	75482	?	?	unknown	tbpA/yabN Score: 8.56	71378	75742	4364	-	10.1	9.87	0.00
HB_286	Carter et al.	4604141	4604220	79	+	leuQ/rsmC HB_285 4604101-4604180 + C1088 4603993-4604236 +	4603801	4604471	670	+	13.81	13.62	0.00
HB_285	Carter et al.	4604101	4604180	79	+	leuQ/rsmC HB_286 4604141-4604220 + C1088 4603993-4604236 +	4603801	4604471	670	+	13.81	13.62	0.00
HB_284	Carter et al.	4603363	4603442	79	+	Not intergenic	4601907	4603679	1772	+	10.48	10.24	0.00
HB_283	Carter et al.	4597182	4597261	79	+	mdoB/yjjA	4595522	4597484	1962	+	9.92	9.79	0.00
HB_282	Carter et al.	4594662	4594718	56	+	yjjN/mdoB	4594303	4595100	797	+	10.28	10.1	0.00
HB_281	Carter et al.	4580709	4580788	79	+	hsdM/hsdR	4579963	4581153	1190	+	9.9	9.77	0.00
tpe85	Rivas et al.	3044127	?	?	unknown	ygfF/gcvP HB_446 3044068-3044147 - Score: 7.44	3043139	3047963	4824	-	11.32	11.37	0.00
tpe75	Rivas et al.	2870845	?	?	unknown	ygbQ/ygbE Score: 9.09 Overlaps a 5' UTR	2870836	2870900	64	+	10.45	10.41	0.00
tpe75	Rivas et al.	2870845	?	?	unknown	ygbQ/ygbE Score: 9.09 Overlaps a 5' UTR	2866797	2871266	4469	-	11.15	11.04	0.00
tpe86	Rivas et al.	3598462	?	?	unknown	rpoH/ftsX C0840 3598435-3598507 + Score: 7.30 Overlaps a 5' UTR	3598201	3599013	812	+	10.53	10.35	0.00
HB_289	Carter et al.	164600	164679	79	-	hrpB/mrcB	164580	164649	69	-	10.56	10.5	0.00
HB_288	Carter et al.	34701	34780	79	-	caiF/caiE	34566	34886	320	-	11	10.94	0.00
HB_470	Carter et al.	3378225	3378287	62	-	yhcB/degQ	3378232	3378312	80	-	9.59	9.58	0.00
HB_471	Carter et al.	3388098	3388177	79	-	yhcS/tldD	3388113	3388167	54	-	10.01	9.85	0.01
HB_472	Carter et al.	3389974	3390053	79	-	tldD/yhdP Overlaps a 5' UTR	3388605	3390900	2295	-	11.63	11.53	0.00
HB_473	Carter et al.	3420899	3420978	79	-	yhdZ/rrfF HB_474 3420859-3420938 -	3420840	3420921	81	-	10.48	10.26	0.01
HB_474	Carter et al.	3420859	3420938	79	-	yhdZ/rrfF HB_473 3420899-3420978 -	3420840	3420921	81	-	10.48	10.26	0.01
HB_475	Carter et al.	3491387	3491437	50	-	yhfC/nirB	3491192	3491882	690	-	11	10.94	0.00
HB_476	Carter et al.	3502493	3502572	79	-	yhfR/yhfS HB_477 3502453-3502532 -	3501947	3502579	632	-	9.59	9.34	0.00
HB_477	Carter et al.	3502453	3502532	79	-	yhfR/yhfS HB_476 3502493-3502572 -	3501947	3502579	632	-	9.59	9.34	0.00
HB_479	Carter et al.	3540601	3540672	71	-	yhgG/yhgA	3538103	3541065	2962	-	10.2	10.1	0.00
HB_57	Carter et al.	719894	719973	79	+	Not intergenic HB_56 719854-719933 +	719890	719931	41	+	9.32	9.32	0.00
HB_56	Carter et al.	719854	719933	79	+	Not intergenic HB_57 719894-719973 +	719890	719931	41	+	9.32	9.32	0.00
HB_55	Carter et al.	685983	686061	78	+	gltJ/ybeJ	685908	686002	94	+	9.52	9.53	0.00
HB_55	Carter et al.	685983	686061	78	+	gltJ/ybeJ	686019	686960	941	+	10.02	9.74	0.00
HB_54	Carter et al.	661526	661601	75	+	lipB/ybeD	661522	661640	118	+	10	10.03	0.00
HB_53	Carter et al.	631223	631302	79	+	cstA/ybdD	629075	631993	2918	+	10.85	10.85	0.00
HB_52	Carter et al.	623824	623903	79	+	fepB/entC HB_51 623784-623863 +	623758	623927	169	+	10.63	10.71	0.00
HB_51	Carter et al.	623784	623863	79	+	fepB/entC HB_52 623824-623903 +	623758	623927	169	+	10.63	10.71	0.00
HB_50	Carter et al.	613283	613329	46	+	Not intergenic Resides within an operon	612099	617138	5039	+	11.57	11.89	0.00
tpe83	Rivas et al.	1269024	?	?	unknown	kdsA/chaA Score: 8.09 Overlaps a 3' UTR	1262830	1269867	7037	+	11.69	11.38	0.00
tpe83	Rivas et al.	1269024	?	?	unknown	kdsA/chaA Score: 8.09 Overlaps a 3' UTR	1268279	1269894	1615	-	10.22	10.03	0.01
tpe88	Rivas et al.	16979	?	?	unknown	gefL/nhaA Score: 6.95	16967	17076	109	+	10.5	9.74	0.02
tpe88	Rivas et al.	16979	?	?	unknown	gefL/nhaA Score: 6.95	16429	17146	717	-	10.93	10.59	0.00
C0853	Tjaden et al.	3652278	3652310	32	-	slp/yhiF	3652171	3652374	203	-	10.11	9.71	0.01
HB_59	Carter et al.	754262	754341	79	+	gltA/sdhC HB_60 754302-754349 + Overlaps a 5' UTR	754259	754333	74	+	10.21	9.96	0.01
HB_58	Carter et al.	753782	753861	79	+	gltA/sdhC	753789	753847	58	+	10.15	10.17	0.00
k44	Rivas et al.	3376288	?	?	unknown	rplM/yhcM Score: 6.34	3375945	3376799	854	+	10.64	10.47	0.00
k44	Rivas et al.	3376288	?	?	unknown	rplM/yhcM Score: 6.34	3375837	3377962	2125	-	12.29	12.05	0.00
IS094	Chen et al.	1994968	1995087	119	+	sdiA/yecC HB_115 1995026-1995084 +	1994934	1994991	57	+	11.36	10.35	0.03
IS094	Chen et al.	1994968	1995087	119	+	sdiA/yecC HB_115 1995026-1995084 +	1994996	1995978	982	+	10.57	10.34	0.00
C0967	Tjaden et al.	4173437	4173516	79	-	thrT/tufB	4173444	4173923	479	-	10.47	10.39	0.00
HB_522	Carter et al.	4054125	4054204	79	-	glnL/glnA HB_523 4054085-4054164 - Overlaps a 3' UTR	4051444	4054400	2956	-	11.28	11.19	0.00
C0807	Tjaden et al.	3472189	3472298	109	+	rpsL/yheL t21 3472191	3472157	3472634	477	+	10.66	10.46	0.00
tp11	Rivas et al.	2151247	?	?	unknown	yegL/yegM IS105 2151195-2151378 + Score: 15.88	2151207	2151265	58	+	9.98	9.88	0.00
tp11	Rivas et al.	2151247	?	?	unknown	yegL/yegM IS105 2151195-2151378 + Score: 15.88	2151225	2151276	51	-	9.71	9.43	0.01
IS098	Chen et al.	2041113	2041206	93	+	yodB/serU	2041106	2041153	47	+	10.33	9.96	0.01
IS098	Chen et al.	2041113	2041206	93	+	yodB/serU	2041170	2041228	58	+	10.17	9.8	0.01
C0479	Tjaden et al.	2033664	2033850	186	+	yedS/yedU	2033653	2033707	54	+	11.55	11.51	0.00
C0479	Tjaden et al.	2033664	2033850	186	+	yedS/yedU	2033716	2033913	197	+	10.1	10.23	0.00
C0478	Tjaden et al.	2033313	2033503	190	-	yedS/yedU	2033301	2033463	162	-	10.07	9.96	0.00
IS209	Chen et al.	4258973	4259131	158	+	yjbM/yjbN	4259064	4259234	170	+	11.48	11.02	0.01
C0984	Tjaden et al.	4215995	4216167	172	-	aceA/aceK	4213437	4216206	2769	-	10.05	9.91	0.00
tp17	Rivas et al.	4113161	?	?	unknown	glpX/glpK Score: 14.22	4112540	4113596	1056	-	10.77	10.71	0.00

C0476	Tjaden et al.	2026113	2026208	95	+	yedQ/yodC	2026015	2026305	290	+	9.93	9.61	0.00
tp16	Rivas et al.	3344103	?	?	unknown	yhbH/ptsN Score: 14.79	3339738	3345039	5301	+	11.58	11.54	0.00
tp16	Rivas et al.	3344103	?	?	unknown	yhbH/ptsN Score: 14.79	3342422	3345039	2617	-	10.16	10.05	0.00
HB_373	Carter et al.	2066340	2066419	79	-	yeeH/yoeA HB_372 2066380-2066459 -	2066260	2066361	101	-	12.14	11.82	0.01
HB_373	Carter et al.	2066340	2066419	79	-	yeeH/yoeA HB_372 2066380-2066459 -	2066365	2066514	149	-	11.18	10.96	0.00
HB_372	Carter et al.	2066380	2066459	79	-	yeeH/yoeA HB_373 2066340-2066419 -	2066365	2066514	149	-	11.18	10.96	0.00
HB_371	Carter et al.	2041170	2041249	79	-	yodB/serU	2041122	2041283	161	-	10.45	10.19	0.00
HB_370	Carter et al.	1993511	1993590	79	-	uvrY/yecF	1993422	1993635	213	-	11.06	10.84	0.00
HB_377	Carter et al.	2116502	2116581	79	-	wcaK/wzxC HB_378 2116477-2116541 - C0496 2116472-2116688 -	2116464	2117857	1393	-	10.06	9.96	0.00
HB_376	Carter et al.	2116622	2116701	79	-	wcaK/wzxC C0496 2116472-2116688 -	2116464	2117857	1393	-	10.06	9.96	0.00
HB_375	Carter et al.	2087284	2087363	79	-	Not intergenic	2087226	2087402	176	-	10.83	10.8	0.00
HB_374	Carter et al.	2076254	2076333	79	-	yeeW/yoeD C0487 2076255-2076327 -	2076231	2076278	47	-	9.95	9.91	0.00
HB_374	Carter et al.	2076254	2076333	79	-	yeeW/yoeD C0487 2076255-2076327 -	2076279	2076335	56	-	9.84	9.81	0.00
HB_499	Carter et al.	3748412	3748477	65	-	sgbE/yiaT	3746241	3748844	2603	-	10.34	10.14	0.00
HB_379	Carter et al.	2151761	2151840	79	-	yegL/yegM HB_380 2151721-2151800 -	2151763	2151871	108	-	10.66	10.47	0.00
HB_378	Carter et al.	2116477	2116541	64	-	wcaK/wzxC HB_377 2116502-2116581 - C0496 2116472-2116688 -	2116464	2117857	1393	-	10.06	9.96	0.00
HB_175	Carter et al.	3040394	3040473	79	+	ygfZ/yqfA Overlaps a 3' UTR	3039300	3041127	1827	+	10.62	10.56	0.00
HB_174	Carter et al.	2974207	2974286	79	+	aas/galR C0687 2974210-2974368 +	2974244	2974372	128	+	10.31	10.12	0.00
C0623	Tjaden et al.	2693796	2693945	149	+	purL/yfhD	2693775	2693821	46	+	10.81	10.65	0.01
C0623	Tjaden et al.	2693796	2693945	149	+	purL/yfhD	2693842	2693900	58	+	10.33	10.07	0.01
C0623	Tjaden et al.	2693796	2693945	149	+	purL/yfhD	2693905	2695601	1696	+	9.82	9.77	0.00
IS204	Chen et al.	4174729	4174842	113	-	tufB/secE	4173934	4175146	1212	-	10.85	10.98	0.00
HB_171	Carter et al.	2902277	2902356	79	+	ygcE/ygcF	2902142	2902440	298	+	9.88	9.61	0.00
HB_170	Carter et al.	2901397	2901476	79	+	ygcE/ygcF	2899802	2901439	1637	+	10.02	9.98	0.00
HB_170	Carter et al.	2901397	2901476	79	+	ygcE/ygcF	2901438	2901484	46	+	9.63	9.59	0.00
HB_173	Carter et al.	2964110	2964189	79	+	lgt/ptsP	2964089	2965725	1636	+	9.96	9.8	0.00
HB_172	Carter et al.	2962290	2962369	79	+	ppdA/thyA	2962267	2962914	647	+	9.89	9.73	0.00
HB_272	Carter et al.	4516571	4516650	79	+	fecI/yjhU HB_271 4516531-4516610 +	4516333	4517332	999	+	11.76	10.93	0.01
C1012	Tjaden et al.	4350096	4350123	27	+	yjdJ/yjdK	4349720	4350517	797	+	11.57	11.27	0.00
HB_270	Carter et al.	4516491	4516570	79	+	fecI/yjhU HB_271 4516531-4516610 + HB_269 4516451-4516530 +	4516333	4517332	999	+	11.76	10.93	0.01
HB_229	Carter et al.	4160980	4161059	79	+	trmA/btuB HB_228 4160940-4161019 + Overlaps a 5' UTR	4159063	4161360	2297	+	11.05	10.85	0.00
HB_179	Carter et al.	3192702	3192781	79	+	yqiK/rfaE HB_180 3192742-3192821 + HB_178 3192662-3192741 +	3192640	3192783	143	+	10.27	10.1	0.00
HB_178	Carter et al.	3192662	3192741	79	+	yqiK/rfaE HB_179 3192702-3192781 +	3192640	3192783	143	+	10.27	10.1	0.00
HB_274	Carter et al.	4518197	4518238	41	+	yjhU/yjhF C1066 4518187-4518237 + HB_273 4518157-4518236 +	4518185	4518253	68	+	9.56	9.56	0.00
HB_275	Carter et al.	4527862	4527941	79	+	Not intergenic	4527764	4527945	181	+	9.49	9.43	0.00
t17	Rivas et al.	1860602	?	?	unknown	yeaA/gapA Score: 12.85 Overlaps a 5' UTR	1860583	1862663	2080	+	12.71	12.54	0.00
t10	Rivas et al.	3468968	?	?	unknown	tufA/fusA Score: 15.42 Resides within an operon	3468169	3472147	3978	+	11.04	11.15	0.00
t10	Rivas et al.	3468968	?	?	unknown	tufA/fusA Score: 15.42 Resides within an operon	3468097	3472677	4580	-	13.47	13.4	0.00
t11	Rivas et al.	2815884	?	?	unknown	argQ/argZ Score: 14.64	2815650	2816463	813	+	10.13	9.91	0.00
t11	Rivas et al.	2815884	?	?	unknown	argQ/argZ Score: 14.64	2815658	2816758	1100	-	13.73	13.37	0.00
t12	Rivas et al.	2268577	?	?	unknown	spr/rtn Score: 14.11	2267831	2268615	784	+	14.33	14.05	0.00
t12	Rivas et al.	2268577	?	?	unknown	spr/rtn Score: 14.11	2268253	2268608	355	-	10.11	9.98	0.00
C1048	Tjaden et al.	4457950	4458034	84	+	nrdG/nrdD	4457923	4457984	61	+	9.73	9.49	0.01
IS200	Chen et al.	4076525	4076636	111	-	yiiD/yiiE	4076558	4076606	48	-	9.08	9.06	0.00
t18	Rivas et al.	3375331	?	?	unknown	sspA/rpsI Score: 12.63	3375161	3375442	281	+	10.01	10.01	0.00
t18	Rivas et al.	3375331	?	?	unknown	sspA/rpsI Score: 12.63	3374007	3375543	1536	-	11.98	11.73	0.00
t19	Rivas et al.	4407596	?	?	unknown	yjfH/yjfI Score: 12.50	4404715	4408012	3297	+	10.61	10.45	0.00
t19	Rivas et al.	4407596	?	?	unknown	yjfH/yjfI Score: 12.50	4407262	4407986	724	-	9.49	9.15	0.00
IS149	Chen et al.	3048693	3048953	260	-	gcvT/visC HB_448 3048738-3048814 - Overlaps a 5' UTR	3048107	3048832	725	-	12.19	12.38	0.00
IS149	Chen et al.	3048693	3048953	260	-	gcvT/visC HB_448 3048738-3048814 - Overlaps a 5' UTR	3048861	3048908	47	-	10.46	9.82	0.02
C0272	Tjaden et al.	1112684	1112759	75	+	mdoH/yceK	1108386	1112773	4387	+	11	10.63	0.00
HB_411	Carter et al.	2525981	2526060	79	-	Not intergenic HB_410 2526021-2526100 -	2524927	2526131	1204	-	9.87	9.79	0.00
C0276	Tjaden et al.	1124608	1124743	135	+	yceL/rimJ	1124692	1124739	47	+	10.23	10.46	0.01
HB_227	Carter et al.	4150968	4151047	79	+	ppc/argE	4148366	4151043	2677	+	10.24	10.17	0.00
IS212	Chen et al.	4359749	4360129	380	+	cadC/pheU HB_257 4360043-4360122 +	4359862	4359993	131	+	9.31	9.15	0.01
C0372	Tjaden et al.	1609916	1609988	72	+	yneF/yneG	1609836	1610041	205	+	12.23	11.58	0.01
tp31	Rivas et al.	3301012	?	?	unknown	yhbV/yhbW Score: 9.75	3299421	3301389	1968	+	10.06	10.01	0.00
tp31	Rivas et al.	3301012	?	?	unknown	yhbV/yhbW Score: 9.75	3299481	3301273	1792	-	9.69	9.49	0.00

tp30	Rivas et al.	1975165	?	?	unknown	motA/flhC Score: 9.75	1970815	1975193	4378	-	13.05	12.82	0.00
C0826	Tjaden et al.	3534246	3534378	132	-	ompR/greB	3532513	3534632	2119	-	10.68	10.71	0.00
tp35	Rivas et al.	2430968	?	?	unknown	folC/accD Score: 8.74	2430622	2431020	398	+	9.64	9.38	0.01
tp35	Rivas et al.	2430968	?	?	unknown	folC/accD Score: 8.74	2426094	2435943	9849	-	11.04	10.87	0.00
tp37	Rivas et al.	3534929	?	?	unknown	greB/yhgF HB_478 3534927-3534991 - Score: 8.68	3534771	3537766	2995	+	10.77	10.79	0.00
p32	Rivas et al.	2465734	?	?	unknown	intS/yfdG Score: 5.03	2464503	2465749	1246	+	10.73	10.54	0.00
p30	Rivas et al.	3769619	?	?	unknown	yibI/mtlA Score: 5.37	3769579	3769655	76	+	9.82	9.87	0.00
p30	Rivas et al.	3769619	?	?	unknown	yibI/mtlA Score: 5.37	3769434	3769811	377	-	10.08	9.69	0.01
C0175	Tjaden et al.	761963	762219	256	-	sucB/sucC	761824	762180	356	-	9.61	9.59	0.00
C0175	Tjaden et al.	761963	762219	256	-	sucB/sucC	762203	762324	121	-	9.2	9.24	0.00
C0171	Tjaden et al.	740155	740296	141	-	phrB/ybgH	740149	741895	1746	-	10.23	9.88	0.00
HB_306	Carter et al.	572969	573048	79	-	Not intergenic	572972	573039	67	-	10.38	10.51	0.00
HB_307	Carter et al.	585290	585369	79	-	ompT/envY	585276	585911	635	-	9.95	9.92	0.00
HB_304	Carter et al.	507819	507898	79	-	ybaQ/ybaR C0120 507786-508062 -	507437	510661	3224	-	10.47	9.87	0.01
HB_305	Carter et al.	550556	550629	73	-	ybcF/purK Overlaps a 3' UTR	550446	554375	3929	-	11.92	11.82	0.00
pe8	Rivas et al.	58182	?	?	unknown	djlA/yabP Score: 10.13	57193	58514	1321	+	10.8	10.46	0.00
pe8	Rivas et al.	58182	?	?	unknown	djlA/yabP Score: 10.13	58017	58251	234	-	9.9	9.84	0.00
HB_303	Carter et al.	507939	508018	79	-	ybaQ/ybaR C0120 507786-508062 -	507437	510661	3224	-	10.47	9.87	0.01
HB_301	Carter et al.	421609	421688	79	-	proY/malZ HB_302 421584-421648 -	421504	421617	113	-	9.3	9.29	0.00
HB_301	Carter et al.	421609	421688	79	-	proY/malZ HB_302 421584-421648 -	421629	421679	50	-	10.3	10.27	0.00
pe4	Rivas et al.	4566122	?	?	unknown	yjiO/yjiP Score: 11.58	4565050	4566448	1398	+	9.87	9.84	0.00
pe4	Rivas et al.	4566122	?	?	unknown	yjiO/yjiP Score: 11.58	4566009	4566596	587	-	9.92	9.58	0.01
tpke10	Rivas et al.	4531556	?	?	unknown	yjhQ/yjhR HB_276 4531483-4531562 + Score: 19.66<br>IS220 4531472-4531746 + Experimentally tested	4531299	4531876	577	+	10.77	10.57	0.00
tpke10	Rivas et al.	4531556	?	?	unknown	yjhQ/yjhR HB_276 4531483-4531562 + Score: 19.66<br>IS220 4531472-4531746 + Experimentally tested	4531263	4531764	501	-	9.87	9.78	0.00
HB_308	Carter et al.	596224	596303	79	-	ylcB/ylcC	596049	597155	1106	-	9.62	9.45	0.00
HB_309	Carter et al.	606958	607037	79	-	ybdK/ybdY	606895	607100	205	-	10.36	10.03	0.00
C0573	Tjaden et al.	2515973	2516058	85	+	yfeA/alaX	2515867	2516081	214	+	10.35	10.27	0.00
C0570	Tjaden et al.	2508924	2509014	90	-	yfeO/yfeP	2508909	2510794	1885	-	11.9	11.52	0.00
C0571	Tjaden et al.	2508935	2509019	84	+	yfeO/yfeP	2508933	2510607	1674	+	10	9.95	0.00
C0575	Tjaden et al.	2526001	2526073	72	+	Not intergenic	2524918	2526161	1243	+	10.52	10.46	0.00
t7	Rivas et al.	4525553	?	?	unknown	sgcA/sgcQ Score: 19.11 Experimentally tested	4525536	4525846	310	+	10.47	10.44	0.00
t7	Rivas et al.	4525553	?	?	unknown	sgcA/sgcQ Score: 19.11 Experimentally tested	4525137	4526057	920	-	9.88	9.82	0.00
C0579	Tjaden et al.	2531669	2531767	98	-	cysK/ptsH	2531624	2534126	2502	-	10.56	10.4	0.00
C1078	Tjaden et al.	4566160	4566320	160	-	yjiO/yjiP IS224 4566199-4566339 -	4566009	4566596	587	-	9.92	9.58	0.01
HB_228	Carter et al.	4160940	4161019	79	+	trmA/btuB HB_229 4160980-4161059 + Overlaps a 5' UTR	4159063	4161360	2297	+	11.05	10.85	0.00
IS087	Chen et al.	1887856	1887966	110	+	fadD/yeaY psrA7 1887849-1887959 +	1887920	1887956	36	+	9.54	9.53	0.00
C0095	Tjaden et al.	440604	440652	48	-	xseB/thiI	436303	441028	4725	-	10.82	10.7	0.00
C0090	Tjaden et al.	408255	408327	72	+	ykiA/yaiD	408253	408331	78	+	10.4	10.26	0.00
IS043	Chen et al.	1050440	1050555	115	-	cspH/cspG C0258 1050518-1050678 -	1050449	1050489	40	-	9.38	9.23	0.01
IS042	Chen et al.	1014724	1014910	186	+	ymbA/rmf	1014707	1014766	59	+	10.27	10.07	0.01
IS042	Chen et al.	1014724	1014910	186	+	ymbA/rmf	1014779	1014882	103	+	11.07	10.88	0.00
IS042	Chen et al.	1014724	1014910	186	+	ymbA/rmf	1014878	1015742	864	+	11.83	11.49	0.00
IS045	Chen et al.	1102640	1103008	368	+	csgD/csgB	1102877	1102916	39	+	9.99	9.99	0.00
C0655	Tjaden et al.	2806169	2806300	131	-	proX/ygaY	2802882	2806297	3415	-	10.27	10.06	0.00
C0305	Tjaden et al.	1260061	1260149	88	+	ychM/prs	1260058	1260101	43	+	10.12	9.98	0.01
C0305	Tjaden et al.	1260061	1260149	88	+	ychM/prs	1260102	1260329	227	+	9.43	9.39	0.00
C0073	Tjaden et al.	259483	259571	88	+	phoE/proB	259464	262049	2585	+	10.96	10.91	0.00
C0307	Tjaden et al.	1261103	1261247	144	+	prs/ychB	1260913	1262679	1766	+	10.19	9.94	0.00
C1102	Tjaden et al.	4638131	4638203	72	-	yjjY/lasT	4637330	4638388	1058	-	12.2	12.14	0.00
C1029	Tjaden et al.	4406683	4406845	162	-	rnr/yjfH	4405801	4406708	907	-	9.92	9.9	0.00
C0300	Tjaden et al.	1232303	1232384	81	-	dsbB/nhaB	1232283	1232382	99	-	10.17	9.82	0.01
tp44	Rivas et al.	2735517	?	?	unknown	yfiA/pheL Score: 7.22	2734081	2736952	2871	+	12.41	12.36	0.00
tp44	Rivas et al.	2735517	?	?	unknown	yfiA/pheL Score: 7.22	2735433	2735591	158	-	10.14	9.72	0.01
k12	Rivas et al.	3934798	?	?	unknown	rbsB/rbsK Score: 12.04	3933773	3937912	4139	+	11.05	10.96	0.00
tp46	Rivas et al.	3679595	?	?	unknown	yhjJ/dctA Score: 6.96	3676110	3680027	3917	-	11.65	11.46	0.00
tp47	Rivas et al.	418413	?	?	unknown	phoR/brnQ Score: 6.71	418266	418434	168	-	10.37	9.92	0.02

k17	Rivas et al.	3244180	?	?	unknown	exuT/exuR Score: 11.21	3243451	3244543	1092	+	9.94	9.73	0.00
k17	Rivas et al.	3244180	?	?	unknown	exuT/exuR Score: 11.21	3243602	3244633	1031	-	10.21	10.1	0.00
k15	Rivas et al.	261982	?	?	unknown	proA/thrW Score: 11.33	259464	262049	2585	+	10.96	10.91	0.00
k15	Rivas et al.	261982	?	?	unknown	proA/thrW Score: 11.33	261718	262070	352	-	10.46	10.12	0.01
IS129	Chen et al.	2651687	2652078	391	-	sseA/sseB HB_418 2651857-2651936 -	2651717	2651848	131	-	12.03	11.41	0.01
IS129	Chen et al.	2651687	2652078	391	-	sseA/sseB HB_418 2651857-2651936 -	2651859	2652042	183	-	10.1	9.71	0.01
psrA2	Argaman et al.	454066	454262	196	-	bolA/tig IS012 454059-454263 -	454056	454136	80	-	10.02	9.89	0.00
psrA2	Argaman et al.	454066	454262	196	-	bolA/tig IS012 454059-454263 -	454141	454242	101	-	9.95	9.69	0.01
IS122	Chen et al.	2496319	2496526	207	-	yfdZ/ypdA	2495127	2496374	1247	-	10.75	10.8	0.00
IS122	Chen et al.	2496319	2496526	207	-	yfdZ/ypdA	2496386	2496434	48	-	11.16	10.46	0.02
IS122	Chen et al.	2496319	2496526	207	-	yfdZ/ypdA	2496437	2496503	66	-	10.83	9.8	0.02
HB_490	Carter et al.	3706165	3706244	79	-	dppA/proK HB_491 3706125-3706204 -	3706131	3706186	55	-	10.3	10.16	0.00
HB_258	Carter et al.	4368161	4368215	54	+	yjeH/groS Overlaps a 5' UTR	4366802	4368216	1414	+	11.14	10.9	0.00
C0781	Tjaden et al.	3381939	3382059	120	+	mdh/argR	3382027	3382228	201	+	10.39	9.84	0.02
C0782	Tjaden et al.	3382808	3382886	78	-	argR/yhcN	3382866	3382992	126	-	10.46	10.37	0.00
p1	Rivas et al.	4049438	?	?	unknown	yihI/hemN HB_521 4049437-4049488 - Score: 25.68<br>Experimentally tested	4049313	4051742	2429	+	10.86	10.64	0.00
C0624	Tjaden et al.	2693860	2693932	72	-	purL/yfhD	2693842	2694576	734	-	10.41	10.4	0.00
C0945	Tjaden et al.	4077774	4077861	87	+	yiiF/fdhE HB_224 4077790-4077869 +	4077760	4077991	231	+	9.78	9.44	0.01
HB_261	Carter et al.	4456966	4457045	79	+	pmbA/cybC HB_260 4456926-4457005 +<br>Resides within an operon	4456002	4457395	1393	+	11.12	10.93	0.00
HB_434	Carter et al.	2876272	2876351	79	-	iap/ygbF IS141 2876310-2876479 - HB_433 2876312-2876391 -<br>HB_435 2876232-2876311 -	2876139	2876496	357	-	10.6	10.38	0.00
HB_435	Carter et al.	2876232	2876311	79	-	iap/ygbF HB_434 2876272-2876351 - IS141 2876310-2876479 -	2876139	2876496	357	-	10.6	10.38	0.00
HB_436	Carter et al.	2876072	2876151	79	-	iap/ygbF	2875691	2876103	412	-	10.61	10.5	0.00
HB_436	Carter et al.	2876072	2876151	79	-	iap/ygbF	2876139	2876496	357	-	10.6	10.38	0.00
HB_437	Carter et al.	2875872	2875951	79	-	iap/ygbF HB_438 2875832-2875911 -	2875691	2876103	412	-	10.61	10.5	0.00
HB_430	Carter et al.	2816375	2816454	79	-	argY/argV	2815658	2816758	1100	-	13.73	13.37	0.00
HB_431	Carter et al.	2823650	2823724	74	-	mltB/srlA	2822474	2823685	1211	-	10.34	10.15	0.00
HB_432	Carter et al.	2864502	2864581	79	-	ygbN/rpoS	2863098	2864527	1429	-	9.73	9.48	0.00
HB_432	Carter et al.	2864502	2864581	79	-	ygbN/rpoS	2864534	2866800	2266	-	11.93	11.95	0.00
HB_433	Carter et al.	2876312	2876391	79	-	iap/ygbF IS141 2876310-2876479 - HB_434 2876272-2876351 -	2876139	2876496	357	-	10.6	10.38	0.00
HB_438	Carter et al.	2875832	2875911	79	-	iap/ygbF HB_437 2875872-2875951 - HB_439 2875792-2875871 -	2875691	2876103	412	-	10.61	10.5	0.00
HB_439	Carter et al.	2875792	2875871	79	-	iap/ygbF HB_438 2875832-2875911 -	2875691	2876103	412	-	10.61	10.5	0.00
HB_457	Carter et al.	3182546	3182625	79	-	ribB/yqiC HB_456 3182586-3182665 - Overlaps a 5' UTR	3181731	3182750	1019	-	12.54	12.4	0.00
HB_226	Carter et al.	4145947	4146026	79	+	yijO/yijP	4145668	4146387	719	+	9.97	9.85	0.00
C0003	Tjaden et al.	8087	8159	72	-	yaaJ/talB	8075	8215	140	-	10.92	10.66	0.00
C1023	Tjaden et al.	4397780	4397851	71	-	miaA/hfq	4397218	4398275	1057	-	9.98	9.94	0.00
HB_260	Carter et al.	4456926	4457005	79	+	pmbA/cybC HB_261 4456966-4457045 + Resides within an operon	4456002	4457395	1393	+	11.12	10.93	0.00
C1027	Tjaden et al.	4399532	4399609	77	-	hflX/hflK	4399603	4400655	1052	-	10.19	9.99	0.00
HB_498	Carter et al.	3748678	3748757	79	-	sgbE/yiaT	3746241	3748844	2603	-	10.34	10.14	0.00
HB_99	Carter et al.	1582034	1582113	79	+	ydeO/ydeP	1582044	1582080	36	+	9.8	9.76	0.00
HB_98	Carter et al.	1577577	1577656	79	+	yddA/ydeM	1577518	1577611	93	+	10.01	9.75	0.01
HB_98	Carter et al.	1577577	1577656	79	+	yddA/ydeM	1577615	1577655	40	+	9.67	9.46	0.01
C0392	Tjaden et al.	1663181	1663259	78	+	ynfI/ynfJ	1656068	1664615	8547	+	10.73	10.56	0.00
HB_93	Carter et al.	1417576	1417655	79	+	ydaG/racR	1417534	1417579	45	+	9.69	9.47	0.01
HB_93	Carter et al.	1417576	1417655	79	+	ydaG/racR	1417597	1417655	58	+	10.59	10.44	0.00
HB_92	Carter et al.	1360573	1360652	79	+	ycjC/aldH	1360555	1360604	49	+	10.92	10.66	0.01
HB_92	Carter et al.	1360573	1360652	79	+	ycjC/aldH	1360602	1360660	58	+	11.01	10.94	0.00
HB_91	Carter et al.	1357436	1357513	77	+	ycjJ/ycjK	1357397	1357456	59	+	9.89	9.9	0.00
HB_91	Carter et al.	1357436	1357513	77	+	ycjJ/ycjK	1357455	1357574	119	+	9.81	9.82	0.00
HB_90	Carter et al.	1344937	1345001	64	+	yciR/rnb	1344924	1344973	49	+	10.08	10.12	0.00
HB_90	Carter et al.	1344937	1345001	64	+	yciR/rnb	1344977	1345214	237	+	10.24	10.25	0.00
HB_97	Carter et al.	1561231	1561310	79	+	yddT/yddU	1561228	1561299	71	+	9.17	9.24	0.00
HB_96	Carter et al.	1525417	1525496	79	+	yncH/rhsE	1525424	1525501	77	+	9.51	9.53	0.00
HB_95	Carter et al.	1489457	1489536	79	+	cybB/ydcA Overlaps a 3' UTR	1488895	1489549	654	+	11.61	11.58	0.00
HB_94	Carter et al.	1439259	1439338	79	+	Not intergenic	1439042	1439765	723	+	11.21	11.12	0.00
HB_481	Carter et al.	3616621	3616692	71	-	yhhG/rhsB	3615763	3617112	1349	-	10.52	10.6	0.00

HB_480	Carter et al.	3571208	3571287	79	-	glgB/asd Resides within an operon	3562046	3573072	11026	-	11.45	11.28	0.00
HB_483	Carter et al.	3655198	3655265	67	-	hdeD/yhiE C0857 3655198-3655608 -	3655188	3655406	218	-	10.34	10.04	0.01
HB_482	Carter et al.	3645948	3646027	79	-	gor/arsR	3645967	3646015	48	-	13.28	13.14	0.00
HB_485	Carter et al.	3662496	3662575	79	-	yhiW/yhiX HB_484 3662536-3662615 - Overlaps a 5' UTR	3661579	3662873	1294	-	12	11.62	0.00
HB_484	Carter et al.	3662536	3662615	79	-	yhiW/yhiX HB_485 3662496-3662575 -	3661579	3662873	1294	-	12	11.62	0.00
HB_487	Carter et al.	3697782	3697861	79	-	yhjU/yhjV HB_486 3697822-3697901 - C0868 3697742-3697968 -	3695865	3698030	2165	-	9.83	9.62	0.00
HB_486	Carter et al.	3697822	3697901	79	-	yhjU/yhjV HB_487 3697782-3697861 - C0868 3697742-3697968 -	3695865	3698030	2165	-	9.83	9.62	0.00
HB_489	Carter et al.	3697542	3697621	79	-	yhjU/yhjV HB_488 3697582-3697661 -	3695865	3698030	2165	-	9.83	9.62	0.00
HB_488	Carter et al.	3697582	3697661	79	-	yhjU/yhjV HB_489 3697542-3697621 -	3695865	3698030	2165	-	9.83	9.62	0.00
tpe27	Rivas et al.	2412694	?	?	unknown	ackA/pta Score: 13.99 Resides within an operon	2411266	2414837	3571	+	12.09	11.71	0.00
tpe27	Rivas et al.	2412694	?	?	unknown	ackA/pta Score: 13.99 Resides within an operon	2411404	2413791	2387	-	10.82	10.73	0.00
tpe20	Rivas et al.	1733277	?	?	unknown	ydhO/sodB Score: 14.61	1732387	1733348	961	+	10.9	10.8	0.00
tpe20	Rivas et al.	1733277	?	?	unknown	ydhO/sodB Score: 14.61	1732913	1733363	450	-	10.3	9.94	0.00
IS175	Chen et al.	3571214	3571405	191	+	glgB/asd C0832 3571143-3571363 +	3569462	3571553	2091	+	9.6	9.43	0.00
t60	Rivas et al.	3787757	?	?	unknown	yibD/tdh Score: 6.68	3787661	3788016	355	-	9.94	9.82	0.00
t63	Rivas et al.	3864532	?	?	unknown	ibpB/ibpA Score: 5.97 Resides within an operon	3864436	3864602	166	+	9.65	9.7	0.00
t63	Rivas et al.	3864532	?	?	unknown	ibpB/ibpA Score: 5.97 Resides within an operon	3864444	3864858	414	-	10.35	10	0.01
t62	Rivas et al.	1156799	?	?	unknown	ycfH/ptsG Score: 6.23	1145826	1156894	11068	+	12.48	12.28	0.00
t62	Rivas et al.	1156799	?	?	unknown	ycfH/ptsG Score: 6.23	1156378	1156894	516	-	11.5	11.37	0.00
HB_500	Carter et al.	3771822	3771880	58	-	mtlA/mtlD	3771104	3772099	995	-	9.92	9.8	0.00
HB_501	Carter et al.	3778712	3778791	79	-	lldD/yibK HB_502 3778672-3778751 -	3778694	3783132	4438	-	11.5	11.35	0.00
HB_502	Carter et al.	3778672	3778751	79	-	lldD/yibK HB_501 3778712-3778791 - HB_503 3778645-3778711 -	3778694	3783132	4438	-	11.5	11.35	0.00
HB_503	Carter et al.	3778645	3778711	66	-	lldD/yibK HB_502 3778672-3778751 -	3778694	3783132	4438	-	11.5	11.35	0.00
HB_504	Carter et al.	3809358	3809437	79	-	rpmB/radC Resides within an operon	3809214	3810055	841	-	13.95	13.69	0.00
HB_507	Carter et al.	3858856	3858935	79	-	yidL/glvG HB_506 3858896-3858975 - HB_508 3858816-3858895 -	3858773	3858859	86	-	9.7	9.48	0.01
HB_508	Carter et al.	3858816	3858895	79	-	yidL/glvG HB_507 3858856-3858935 -	3858773	3858859	86	-	9.7	9.48	0.01
HB_509	Carter et al.	3881715	3881794	79	-	dnaA/rpmH	3875578	3881988	6410	-	11.69	11.45	0.00
HB_138	Carter et al.	2458580	2458659	79	+	yfcY/yfcZ	2458562	2458695	133	+	9.92	9.83	0.00
HB_131	Carter et al.	2345211	2345290	79	+	nrdA/nrdB HB_132 2345251-2345330 + Resides within an operon	2342830	2347047	4217	+	10.92	10.92	0.00
HB_130	Carter et al.	2317899	2317978	79	+	rcsC/atoS	2316701	2317964	1263	+	9.83	9.83	0.00
HB_133	Carter et al.	2345291	2345353	62	+	nrdA/nrdB HB_132 2345251-2345330 + Resides within an operon	2342830	2347047	4217	+	10.92	10.92	0.00
HB_132	Carter et al.	2345251	2345330	79	+	nrdA/nrdB HB_133 2345291-2345353 + Resides within an operon HB_131 2345211-2345290 +	2342830	2347047	4217	+	10.92	10.92	0.00
HB_135	Carter et al.	2378529	2378608	79	+	Not intergenic C0536 2378534-2378740 + p26 2378544	2377336	2378691	1355	+	10.05	9.86	0.00
HB_134	Carter et al.	2360402	2360450	48	+	yfaX/yfaY	2360397	2360817	420	+	9.6	9.38	0.00
HB_137	Carter et al.	2438271	2438350	79	+	yfcJ/fabB	2438240	2438322	82	+	10.41	10.38	0.00
HB_137	Carter et al.	2438271	2438350	79	+	yfcJ/fabB	2438330	2438743	413	+	9.88	9.93	0.00
C0132	Tjaden et al.	607070	607166	96	+	Not intergenic HB_46 607097-607176 +	607124	608681	1557	+	10.8	10.68	0.00
C0989	Tjaden et al.	4237392	4237543	151	-	yjbH/yjbA HB_536 4237334-4237413 -	4237387	4237537	150	-	10.07	10.01	0.00
C0137	Tjaden et al.	613286	613363	77	-	Not intergenic	613219	613662	443	-	10.13	10.19	0.00
C0502	Tjaden et al.	2175319	2175434	115	+	gatY/fbaB	2175289	2176693	1404	+	10.22	10.12	0.00
HB_453	Carter et al.	3155494	3155573	79	-	yqhE/yqhG	3155259	3155608	349	-	9.59	9.51	0.00
C0506	Tjaden et al.	2213677	2213763	86	+	yehV/yehW	2213705	2214607	902	+	10.25	9.88	0.00
C0948	Tjaden et al.	4099036	4099156	120	-	sodA/kdgT	4099063	4099451	388	-	10.24	10.23	0.00
C0868	Tjaden et al.	3697742	3697968	226	-	yhjU/yhjV HB_487 3697782-3697861 - HB_486 3697822-3697901 -	3695865	3698030	2165	-	9.83	9.62	0.00
HB_279	Carter et al.	4576976	4577055	79	+	mcrB/yjiW HB_280 4577016-4577068 +	4576318	4577425	1107	+	10.01	9.83	0.00
C0861	Tjaden et al.	3661470	3661506	36	+	yhiV/yhiW HB_195 3661398-3661477 +	3657354	3661583	4229	+	10.18	9.71	0.00
tpk14	Rivas et al.	3450909	?	?	unknown	rpsJ/pioO Score: 11.37 Resides within an operon	3446293	3451317	5024	+	10.56	10.54	0.00
tpk14	Rivas et al.	3450909	?	?	unknown	rpsJ/pioO Score: 11.37 Resides within an operon	3437613	3451499	13886	-	13.48	13.29	0.00
C0865	Tjaden et al.	3673739	3673901	162	-	yhjE/yhjG	3673200	3674499	1299	-	10.4	10.19	0.00
HB_225	Carter et al.	4135328	4135407	79	+	yijF/gldA	4135305	4135357	52	+	10.35	10.22	0.00
HB_225	Carter et al.	4135328	4135407	79	+	yijF/gldA	4135399	4135467	68	+	9.82	9.65	0.00
HB_224	Carter et al.	4077790	4077869	79	+	yiiF/fdhE C0945 4077774-4077861 +	4077760	4077991	231	+	9.78	9.44	0.01
HB_348	Carter et al.	1328902	1328981	79	-	yciN/topA	1328970	1329022	52	-	9.27	9.02	0.01
HB_349	Carter et al.	1333645	1333724	79	-	yciX/acnA	1333610	1333764	154	-	10.14	9.98	0.00
HB_342	Carter et al.	1268332	1268411	79	-	kdsA/chaA HB_341 1268372-1268451 -	1268279	1269894	1615	-	10.22	10.03	0.01
HB_343	Carter et al.	1278597	1278676	79	-	narK/narG	1278540	1278691	151	-	10.64	10.64	0.00
HB_340	Carter et al.	1268532	1268611	79	-	kdsA/chaA HB_339 1268572-1268651 -	1268279	1269894	1615	-	10.22	10.03	0.01

HB_341	Carter et al.	1268372	1268451	79	-	kdsA/chaA HB_342 1268332-1268411 -	1268279	1269894	1615	-	10.22	10.03	0.01
HB_347	Carter et al.	1293409	1293488	79	-	tdk/ychG	1293384	1293505	121	-	10.09	9.97	0.00
HB_344	Carter et al.	1286681	1286760	79	-	tyrV/tyrT HB_345 1286641-1286720 -	1286686	1286748	62	-	12.66	11.42	0.03
HB_344	Carter et al.	1286681	1286760	79	-	tyrV/tyrT HB_345 1286641-1286720 -	1286748	1286972	224	-	10.61	10.45	0.00
HB_345	Carter et al.	1286641	1286720	79	-	tyrV/tyrT HB_344 1286681-1286760 - HB_346 1286602-1286680 -	1286686	1286748	62	-	12.66	11.42	0.03
HB_240	Carter et al.	4237470	4237549	79	+	yjbH/yjbA HB_239 4237430-4237509 +	4237198	4237545	347	+	10.08	9.97	0.00
IS214	Chen et al.	4371910	4372189	279	+	yjeJ/yjeK	4372044	4372098	54	+	9.22	9.12	0.00
HB_273	Carter et al.	4518157	4518236	79	+	yjhU/yjhF C1066 4518187-4518237 + HB_274 4518197-4518238 +	4518185	4518253	68	+	9.56	9.56	0.00
C0648	Tjaden et al.	2763425	2763497	72	+	yfjL/yfjM	2763382	2763859	477	+	10.54	10.35	0.00
C0430	Tjaden et al.	1860674	1860747	73	-	yeaA/gapA psrA6 1860608-1860782 -	1860678	1860728	50	-	9.62	9.59	0.00

Supplementary Table S5

Transcripts overlapping with the novel sRNA candidates by Shinhara *et al.* (2011)

All novel sRNA candidates by Shinhara *et al.* (2011) that overlap with differentially or similarly expressed regions detected by the sliding window method in this study are shown. Data in columns 1-5 are from Shinhara *et al.* while data in columns 6-13 were generated in this study. Fold changes are $\log_2$ values.

Shinhara ID	Shinhara Start	Shinhara End	Shinhara Length	Shinhara Strand	Overlap	This study Start	This study End	This study Length	This study Strand	Regulation	Fold change	Name in Fig 3 or Table 2
ECS002	3645971	3646021	51	-	45	3645967	3646015	49	-	Sim	-0.14	
ECS004	3646315	3646392	78	+	61	3646332	3646402	71	+	Sim	-0.62	
ECS005	3655821	3655882	62	+	14	3655786	3655834	49	+	Diff	-1.46	novel13
ECS005	3655821	3655882	62	+	25	3655858	3655942	85	+	Diff	-0.74	
ECS005	3655821	3655882	62	+	49	3655825	3655873	49	+	Sim	-0.51	
ECS006	2111172	2111260	89	-	57	2111204	2111290	87	-	Sim	-0.65	
ECS007	2066460	2066509	50	-	50	2066365	2066514	150	-	Sim	-0.22	
ECS008	2331923	2331997	75	+	74	2331924	2332085	162	+	Sim	-0.10	
ECS009	2468528	2468713	186	-	181	2468509	2468708	200	-	Sim	-0.62	
ECS011	2651730	2651800	71	-	71	2651717	2651848	132	-	Sim	-0.62	
ECS017	312026	312117	92	+	50	312068	312123	56	+	Sim	-0.18	
ECS018	3358818	3358907	90	-	90	3358758	3358907	150	-	Sim	-0.22	
ECS019	1702813	1702878	66	-	55	1702823	1702877	55	-	Sim	-0.36	
ECS020	1049757	1049935	179	-	60	1049875	1049934	60	-	Diff	-0.77	
ECS022	3646083	3646161	79	+	51	3646047	3646133	87	+	Diff	-1.60	novel6
ECS022	3646083	3646161	79	+	26	3646120	3646145	26	+	Diff	-1.03	
ECS024	407797	407870	74	+	50	407821	408199	379	+	Sim	0.04	
ECS026	2276439	2276488	50	-	46	2276432	2276484	53	-	Sim	-0.96	
ECS027	637846	637925	80	+	35	637872	637906	35	+	Diff	-0.74	
ECS030	502571	502645	75	+	67	502579	502681	103	+	Sim	0.04	
ECS038	3098842	3098893	52	+	43	3098851	3098900	50	+	Sim	-0.18	
ECS039	89360	89417	58	+	31	89387	89426	40	+	Diff	-0.75	
ECS039	89360	89417	58	+	8	89410	89450	41	+	Sim	-0.45	
ECS039	89360	89417	58	+	38	89284	89397	114	+	Sim	-0.42	
ECS041	4435508	4435575	68	-	38	4435538	4435578	41	-	Sim	0.10	
ECS049	1669841	1669894	54	-	4	1669891	1669954	64	-	Sim	-0.23	
ECS050	3886256	3886315	60	-	39	3886233	3886294	62	-	Sim	0.02	
ECS053	59499	59625	127	+	52	59477	59550	74	+	Diff	-0.73	nc6
ECS062	455812	455876	65	-	44	455793	455855	63	-	Sim	-0.45	
ECS074	585114	585202	89	+	29	585089	585142	54	+	Sim	-0.69	
ECS089	29303	29371	69	+	4	29368	29404	37	+	Sim	-0.59	
ECS103	62	132	71	-	42	7	103	97	-	Sim	-0.16	
ECS105	1733308	1733376	69	-	7	1733370	1733395	26	-	Diff	-1.11	
ECS106	2042726	2042792	67	-	45	2042748	2042862	115	-	Sim	-0.54	
ECS106	2042726	2042792	67	-	29	2042710	2042754	45	-	Sim	-0.01	
ECS111	2902502	2902556	55	-	42	2902456	2902543	88	-	Sim	-0.80	
ECS118	2066059	2066128	70	+	13	2066027	2066071	45	+	Sim	-1.33	
ECS118	2066059	2066128	70	+	64	2066057	2066122	66	+	Diff	-0.98	
ECS118	2066059	2066128	70	+	22	2066107	2066169	63	+	Sim	-0.65	
ECS119	4116145	4116213	69	+	12	4116202	4116247	46	+	Sim	-0.61	
ECS121	2967133	2967207	75	+	75	2967119	2967255	137	+	Sim	-0.37	
ECS127	4116322	4116371	50	+	36	4116331	4116366	36	+	Diff	-0.93	
ECS134	2111303	2111362	60	+	13	2111275	2111315	41	+	Sim	-0.18	
ECS137	4324995	4325051	57	+	19	4324950	4325013	64	+	Diff	-1.52	novel11
ECS138	4460897	4460952	56	+	18	4460935	4460975	41	+	Sim	0.03	

ECS151	1649586	1649648	63	-	63	1648707	1650705	1999	-	Sim	-0.31	
ECS152	2454057	2454119	63	-	46	2453944	2454102	159	-	Sim	-0.32	
ECS156	2454313	2454382	70	+	5	2454276	2454317	42	+	Sim	-0.28	
ECS160	2380661	2380730	70	-	8	2380573	2380668	96	-	Sim	-0.30	
ECS160	2380661	2380730	70	-	46	2380676	2380721	46	-	Sim	-0.11	
ECS167	989733	989783	51	+	11	989711	989743	33	+	Diff	-1.04	
ECS180	2404787	2404849	63	-	63	2404765	2404889	125	-	Diff	-0.68	
ECS184	2404734	2404785	52	-	21	2404765	2404889	125	-	Diff	-0.68	
ECS186	2576447	2576555	109	-	8	2576548	2576637	90	-	Sim	0.05	
ECS191	1019493	1019559	67	+	10	1019439	1019502	64	+	Sim	-0.33	
ECS200	2902008	2902058	51	+	46	2902013	2902106	94	+	Sim	-0.16	
ECS205	2559006	2559079	74	-	66	2558975	2559071	97	-	Sim	-0.31	
ECS207	3920625	3920699	75	+	12	3920688	3920795	108	+	Sim	-0.80	nc10
ECS207	3920625	3920699	75	+	71	3920629	3920702	74	+	Diff	-0.77	nc10
ECS207	3920625	3920699	75	+	20	3920532	3920644	113	+	Sim	0.01	nc10
ECS210	889150	889273	124	+	21	889094	889170	77	+	Sim	0.39	
ECS214	1755358	1755419	62	-	42	1755367	1755408	42	-	Sim	-1.68	
ECS214	1755358	1755419	62	-	27	1755329	1755384	56	-	Diff	-1.52	
ECS224	4056241	4056304	64	+	6	4056190	4056246	57	+	Sim	-0.35	
ECS229	4570223	4570288	66	+	66	4570141	4571307	1167	+	Sim	0.11	

Supplementary Table S6

Primer sequences for RT-qPCR: Differentially expressed genes

Gene	Forward Primer	Reverse Primer
ada	TGCGTACCACAGGCATCTTTT	TTTTCCCGCAAAGCATGTCT
aidB	CGCTCGAGGCGAATCG	TTTTTGTGCCGCGCTTTT
recN	TTTTTGTGCCGCGCTTTT	GACGGGCATGAATGTTTGCT
tisAB	TCATACGCGTCTCCTGTGGTT	AGGCAGCGGAAAGGTACGT
hmp	GCCGGTGTTGGTCAAACG	CTGTGTGGCCTGCTTTTGC
ytfE	GCTGATTCTGCAAGCGACTAAA	TTGGCACGCTCGGTTTG
cspH	GGTCCATATTTCCGCATTCACT	GGCAAAATTCCACACGTAATCC
gcvA	TTCAGCAAGGGCCAATTTTT	CCCTGCCCGTGGATAGC
pdhR	GCCCGGGCGAAAAACT	GGACGGGAGACGTCAAACTG
yfgH	TGCCGTAGACAACTCCCAAAA	GCGACTGCGCCAATAAGC

Supplementary Table S7

Primer sequences for RT-qPCR: Control genes

Gene	Forward Primer	Reverse Primer
rrsB	CGTGTTGTGAAATGTTGGGTTAA	CCGCTGGCAACAAAGGATAA (not shown in Figure 1)
alkA	CGTATGACTGGTCGTGGATGTT	CGCGACCGTTTCCACACT

Supplementary Table S8

Primer sequences for RT-qPCR: Differentially expressed novel transcripts (nc3-10)

Candidate No	Start Nucleotide	Stop Nucleotide	Strand	Forward Primer	Reverse Primer
irsB	1 985 809	1 986 130	-	GCCAGGCAATATGTTCCGATA	ACCGTCATCAGGACACAAAAAA
omrA/omrB	2 974 054	2 974 389	-	TGGTCTGCACGACGACTCTT	TGCATTTCCCTTCATTCCTTTG
nc3	2 773 147	2 773 512	+	AGCCAGCGGCATGGAA	CGGCACCATTTGCCTACAGT
nc4	4 532 242	4 532 559	+	AGGCAGATGACACTGACGAATG	CGAAGGGAAAAGCCGATGA
nc5	20 629	20 774	-	GCCGGTTGCCAGATAGTCA	CGATTCTTAAGCCACGAAGAGTTC
nc6	59 477	59 584	+	CGTTAAAATGAAATATAAGAGACGGTCAA	GTGTGGCTACGCTTTCCATTC
nc7	3 107 318	3 107 457	-	TCTCCTCTCTGTACGGAGTTTGC	GGCGATAAGCGAGAGAATGTAAG
nc8	4 457 461	4 457 805	+	GCCGCAGCGCTGGAT	CCGGTGATTTATCTTCGAGCTT
nc9	2 010 221	2 010 451	+	CCGGCTGAGTTTTTAACGAAA	CGCCCTGGGCAATATAATTTAT
nc10	3 920 629	3 920 830	+	TTCAGATCACATATTGCGCATGT	AAAGGCATCATTTGCCAAGTAAA
